# Evolution of structural diversity of trichothecenes, a family of toxins produced by plant pathogenic and entomopathogenic fungi

**DOI:** 10.1371/journal.ppat.1006946

**Published:** 2018-04-12

**Authors:** Robert H. Proctor, Susan P. McCormick, Hye-Seon Kim, Rosa E. Cardoza, April M. Stanley, Laura Lindo, Amy Kelly, Daren W. Brown, Theresa Lee, Martha M. Vaughan, Nancy J. Alexander, Mark Busman, Santiago Gutiérrez

**Affiliations:** 1 Mycotoxin Prevention and Applied Microbiology, National Center for Agricultural Utilization Research, U.S. Department of Agriculture, Peoria, Illinois, United States of America; 2 Area of Microbiology, University School of Agricultural Engineers, University of León, Campus de Ponferrada, Ponferrada, Spain; 3 Microbial Safety Team, National Institute of Agricultural Sciences, Rural Development Administration, Wanju, Republic of Korea; UW-Madison, Madison, WI 53706 USA, UNITED STATES

## Abstract

Trichothecenes are a family of terpenoid toxins produced by multiple genera of fungi, including plant and insect pathogens. Some trichothecenes produced by the fungus *Fusarium* are among the mycotoxins of greatest concern to food and feed safety because of their toxicity and frequent occurrence in cereal crops, and trichothecene production contributes to pathogenesis of some *Fusarium* species on plants. Collectively, fungi produce over 150 trichothecene analogs: i.e., molecules that share the same core structure but differ in patterns of substituents attached to the core structure. Here, we carried out genomic, phylogenetic, gene-function, and analytical chemistry studies of strains from nine fungal genera to identify genetic variation responsible for trichothecene structural diversity and to gain insight into evolutionary processes that have contributed to the variation. The results indicate that structural diversity has resulted from gain, loss, and functional changes of trichothecene biosynthetic (*TRI*) genes. The results also indicate that the presence of some substituents has arisen independently in different fungi by gain of different genes with the same function. Variation in *TRI* gene duplication and number of *TRI* loci was also observed among the fungi examined, but there was no evidence that such genetic differences have contributed to trichothecene structural variation. We also inferred ancestral states of the *TRI* cluster and trichothecene biosynthetic pathway, and proposed scenarios for changes in trichothecene structures during divergence of *TRI* cluster homologs. Together, our findings provide insight into evolutionary processes responsible for structural diversification of toxins produced by pathogenic fungi.

## Author summary

Toxins produced by pathogens can contribute to infection and/or colonization of hosts. Some toxins consist of a family of metabolites with similar but distinct chemical structures. This structural variation can affect biological activity, which in turn likely contributes to adaptation to different environments, including to different hosts. Trichothecene toxins consist of over 150 structurally distinct molecules produced by certain fungi, including some plant and insect pathogens. In multiple systems that have been examined, trichothecenes contribute to pathogenesis on plants. To elucidate the evolutionary processes that have given rise to trichothecene structural variation, we conducted comparative analyses of nine fungal genera, most of which produce different trichothecene structures. Using genomic, molecular biology, phylogenetic, and analytical chemistry approaches, we obtained evidence that trichothecene structural variation has arisen primarily from gain, loss, and functional changes of trichothecene biosynthetic genes. Our results also indicate that some structural changes have arisen independently in different fungi. Our findings provide insight into genetic and biochemical changes that can occur in toxin biosynthetic pathways as fungi with the pathways adapt to different environmental conditions.

## Introduction

Secondary metabolites (SMs) are low-molecular-weight metabolites that are not required for growth or development, but instead provide ecological advantages under certain environmental conditions. Microbial SMs are diverse in chemical structure and biological activity; some are toxins, plant hormones, pigments, or antibiotics, and some have pharmaceutical properties. Many SMs contribute to host-pathogen interactions. Despite their structural diversity, most microbial SMs are derived from one of three classes of parent compounds: non-ribosomal peptides, polyketides, and terpenes [1]. SM structural diversity results from functional variation in enzymes that synthesize the parent compounds (i.e., non-ribosomal peptide synthetases, and polyketide and terpene synthases) as well as enzymes that catalyze modifications of the parent compound. The latter enzymes include acyltransferases, amino transferases, dehydrogenases, reductases, dioxygenases, methyltransferases, monooxygenases, and prenyltransferases. In fungi, genes encoding enzymes required for synthesis of the same SM are typically located adjacent to one another in a biosynthetic gene cluster [2]. Such clusters can also encode transport proteins that export SMs from cells, and transcription factors that activate expression of cluster genes. SMs often consist of families of analogs that share a core structure, but vary in the presence of substituents (functional groups) attached to the core structure. Structural variation among analogs of the same SM family typically results from the presence, absence, or differences in function of genes encoding modifying enzymes [2,3].

Trichothecenes are a family of toxic SMs produced by some, but not all, species in multiple fungal genera, including *Fusarium*, *Isaria*, *Microcyclospora*, *Myrothecium*, *Peltaster*, *Spicellum*, *Stachybotrys*, *Trichoderma*, and *Trichothecium* [4–8]. Most known trichothecene-producing fungi are plant pathogens, and one, *Isaria tenuipes*, is an insect pathogen [5]. In *Fusarium*, trichothecene production contributes to pathogenesis on multiple crop plants [9–11], and some *Fusarium* trichothecenes are among the mycotoxins of greatest concern to food and feed safety [12]. In addition, *Stachybotrys* trichothecenes have been implicated in negative health effects of mold growth in damp buildings [13]. In contrast, trichothecene production by *Trichoderma arundinaceum* contributes to its biological control activity against some plant pathogenic fungi [14].

**Fig 1:**
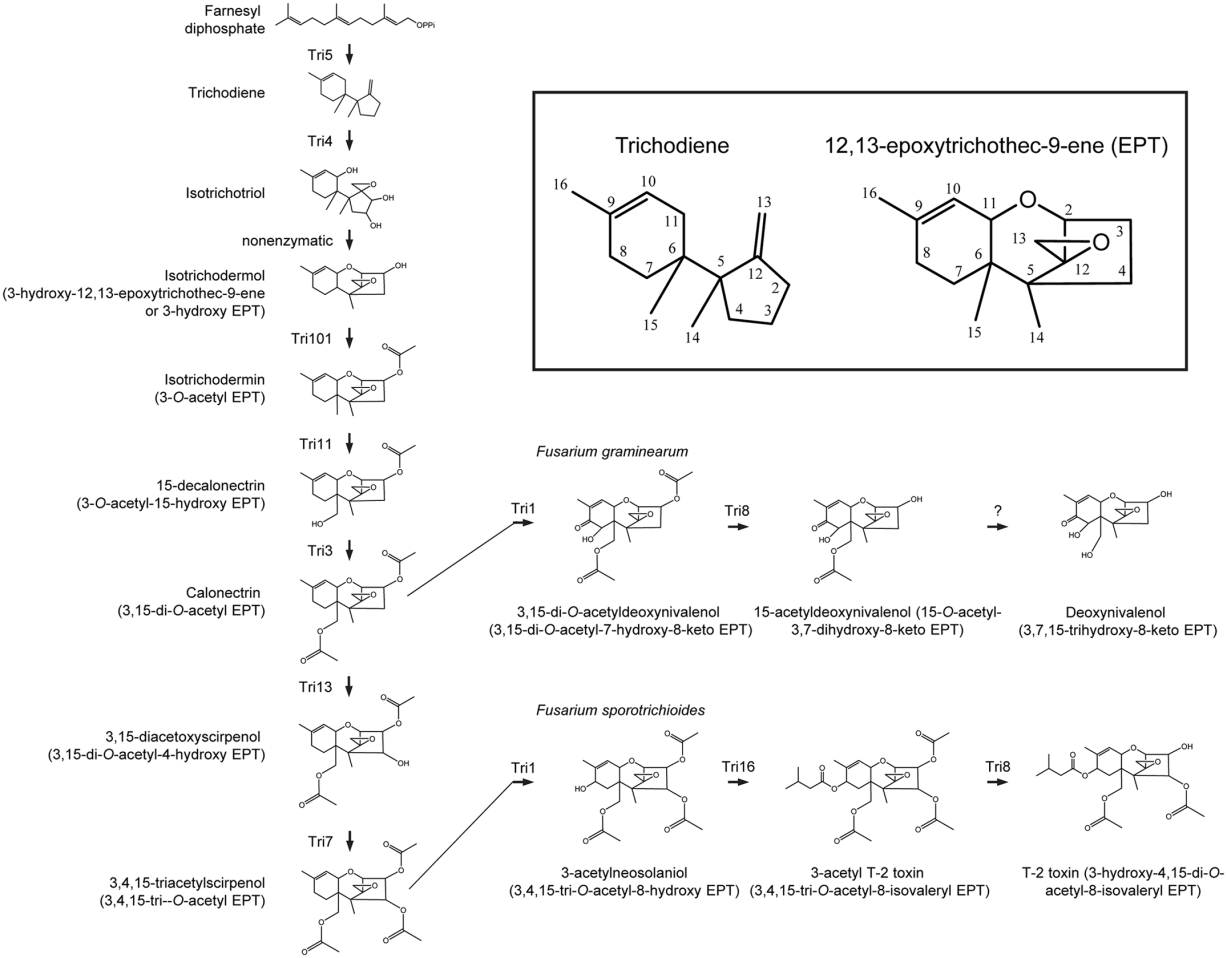
Biosynthetic pathways for the trichothecene analogs deoxynivalenol and T-2 toxin in *Fusarium graminearum* and *F*. *sporotrichioides*, respectively. The inset at the top right shows the structure and numbering systems for trichodiene and 12,13-epoxytrichothec-9-ene (EPT).

**Fig 2:**
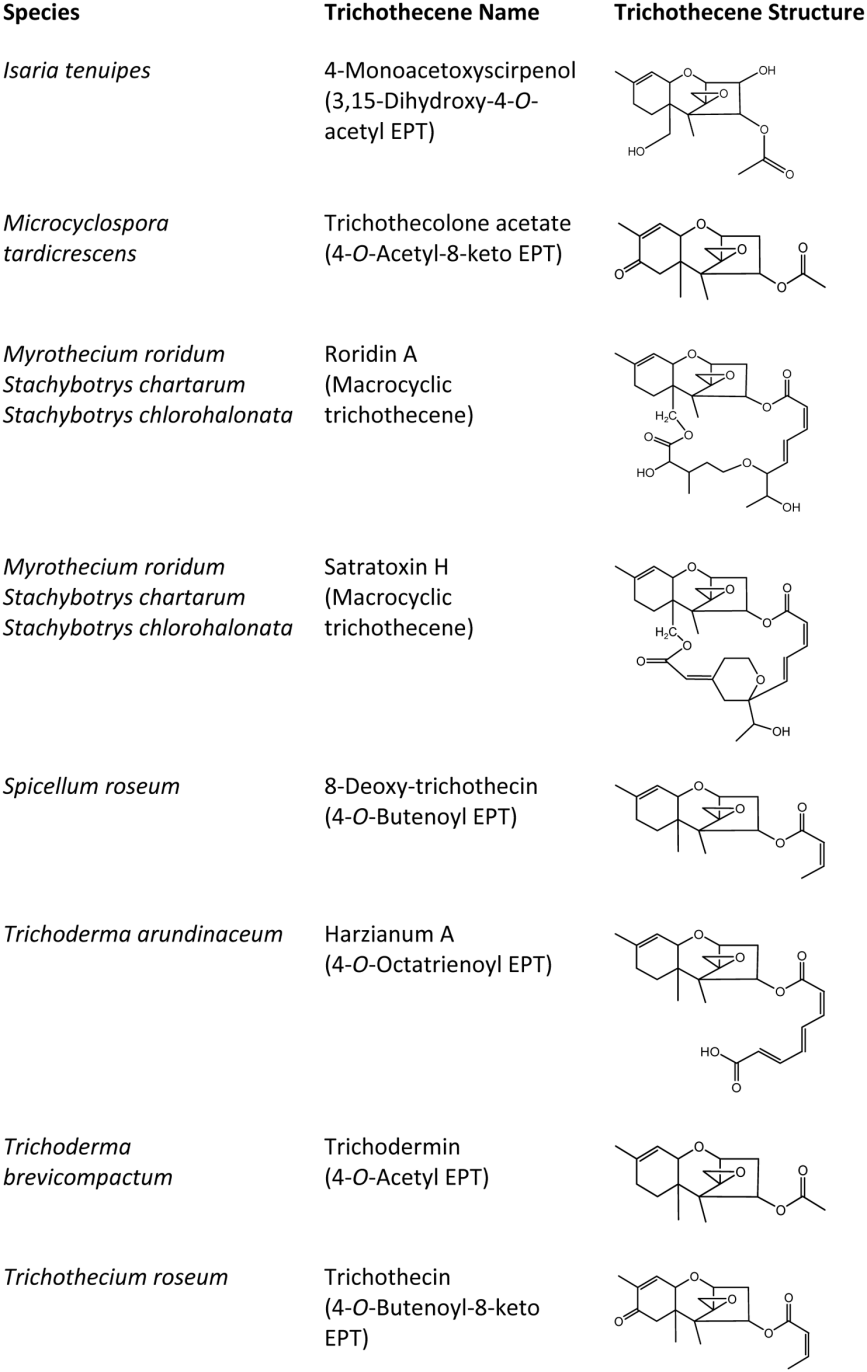
Representative diversity of trichothecene structures in fungi other than *Fusarium*. See Fig 1 for examples of *Fusarium* trichothecenes.

The core structure of trichothecenes consists of a three-ring molecule known as 12,13-epoxytrichothec-9-ene (EPT; Fig 1), and analogs of trichothecenes differ from one another in the patterns of substituents attached to EPT (Fig 2). One type of structural variation has resulted in classification of trichothecenes into two groups [15]. Analogs in the first group, macrocyclic trichothecenes, have a macrolide ring resulting from a 12 or 14-atom chain esterified via hydroxyl groups at carbon atoms 4 and 15 (C4 and C15) of EPT. Analogs in the second group, simple trichothecenes, lack a macrolide ring.

The genetics and biochemistry of trichothecene biosynthesis have been studied most extensively in *Fusarium*, and biosynthetic pathways for *Fusarium* trichothecenes that significantly impact agriculture have been elucidated (e.g., deoxynivalenol, nivalenol and T-2 toxin; Fig 1) [3,16]. Additional studies indicate that at least the initial steps in trichothecene biosynthesis are similar in *Fusarium*, *Myrothecium* and *Trichoderma* [17–20]. Trichothecene biosynthesis begins with the cyclization of the primary metabolite farnesyl diphosphate to form the terpene trichodiene. This reaction is catalyzed by a terpene synthase (trichodiene synthase). Subsequently, a cytochrome P450 monooxygenase (trichodiene oxygenase) catalyzes oxygenation of trichodiene at three or four positions to yield isotrichodiol or isotrichotriol, which can cyclize nonenzymatically to form EPT or 3-hydroxy EPT (isotrichodermol), respectively. These latter molecules undergo one or more additional oxygenations, acylations and sometimes other modifications to form all trichothecene analogs [4].

The trichothecene biosynthetic gene (*TRI*) cluster is one of the most studied SM gene clusters in fungi. Homologs of the *TRI* cluster have been identified in *Fusarium*, *Stachybotrys*, and *Trichoderma* [3,16,17,21]. In addition, sequence analysis of a *Myrothecium roridum* cosmid clone identified three adjacent *TRI* genes presumed to be part of a larger cluster [22], and RNAseq analysis of the fungus has identified homologs of six *TRI* genes [23]. The number of *TRI* genes per cluster varies among *Fusarium*, *Stachybotrys*, and *Trichoderma* and in some cases among species of the same genus. The *Fusarium* and *Stachybotrys* cluster homologs include the trichodiene synthase gene (*TRI5*), the trichodiene oxygenase gene (*TRI4*), two regulatory genes (*TRI6* and *TRI10*), and other genes encoding enzymes that catalyze addition of substituents to the core EPT structure (Table 1). The *Trichoderma TRI* cluster differs in that it lacks *TRI5*, which is located elsewhere in the genome [17]. *Fusarium* and *Stachybotrys* also have *TRI* genes at loci other than the *TRI* cluster. In some *Fusarium* species, monooxygenase (*TRI1*) and acyltransferase (*TRI16*) genes are at a second locus, and an acetyltransferase gene (*TRI101*) is at a third locus [3]. In other *Fusarium* species, however, *TRI1* and *TRI101* are located in the cluster [24]. In *Stachybotrys*, *TRI* genes at loci other than the cluster are paralogs of genes in the cluster [21].

**Table 1:** Functions of trichothecene biosynthetic genes.

Gene^a^	Functional Category^b^	Function in trichothecene biosynthesis^c^
*TRI3*	acetyl transferase	acetylation at C15 (*Fusarium*)
*TRI4*	cytochrome P450 monooxygenase	oxygenation of trichodiene at C2, C11, and C13 (*Myrothecium*, *Trichoderma*), or C2, C3, C11, and C13 (*Fusarium*)
*TRI5*	terpene synthase	cyclization of farnesyl pyrophosphate to trichodiene (*Fusarium*, *Trichoderma*)
*TRI6*	Zn_2_His_2_ transcription factor	transcriptional regulation of *TRI* gene expression (*Fusarium*)
*TRI7*	acetyl transferase	acetylation at C4 (*Fusarium*)
*TRI8*	esterase	deacetylation at C3 or C15 (*Fusarium*)
*TRI9*	unknown	unknown
*TRI10*	transcriptional regulator	transcriptional regulation of *TRI* gene expression (*Fusarium*)
*TRI11*	cytochrome P450 monooxygenase	hydroxylation at C15 (*Fusarium*)
*TRI12*	major facilitator superfamily transporter	trichothecene efflux pump (*Fusarium*)
*TRI13*	cytochrome P450 monooxygenase	hydroxylation at C4 (*Fusarium*)
*TRI14*	unknown	unknown; not required for synthesis in culture (*Fusarium*)
*TRI16*	acyl transferase	acylation at C8 (*Fusarium*)
*TRI17*	polyketide synthase	synthesis of polyketide esterified at C4 (predicted in *Stachybotrys*)
*TRI18*	acyl/acetyl transferase	unknown
*TRI19*	terpene synthase	*TRI5* paralog
*TRI22*^d^	cytochrome P450 monooxygenase	hydroxylation at C4 (*Trichoderma*)
*TRI101*	acetyl transferase	acetylation at C3 (*Fusarium*)

^**a**^ In literature on trichothecene biosynthetic genes, wild-type genes are variously designated as *TRI*, *Tri* or *tri*. For consistency among fungi examined in the current study, we have used the same format for all fungi. Uppercase italicized letters are used to indicate wild-type genes (e.g., *TRI5*), lowercase italicized letters are used to indicate inactivated genes (e.g., *tri5*), and non-italicized letters, with the first letter uppercase and the second and third letters lowercase, are used to indicate proteins (e.g., Tri5).

^**b**^ Functional categories are based on previously reported BLAST analyses.

^**c**^ Functions in trichothecene biosynthesis have been determined by chemical analysis of fungal strains in which the gene has been inactivated or by heterologous expression [3,16,17,25]. The fungal genus names in parentheses indicate the origin of the *TRI* homologs used in functional analyses.

^**d**^ In the initial characterization of *TRI22*, it was designated as *TRI11*. But here, we propose that it be re-designated as *TRI22* (S1 Fig).

Functional analyses of *TRI* genes have elucidated the genetic bases for much of the structural diversity of trichothecene analogs produced by *Fusarium* [3,16,26]. However, the genetic bases for most of the structural diversity of trichothecenes produced by other fungi are not known. For example, *T*. *arundinaceum* produces harzianum A, a trichothecene with a polyketide-derived side chain [6,27]. The macrolide ring of macrocyclic trichothecenes is thought to be composed of both polyketide- and isoprenoid-derived moieties [15]. Although the genes responsible for formation of these substituents have not been identified, a polyketide synthase (PKS) gene is located in the *TRI* cluster of *Stachybotrys* species that produce macrocyclic trichothecenes [21].

The objective of the current study was to investigate variation of *TRI* genes among selected fungi in order to identify evolutionary processes that have likely contributed to structural diversity of trichothecene analogs produced by different fungi. To this end, we used genome sequencing to compare the gene content, arrangement, and sequences of *TRI* loci in selected species of nine genera. We also conducted additional functional analyses of selected *TRI* genes. The results indicate that gain, loss, and changes in function of genes are major contributors to structural diversity of trichothecenes. We used the results to infer an ancestral trichothecene biosynthetic pathway and to propose scenarios for gain and loss of trichothecene substituents during divergence of *TRI* cluster homologs. Together, our findings and inferences provide insights into the evolutionary processes that have given rise to biochemical diversity in plant pathogenic, entomopathogenic, and other fungi.

## Results

### Genomic analysis and *TRI*-gene content

We used genome sequence data to examine the content and arrangement of *TRI* genes in 20 fungal strains that included 14 species from nine genera (Table 2). Genome sequence data for 12 strains were generated during the current study, while data for eight strains were generated in previous studies. The strains represented fungi with a range of lifestyles, including saprophytism, endophytism, plant pathogenicity, and entomopathogenicity. The two entomopathogenic fungi, *Beauveria bassiana* and *Cordyceps confragosa* (*Lecanicillium lecanii*), have *TRI* genes but have not been reported to produce trichothecenes as far as we are aware. To assess *TRI* gene content in the fungi, we used coding region sequences of the 18 previously described *TRI* genes (Table 1) as queries in BLASTn and BLASTx analyses against genome sequence databases of the 20 fungi.

**Table 2:** Information on fungal strains and genome sequences examined in the current study.

Species ^a^	Strain	Lifestyle	GenBank Accession No.^c^	Genome Size (Mb)	No. Contigs	No. Genes	N50
*Beauveria bassiana*	ARSEF 2860	Insect pathogen/ endophyte	ADAH00000000	33.7	1,229	10,364	84,720
*Cordyceps confragosa*	RCEF 1005	Insect pathogen	AZHF00000000	35.6	197	11,030	782,161
*Cordyceps confragosa*	UM487	Insect pathogen	LUKN00000000	32.6	8,204	8,126	9,866
FIESC 12^b^	NRRL 13405	Plant pathogen	PXXK00000000†	39.6	1,073	13,092	112,688
*Fusarium graminearum*	PH-1	Plant pathogen	AACM00000000	36.6	435	13,313	184,591
*Fusarium longipes*	NRRL 20695	Plant pathogen	PXOG00000000†	35.3	544	11,461	144,380
*Fusarium sporotrichioides*	NRRL 3299	Plant Pathogen	PXOF00000000†	37.4	446	12,014	235,034
*Microcyclospora tardicrescens*	HJS 1936	Plant pathogen	PXOE00000000†	26.2	1,069	10,375	169,506
*Myrothecium roridum*	NRRL 2183	Plant pathogen	PXOD00000000†	45.1	2,310	14,215	54,581
*Spicellum ovalisporum*	DAOM 186447	Saprophyte	PXOC00000000†	32.1	1,632	9,497	91,139
*Spicellum roseum*	DAOM 209012	Saprophyte	PXOB00000000†	34.1	1,154	10,426	168,957
*Stachybotrys chartarum*	IBT 7711	Saprophyte	APIU00000000	36.9	3,848	11,530	55,709
*Stachybotrys chartarum*	IBT 40288	Saprophyte	AQPQ00000000	36.0	3,659	11,368	46,546
*Stachybotrys chartarum*	IBT 40293	Saprophyte	ASEQ00000000	36.5	4,267	11,453	60,116
*Stachybotrys chlorohalonata*	IBT 40285	Saprophyte	APWP00000000	34.4	5,591	10,706	47,022
*Trichoderma arundinaceum*	IBT 40837 (Ta37)	Saprophyte	PXOA00000000†	36.9	1,370	10,539	134,831
*Trichoderma brevicompactum*	IBT 40841 (Tb41)	Saprophyte	PXNZ00000000†	37.0	1,404	10,467	57,298
*Trichothecium roseum*	DAOM 195227	Saprophyte/Plant pathogen	PXNY00000000†	33.9	1,466	9,759	77,986
*Trichothecium roseum*	DAOM 197141	Saprophyte/Plant pathogen	PXNX00000000†	32.2	4,816	10,007	13,428
*Trichothecium roseum*	K7-1	Saprophyte/Plant pathogen	PXNW00000000†	31.6	2,463	9,356	24,156

^**a**^ Fungi were obtained from the following individuals and institutions: *Beauveria*—Richard Humber, Agriculture Research Service Collection of Entomopathogenic Fungal Cultures (ARSEF); *Cordyceps*—Timothy James, Biology Department at the University of Michigan; *Fusarium* and *Myrothecium*—Agriculture Research Service (NRRL) Culture Collection, National Center for Agricultural Utilization Research, U.S. Department of Agriculture; *Microcyclospora*—Hans Josef Schroers at Agricultural Institute of Slovenia; *Spicellum* and *Trichothecium* (DAOM strains)—Keith A. Seifert, Ottawa Research and Development Centre, Agriculture and Agri-Food Canada; *Trichoderma*—Ulf Thrane, IBT Culture Collection of Fungi, Mycology Group, Technical University of Denmark; *Trichothecium* (strain K7-1)—Anne E. Desjardins, National Center for Agricultural Utilization Research, U.S. Department of Agriculture.

^**b**^ FIESC 12 is phylogenetic species 12 of the *Fusarium incarnatum*-*equiseti* species complex [28].

^**c**^ Accession numbers for genome sequences generated during the course of the current study are denoted with the symbol †. Data for all other strains were obtain from the NCBI website, and have been described previously [21,29–31].

**Fig 3:**
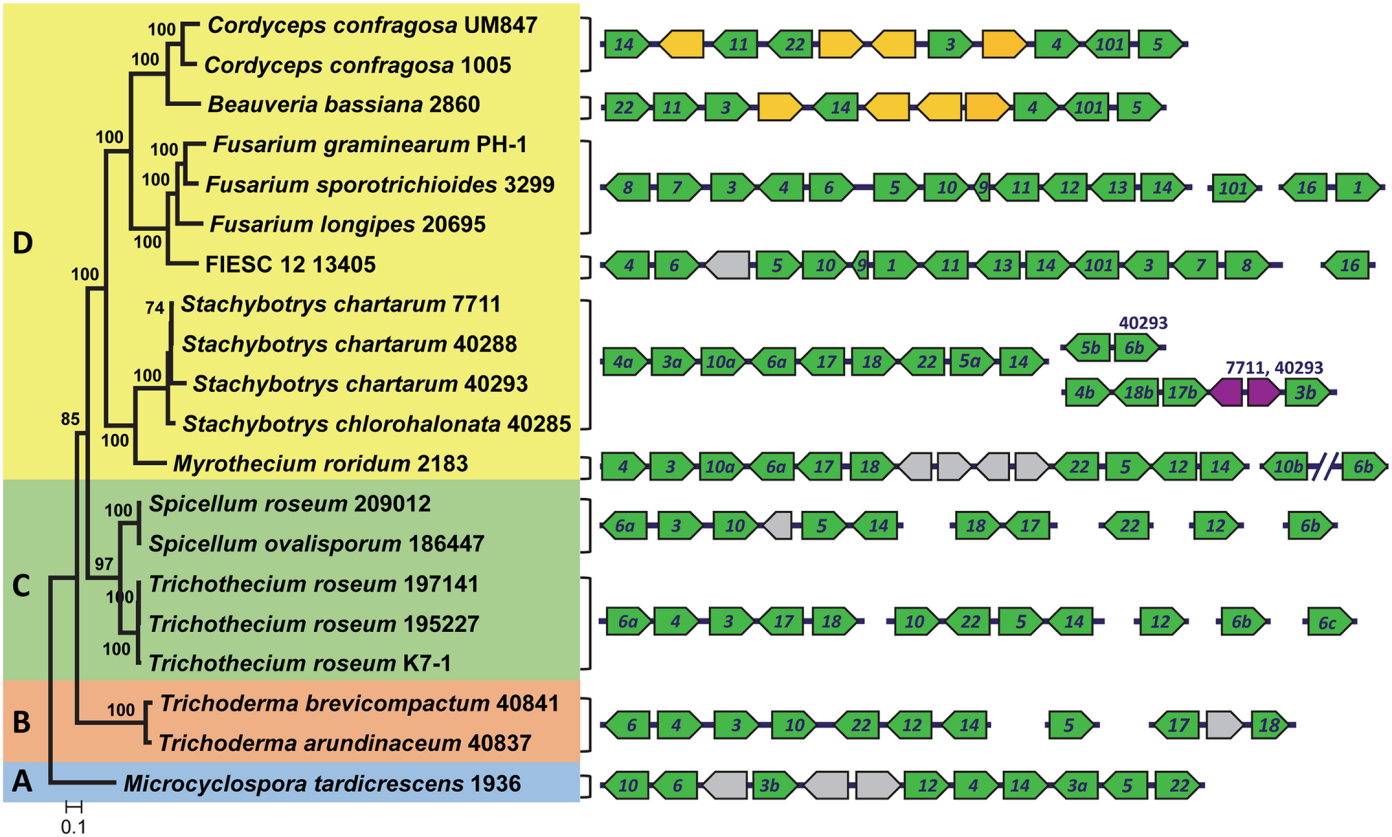
Gene content and arrangement at *TRI* loci in fungi examined in this study. The phylogenetic tree to the left was inferred by maximum likelihood analysis of concatenated nucleotide sequences of *TRI3*, *TRI5*, and *TRI14*, the only three *TRI* genes common to all the fungi. Numbers near branch nodes are bootstrap values based on 1000 pseudoreplicates. The four colored blocks (labeled A, B, C and D) indicate four lineages of *TRI* genes. The diagrams to the right show the content and arrangement of genes at *TRI* loci. Green arrows represent homologs of previously described *TRI* genes, and numbers within arrows indicate *TRI* gene designations (e.g., *14* indicates *TRI14*). *TRI22* was originally described as *TRI11* in *Trichoderma*, but here we consider *TRI11* and *TRI22* to be functionally and phylogenetically distinct genes (S1 Fig). For the purposes of this study, paralogs are indicated by the lowercase letters *a*, *b*, and *c*; e.g., *TRI6* paralogs are indicated as *6a*, *6b*, and *6c*. Gray arrows represent genes present in the *TRI* cluster of only one genus; orange arrows represent genes unique to *Beauveria* and *Cordyceps*; and purple arrows represent genes unique to *Stachybotrys* and *Myrothecium* (S2 Fig). Arrows overlaid on the same line indicate genes on the same contig, whereas arrows overlaid on different lines indicate genes on different contigs. For *Stachybotrys*, the numbers 7711 and 40293 above *TRI* paralogs indicate strains in which the paralogs occur. *TRI3b* in *S*. *chartarum* strain 40293 is truncated relative to other *TRI3* homologs and, as a result, is likely nonfunctional.

There was considerable variation in the presence and absence of *TRI* genes among the fungi examined (Fig 3, Table 3). The number of *TRI* genes per genome varied from six in *B*. *bassiana* and *C*. *confragosa* to 15 in *Fusarium sporotrichioides* and *Stachybotrys chartarum* strain 40293. The number of *TRI* genes varied within some genera and species as well. For example, *S*. *chartarum* had from nine to 15 *TRI* genes, because some *TRI* genes were duplicated in two strains [21]. *TRI3*, *TRI5* and *TRI14* were the only *TRI* genes that were present in all 20 fungi examined, while *TRI4* was present in all the fungi except the two *Spicellum* strains (Table 3). In some cases, *TRI*-gene counts per genome included two or three paralogs of the same gene (Table 3). We identified paralogs of the structural genes *TRI3*, *TRI4*, *TRI5*, *TRI17* and *TRI18* and the regulatory genes *TRI6* and *TRI10* (Table 3). *TRI6* had the largest number of paralogs; two each in *Myrothecium*, *Spicellum*, and *S*. *chartarum* 40293, and three in each *Trichothecium* strain (Table 3). *S*. *chartarum* 40293 had the largest number of *TRI* paralogs, with over half of the *TRI* genes in this strain being paralogs.

**Table 3:** *TRI* gene content of fungi examined in the current study. A gray box indicates that a *TRI* gene is present in the genome of a fungus, while a white box indicates the gene was not detected. Numbers within boxes indicate the number of paralogs. The Greek letter ψ indicates that a large portion of the gene is present but that it is a pseudogene.

Species	Strain	No. Loci^a^	No. *TRI* Genes	*TRI* Gene																	
				*1*	*3*	*4*	*5*	*6*	*7*	*8*	*9*	*10*	*11*	*12*	*13*	*14*	*16*	*17*	*18*	22^b^	*101*
*Beauveria bassiana*	ARSEF 2860	1	6																		
*Cordyceps confragosa*	RCEF 1005	1	6																		
*Cordyceps confragosa*	UM487	1	6																		
FIESC 12	NRRL 13405	2	14																		
*Fusarium graminearum*	PH-1	3	12						Ψ						Ψ		Ψ				
*Fusarium longipes*	NRRL 20695	3	15																		
*Fusarium sporotrichioides*	NRRL 3299	3	15																		
*Microcylospora tardicrescens*	HJS 1936	1	11		2																
*Myrothecium roridum*	NRRL 2183	2	13					2													
*Spicellum ovalisporum*	DAOM 186447	nd	10					2													
*Spicellum roseum*	DAOM 209012	nd	10					2													
*Stachybotrys chartarum*	IBT 7711	2	12			2												2	2		
*Stachybotrys chartarum*	IBT 40288	1	9																		
*Stachybotrys chartarum*	IBT 40293	3	14			2	2	2										2	2		
*Stachybotrys chlorohalonata*	IBT 40285	1	9																		
*Trichoderma arundinaceum*	IBT 40837	3	10																		
*Trichoderma brevicompactum*	IBT 40841	3	10																		
*Trichothecium roseum*	DAOM 195227	nd	12					3													
*Trichothecium roseum*	DAOM 197141	nd	12					3													
*Trichothecium roseum*	K7-1	nd	12					3													

^**a**^ nd indicates that sequence assemblies do not provide a clear indication of the number of loci.

^**b**^ *TRI22* was originally described as the *Trichoderma* homolog of the *Fusarium TRI11* [17]. But here, we consider it functionally and phylogenetically distinct from *TRI11* (S1 Fig).

The sequence data also indicated that *TRI* genes can occur at one to as many as five distinct genomic locations (loci). In some fungi with multiple *TRI* loci, genes located at different loci were paralogs. For example, the *Myrothecium* and *Stachybotrys TRI* clusters include nine and ten known *TRI* genes, respectively, but the *TRI* genes at other loci were paralogs of genes in the cluster. In *Fusarium* and *Trichoderma*, by contrast, *TRI* genes occurred at two or three loci, but the gene at the same or different loci in these fungi were not paralogous.

In the *Spicellum* and *Trichothecium* strains examined, *TRI* genes were dispersed over five or six contigs (S2 Fig). Although this dispersion of *TRI* genes on different contigs was likely an artifact of the genome sequence assembly in some cases, in other cases it was not artefactual. In *S*. *roseum*, for example, *TRI12* was near the middle of 243-kb contig and *TRI3*, *TRI5*, *TRI6a*, *TRI10*, and *TRI14* were located adjacent to one another and near the middle of a 169-kb contig (S2 Fig). In both of these contigs, the *TRI* genes were flanked by multiple genes that were unlikely to be involved in trichothecene biosynthesis based on their predicted functions. Thus, like *Fusarium*, *Myrothecium*, *Stachybotrys*, and *Trichoderma*, *TRI* genes in *Spicellum* and *Trichothecium* occur at two or more loci (S2 Fig).

The apparent absence of *TRI4* in the *Spicellum* genome sequences was unexpected, because *TRI4* is required for essential steps that occur early in trichothecene biosynthesis in other fungi [3,16]. We used three approaches to determine whether the absence of *TRI4* was a sequencing or assembly artifact: 1) generation of genome sequence data for both strains of *Spicellum* using two or three Methods (MiSeq, TruSeq, and Ion Torrent); 2) RNAseq analysis of *S*. *roseum* (strain 209012) grown under conditions that induced expression of other *TRI* genes; and 3) PCR analysis of the *Spicellum* strains using multiple primer pairs that amplified *TRI4* fragments from *Fusarium*, *Myrothecium* and *Trichothecium* strains. None of these methods yielded evidence for a full-length *TRI4* homolog in either *Spicellum* strain. However, BLASTx analysis of the *TRI3*-*TRI6a* intergenic region in *S*. *roseum* 209012 revealed a 558-nucleotide sequence that is likely a remnant of *TRI4* (S3 Fig).

The absence of *TRI4* in both *Spicellum* strains led us to predict that neither strain would produce trichothecenes. In Gas chromatography-mass spectrometry (GC-MS) analysis, we did not detect trichothecenes in culture extracts of *S*. *ovalisporum*, but we did detect them in culture extracts of *S*. *roseum* strain 209012 (S4 Fig). Consistent with a previous study [32], the most abundant trichothecene analog produced was 8-deoxy-trichothecin (4-*O*-butenoyl EPT). The absence of a *TRI4* homolog in *S*. *roseum* suggests that it must have a gene(s) that encodes another trichodiene oxygenase. Attempts to identify such a gene by RNAseq analysis were not successful. That is, we did not find evidence for an oxygenase gene in *S*. *roseum* 209012 that exhibited a pattern of expression similar to those of known *TRI* genes.

### Functional analyses of *TRI* genes

#### Trichoderma arundinaceum TRI3

**Fig 4:**
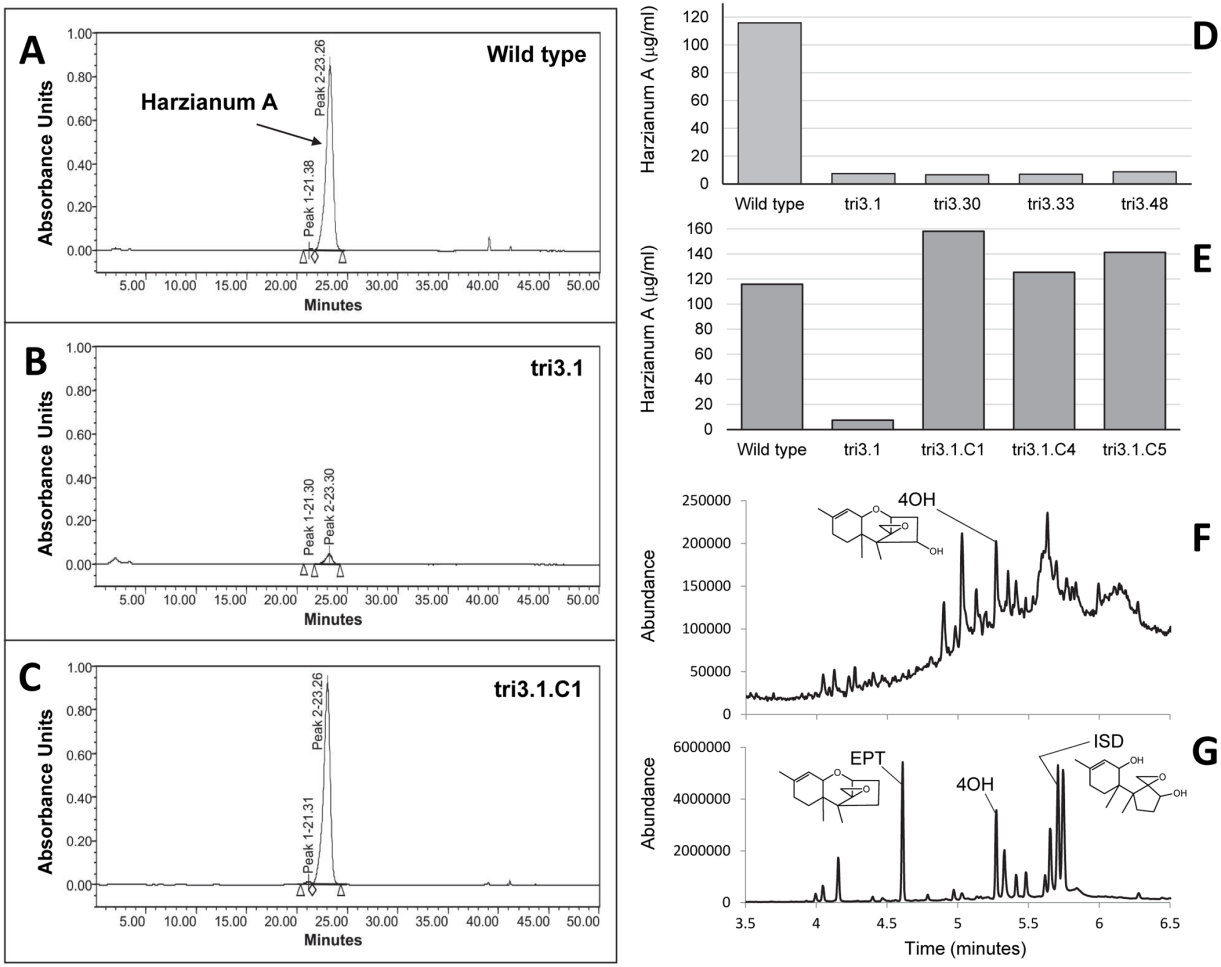
Functional analysis of *TRI3* in *Trichoderma arundinaceum*. (**A-C**) High performance liquid chromatograms showing harzianum A (HA) production by: (**A**) wild-type progenitor strain Ta37; (**B**) *tri3* mutant strain tri3.1; and (**C**) strain tri3.1.C1, *tri3* mutant strain tri3.1 complemented with a wild-type copy of *TRI3*. (**D**) Quantification of HA production in the wild type and *tri3* deletion mutant strains tri3.1, tri3.30, tri3.33, and tri3.48. (**E**) Quantification of HA production in the wild type, *tri3* mutant strain tri3.1, and three tri3.1-derived strains that were complemented with a wild-type copy of *TRI3* (strains tri3.1.C1, tri3.1.C4 and tri3.1.C5). (**F** and **G**) Total ion chromatograms from gas chromatography-mass spectrometry analysis of culture extracts of the (**F**) wild-type strain and (**G**) *tri3* mutant strain tri3.1. The peaks labeled 4OH and ISD are for trichodermol (4-hydroxy EPT) and isotrichodiol, respectively.

The presence of *TRI3* in fungi that produce trichothecenes that lack a C15 ester suggests that the gene can have a function other than the C15 esterification function previously described in *Fusarium* [33]. To determine whether this is the case, we inactivated *TRI3* in *T*. *arundinaceum* by gene deletion (S1 File). The most abundant trichothecene produced by wild-type strains of this fungal species is harzianum A, which consists of EPT with an eight-carbon side chain (octatrienedioate) esterified to a hydroxyl group at C4 (Fig 2). Harzianum A does not have an ester or hydroxyl group at C15. Analysis of *T*. *arundinaceum tri3* deletion mutants indicated that harzianum A production was reduced by 92–94% compared to the wild-type progenitor strain (Fig 4A, 4B and 4D). However, GC-MS analysis revealed that the *tri3* mutants produced relatively high levels of trichodermol (4-hydroxy EPT) and two other harzianum A precursors, EPT and its immediate precursor isotrichodiol (Fig 4G). In contrast, the wild-type progenitor strain produced only low levels of trichodermol (Fig 4F). Complementation of a *tri3* mutant with a wild-type copy of *TRI3* restored high levels of harzianum A production (Fig 4C and 4E). The trichothecene production phenotypes of the *tri3* mutants and complemented mutant indicate that the *T*. *arundinaceum* Tri3 catalyzes esterification of octatrienedioate to the C4 hydroxyl group of 4-hydroxy EPT. Thus, the results also indicate that different homologs of Tri3 can have different functions: C4 esterification in *T*. *arundinaceum* and C15 esterification in *Fusarium*. Given that *Microcyclospora tardicrescens*, *S*. *roseum* and *T*. *roseum* can produce trichothecenes esterified at C4 but not C15, we propose that *TRI3* functions in C4 esterification in these fungi as well.

#### *Trichoderma arundinaceum* and *Myrothecium roridum TRI17*

**Fig 5:**
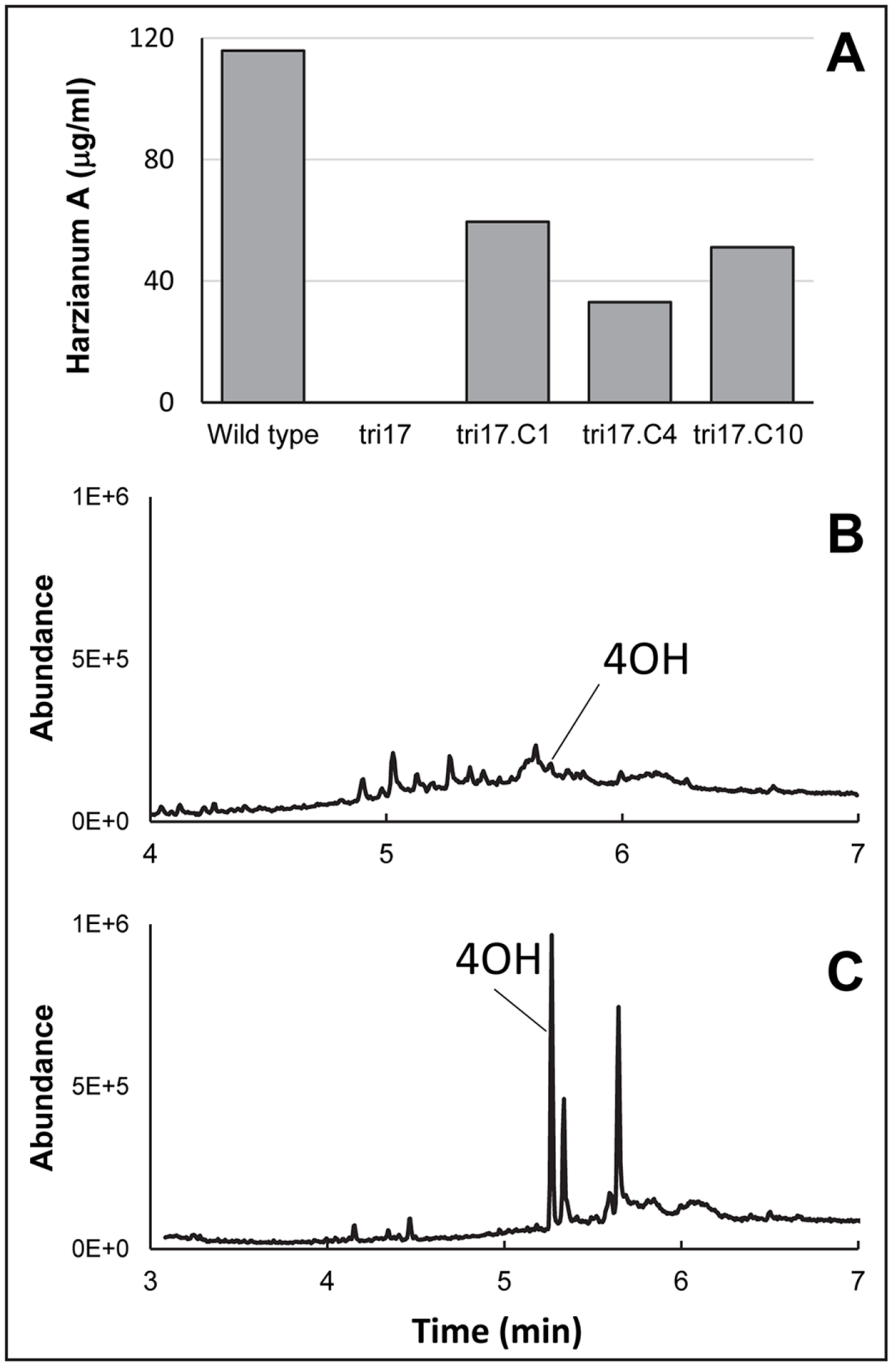
Functional analysis of *TRI17* in *Trichoderma arundinaceum*. (**A**) Quantitation of harzianum A production in the wild-type strain Ta37, *tri17* mutant strain tri17.139, and three tri17.139-derived strains (tri17.C1, tri17.C4 and tri17.C10) that were complemented with a wild-type copy of *T*. *arundinaceum TRI17*. (**B** and **C**) Total ion chromatograms of culture extracts of (**B**) wild-type progenitor strain Ta37 and (**C**) *tri17* deletion mutant tri17.139. The trichothecene biosynthetic intermediate trichodermol (4-hydroxy EPT) is indicated at 5.268 min. 4OH indicates trichodermol (4-hydroxy EPT).

The octatrienedioate substituent in harzianum A is thought to be derived from a polyketide [6] and, therefore, likely requires a PKS gene for its synthesis. We detected homologs of the PKS gene *TRI17* in *Myrothecium*, *Spicellum*, *Trichoderma*, and *Trichothecium* in addition to *Stachybotrys*, the fungus in which the gene was originally reported [21]. Its presence in the *Stachybotrys TRI* cluster led Semeiks et al. [21] to propose that Tri17 catalyzes synthesis of the polyketide portion of the macrolide ring of macrocyclic trichothecenes, but they did not confirm its role in trichothecene biosynthesis. Therefore, we used gene deletion and complementation analyses to determine whether *TRI17* is required for synthesis of the polyketide side chain of harzianum A in *T*. *arundinaceum* (S1 File). If *TRI17* is required for synthesis of the polyketide, deletion of the gene should block formation of harzianum A. *tri17* deletion mutants did not produce detectable amounts of harzianum A, but did produce trichodermol (4-hydroxy EPT), and complementation of a mutant with a wild-type copy of *TRI17* restored harzianum A production (Fig 5A, 5B and 5C). These results demonstrate that *TRI17* is required for synthesis of harzianum A and are consistent with the hypothesis that Tri17 catalyzes synthesis of the polyketide precursor of octatrienedioate.

Because the 6,7-dihydroxy-2,4-octadienoate substituent that occurs in the macrolide ring of some macrocyclic trichothecenes is similar in structure to octatrienedioate (Fig 2), it is possible that the *TRI17* homolog from a macrocyclic trichothecene-producing fungus could complement the *T*. *arundinaceum tri17* mutant. To test this hypothesis, we introduced a wild-type copy of *M*. *roridum TRI17* into a *T*. *arundinaceum tri17* mutant (S1 File). Liquid chromatography-tandem mass spectrometry (LC-MS/MS) analysis revealed that harzianum A production was restored in the resulting transformants and, thus, that *M*. *roridum TRI17* can complement the *T*. *arundinaceum tri17* mutant (S5 Fig). This finding indicates that biosynthesis of the polyketide-derived substituents of harzianum A and some macrocyclic trichothecenes (e.g., roridins and satratoxins) requires the same polyketide precursor.

#### *Beauveria bassiana TRI* genes

We attempted to induce trichothecene production in *B*. *bassiana* ARSEF 2860 and *C*. *confragosa* UM487 by growing each fungus under multiple conditions that induce trichothecene production in other fungi. However, trichothecenes were not detected in extracts from any of the resulting cultures. To obtain evidence as to whether the *B*. *bassiana TRI* cluster is functional, we heterologously expressed selected *TRI* genes from *B*. *bassiana* in either *Fusarium verticillioides* or *Saccharomyces cerevisiae*, two trichothecene-nonproducing fungi that have been used previously to determine *TRI* gene function [20,26,34]. We used *F*. *verticillioides* for analysis of *TRI4* and *TRI22*, which have introns, and *S*. *cerevisiae* for analysis of *TRI101*, which does not have introns. In the heterologous expression experiments, a *TRI* gene was introduced into *F*. *verticillioides* or *S*. *cerevisiae* by standard transformation methods [26]; selected trichothecene biosynthetic intermediates were added to cultures of the resulting transformants; and the ability of the cultures to modify the intermediates was assessed by GC-MS.

**Fig 6:**
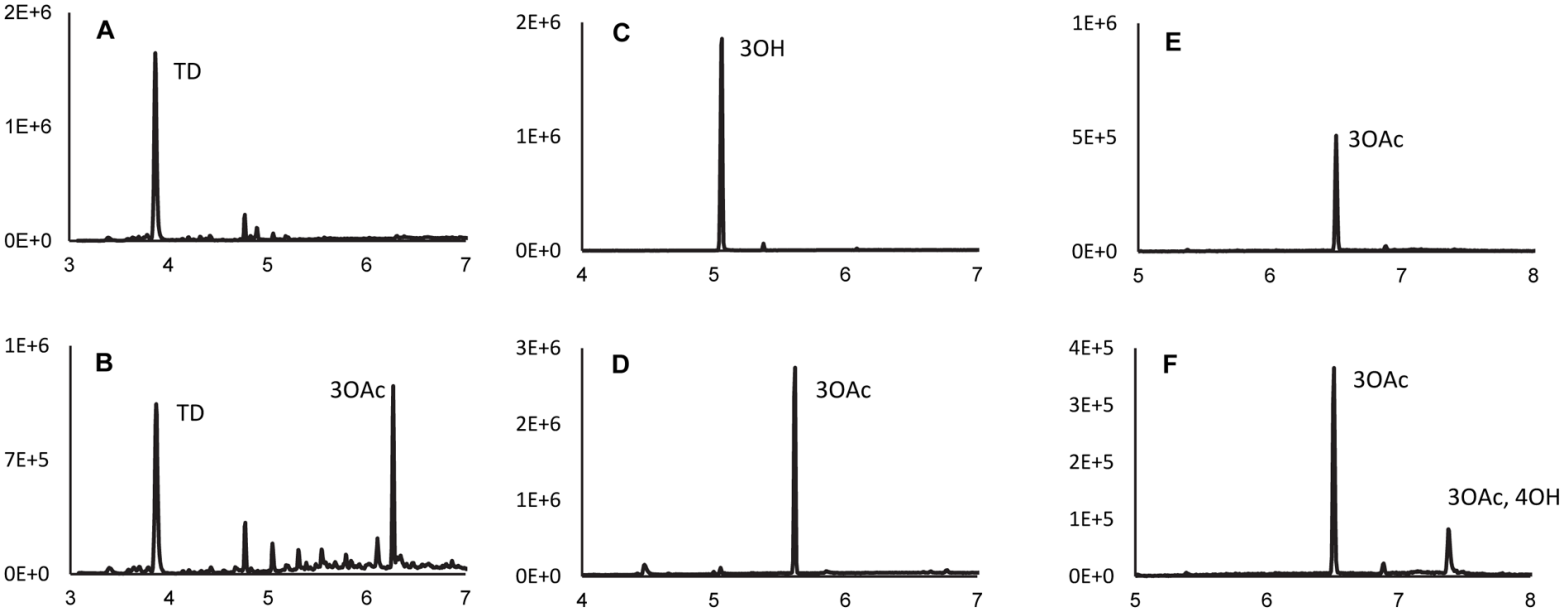
Total ion chromatograms from GC-MS analysis of cultures from heterologous expression of *TRI* genes from *Beauveria bassiana* strain ARSEF 2860. (**A)** wild-type *Fusarium verticillioides* grown on YEPD medium containing trichodiene (TD); (**B**) *F*. *verticillioides* expressing the *B*. *bassiana TRI4* grown on YEPD medium containing trichodiene; (**C**) *ayt1* mutant of *Saccharomyces cerevisiae* grown on YG medium containing isotrichodermol (3-hydroxy EPT, 3OH); (**D**) *ayt1* mutant of *S*. *cerevisiae* expressing the *B*. *bassiana TRI101* grown on YG medium containing isotrichodermol; (**E**) wild-type *F*. *verticillioides* grown on YEPD medium containing isotrichodermin (3-*O*-acetyl EPT, 3OAc); (**F**) *F*. *verticillioides* expressing the *B*. *bassiana TRI22* grown on YEPD medium containing isotrichodermin. In each chromatogram, the Y-axis is total ion abundance, and the X-axis is time in minutes. The peak labeled 3OAc,4OH indicates 4-hydroxy isotrichodermin (3-*O*-acetyl-4-hydroxy EPT).

In the first heterologous expression experiment, we introduced the *B*. *bassiana TRI4* gene into *F*. *verticillioides*. In trichothecene-producing fungi, Tri4 catalyzes oxygenation at three or four positions of trichodiene leading to the formation of EPT or 3-hydroxy EPT, respectively. Therefore, we reasoned that expression of the *B*. *bassiana TRI4* homolog in *F*. *verticillioides* would lead to the conversion of exogenously added trichodiene to EPT or 3-hydroxy EPT. Although *F*. *verticillioides* does not produce trichothecenes or have a *TRI* cluster, wild-type strains of the fungus can acetylate the C3 hydroxyl of trichothecenes and, as a result, can convert 3-hydroxy EPT to 3-*O*-acetyl EPT (isotrichodermin) [18]. Thus, if 3-hydroxy EPT were formed by expression of *B*. *bassiana TRI4* in *F*. *verticillioides*, it would be converted to 3-*O*-acetyl EPT by the endogenous C3 acetylase activity. Indeed, addition of trichodiene to cultures of *F*. *verticillioides* expressing the *B*. *bassiana TRI4* gene resulted in formation of 3-*O*-acetyl EPT (Fig 6A and 6B), thereby confirming that the *B*. *bassiana TRI4* is functional, and that it is required for oxygenation of trichodiene at four positions to yield 3-hydroxy EPT, the same function reported in *Fusarium* [18,20].

In a second experiment, we introduced the *B*. *bassiana TRI101* gene into *S*. *cerevisiae*. In trichothecene-producing fusaria, Tri101 catalyzes trichothecene C3 acetylation. The *S*. *cerevisiae* Ayt1 enzyme also catalyzes trichothecene C-3 acetylation. Therefore, in order to study the activity of *B*. *bassiana* Tri101 in this yeast species, we expressed *TRI101* in an *ayt1* deletion mutant. Addition of 3-hydroxy EPT (isotrichodermol) to cultures of a *S*. *cerevisiae ayt1* mutant expressing the *B*. *bassiana TRI101* homolog resulted in the formation of 3-*O*-acetyl EPT (isotrichodermin), thereby confirming that *B*. *bassiana TRI101* confers trichothecene C3 acetylation (Fig 6C and 6D).

In a third experiment, we introduced the *B*. *bassiana TRI22* homolog into *F*. *verticillioides*, and assessed whether expression of the gene conferred trichothecene C4 hydroxylation, the reaction catalyzed by Tri22 in *T*. *arundinaceum* [17]. Addition of 3-*O*-acetyl EPT to cultures of *F*. *verticillioides* expressing *B*. *bassiana TRI22* resulted in formation of a novel trichothecene product that was isolated and identified as 3-*O*-acetyl-4-hydroxy EPT on the basis of mass spectral and NMR data (Fig 6E and 6F, S6 Fig). This result indicated that *B*. *bassiana* Tri22 catalyzes trichothecene C4 hydroxylation. Together, the heterologous expression experiments described above indicate that the *B*. *bassiana TRI* genes examined are functional. Given this, the rest of the *B*. *bassiana TRI* genes might also be functional. If this is the case, our inability to induce trichothecene production in cultures of *B*. *bassiana* in the current study was likely because we did not use suitable culture conditions.

### Phylogenetic analysis of *TRI* genes

To gain insight into the variation of *TRI* gene homologs, we generated phylogenetic trees for individual *TRI* genes and for concatenated sequences of three of the genes. In preliminary analyses of individual *TRI* genes, we employed outgroup sequences of non-*TRI* genes from fungi that do not produce trichothecene and do not have a *TRI* cluster. Although distantly related, these outgroup sequences aligned to *TRI* sequences. In trees inferred from the resulting alignments, *Microcyclospora* homologs were consistently either the most or among the most basal lineages of *TRI* genes (S7 Fig). Given this and its distant relationships to the other trichothecene-producing fungi examined in this study, *Microcyclospora* homologs were used as the root in subsequent *TRI* gene trees that excluded a non-*TRI*-gene outgroup.

**Fig 7:**
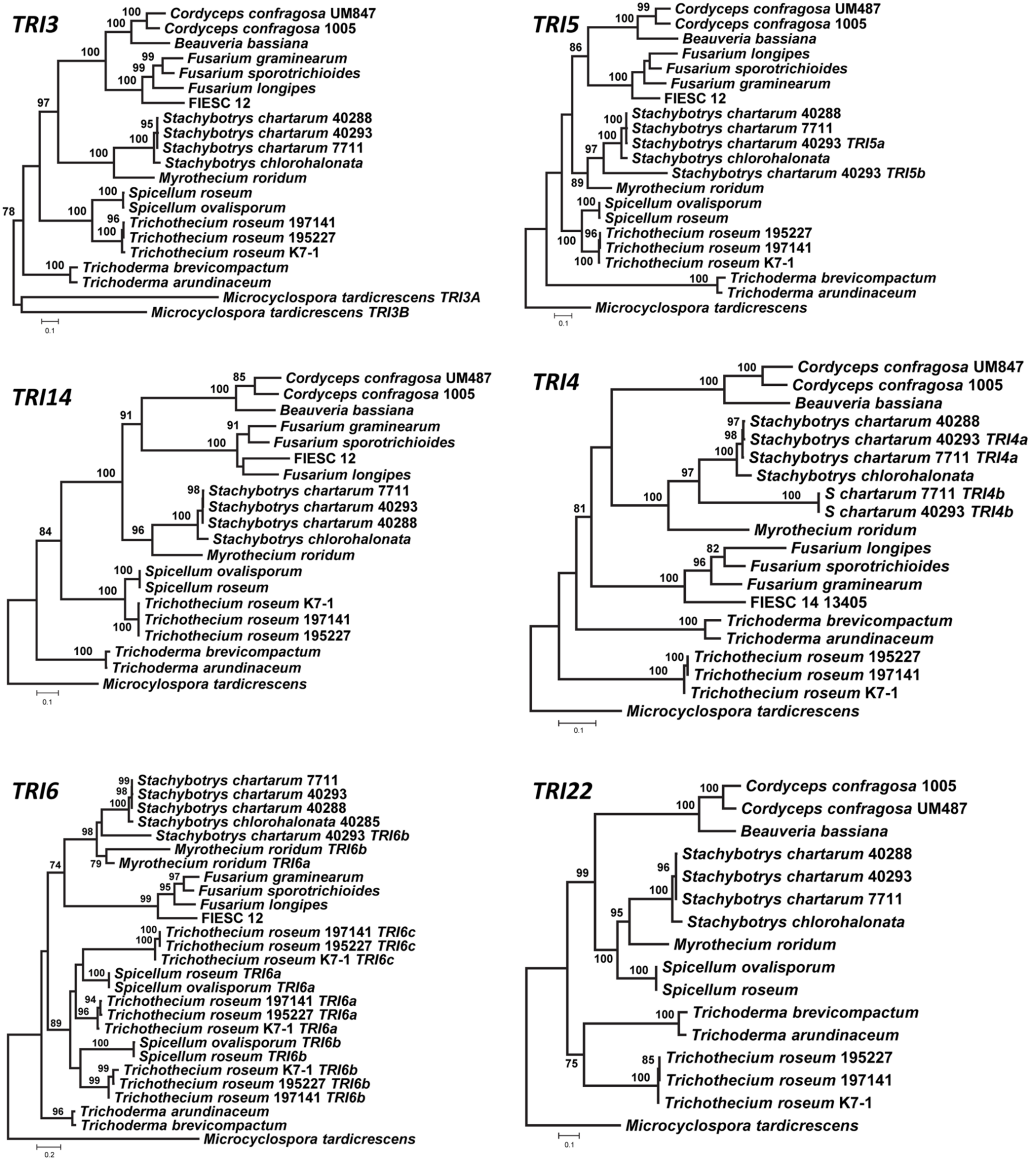
Maximum likelihood trees inferred from sequences of selected *TRI* genes: *TRI3*, *TRI4*, *TRI5*, *TRI6*, *TRI14*, and *TRI22*. Trees for *TRI10*, *TRI12* and *TRI18* are shown in the Supporting Information (S8 Fig). Numbers near branch nodes are bootstrap values based on 1000 pseudoreplicates.

**Fig 8:**
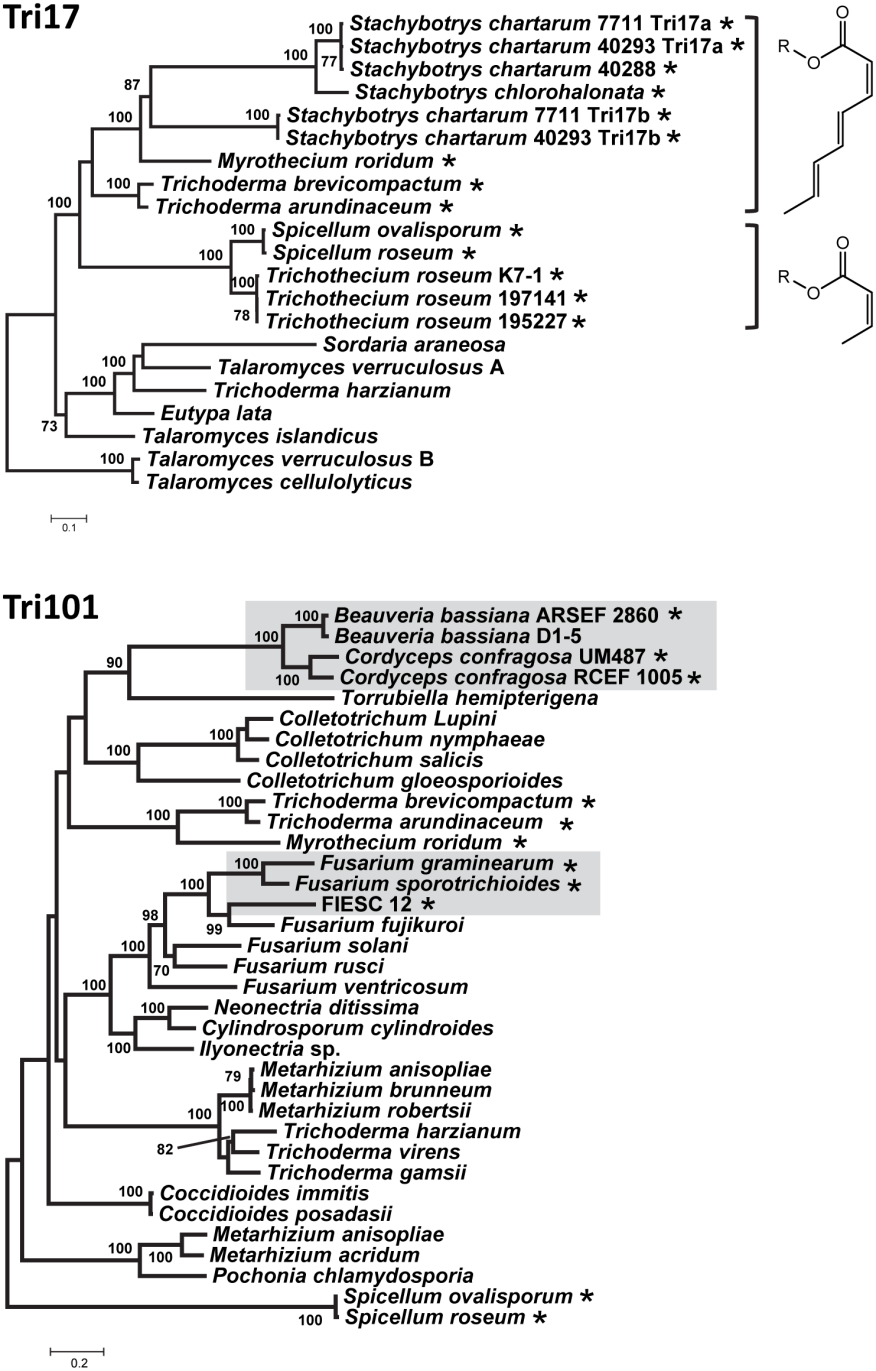
Maximum likelihood trees inferred from predicted amino acid sequences of Tri17 (top) and Tri101 (bottom) and related homologs from trichothecene-nonproducing fungi. The chemical structures shown to the right of the Tri17 tree are predicted structures of polyketides synthesized by the different Tri17 homologs. Asterisks (*) indicate species/strains that produce trichothecenes or are predicted to produce trichothecene based the presence of *TRI* genes. In the *TRI101* tree, the gray boxes indicate the strains/species that have a *TRI101* gene that functions or is likely to function in trichothecene biosynthesis. Numbers near branch nodes are bootstrap values based on 1000 pseudoreplicates. Strain designations are shown only for species with two or three strains included in a tree.

In trees inferred from homologs of individual *TRI* genes, relationships among more closely related homologs were generally well resolved (bootstrap values >70%), whereas relationships among more distantly related homologs were generally not well resolved (Figs 7 and 8, S8 Fig). In most single-*TRI*-gene trees, *Myrothecium* and *Stachybotrys* formed a well-supported clade, and with the exception of *TRI22*, *Spicellum* and *Trichothecium* formed a well-supported clade. In addition, *Beauveria*, *Cordyceps* and *Fusarium* also formed a well-supported clade in which *Beauveria* and *Cordyceps* had a sister relationship. Although branch conflicts were observed in comparisons of some *TRI* gene trees, most of the conflicts were not statistically supported by bootstrap analysis. We also performed Shimodaira-Hasegawa (SH) [35] and the Approximately Unbiased (AU) [36] tests to assess the significance of conflicting branches with bootstrap values > 70. According to the results of these tests, the conflicts were not significant, with one exception; in the *TRI22* tree, *Spicellum* homologs grouped in a well-supported clade with *Myrothecium* and *Stachybotrys* rather than with *Trichothecium* (Fig 7). This result suggests that the evolutionary history of *TRI22* differs from other *TRI* genes in *Spicellum*, a phenomenon that has been previously reported for some *Fusarium TRI* genes [37,38].

We surmised that trees inferred from multiple *TRI* genes would better reflect relationships of *TRI*-cluster homologs than single-*TRI*-gene trees. Therefore, we inferred a phylogenetic tree from concatenated sequences of *TRI3*, *TRI5* and *TRI14*, the only three *TRI* genes that were common to all fungi that were the focus of this study (Table 3). Trees inferred from these three genes individually did not have any well-supported branches that conflicted and were not significantly different from one another according to the SH and AU tests. Although results of a partition homogeneity test indicated inclusion of *F*. *graminearum* sequences resulted in significant heterogeneity in the data, relationships between genera did not differ in the concatenated gene trees with or without inclusion of *F*. *graminearum* sequences. Therefore, *F*. *graminearum* sequences were included in the final concatenated dataset.

**Fig 9:**
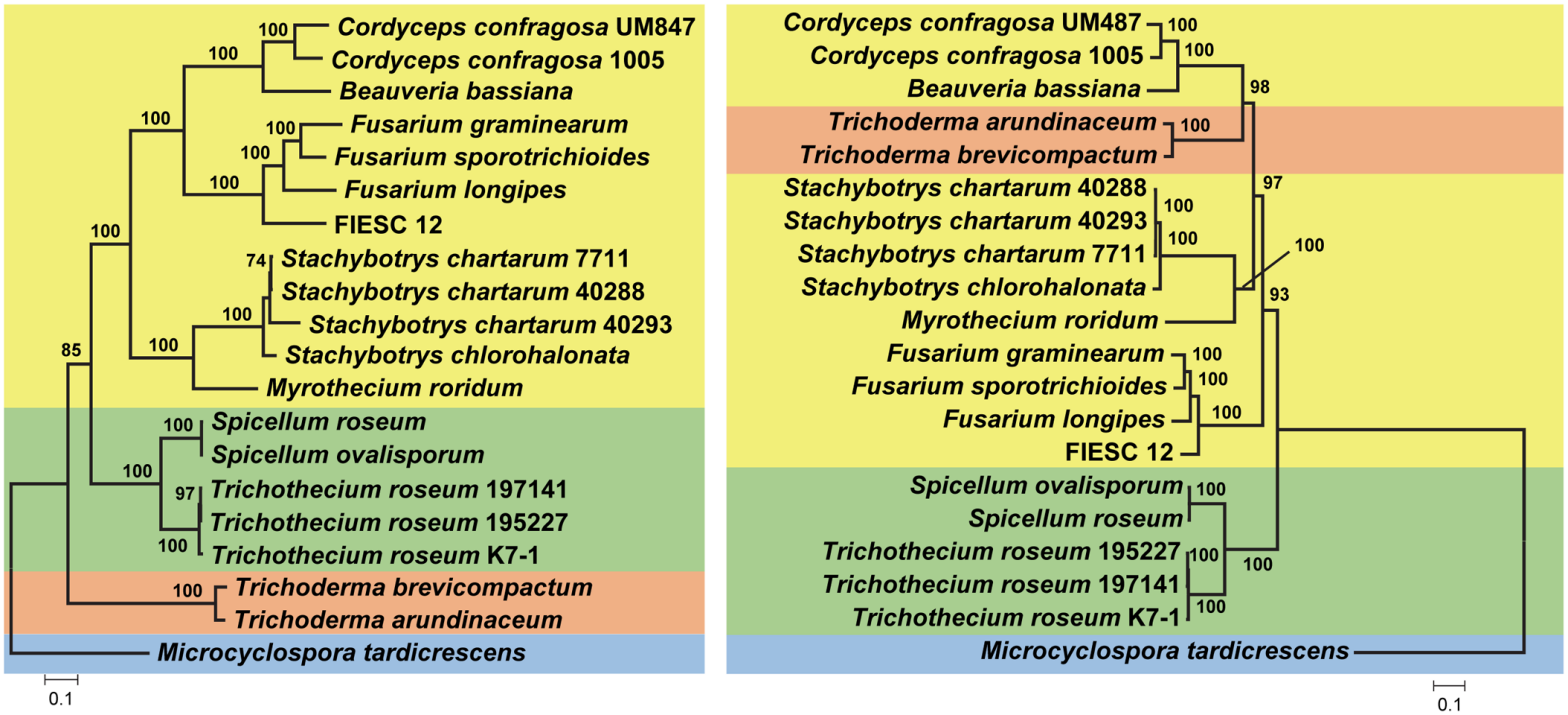
Comparison phylogenetic tree inferred from concatenated sequences of *TRI3*, *TRI5* and *TRI14* (left) and a species phylogeny inferred from concatenated sequences of 20 housekeeping genes (right). Numbers near branch nodes are bootstrap values from 1000 pseudoreplicates. Only bootstrap values greater than 70% are shown. The housekeeping genes used in this analysis are listed in S3 File.

Some relationships among *TRI* gene homologs that were evident in single-*TRI*-gene trees were also evident in the concatenated gene tree. For example, *Myrothecium* and *Stachy*botrys formed a well-supported clade, as did *Beauveria*, *Cordyceps*, and *Fusarium* in the concatenated and most single-*TRI*-gene trees (Figs 7 and 9). The concatenated-*TRI*-gene tree had high bootstrap support for almost all branches, and therefore provided information for relationships of more distantly related *TRI* clusters. Based on the clades resolved in the concatenated-gene tree, we divided the cluster homologs into four lineages: lineage A consisted of the outgroup, *M*. *tardicrescens*; lineage B was the next most basal clade and consisted of *Trichoderma* sequences; lineage C consisted of *Spicellum* and *Trichothecium* sequences; and lineage D consisted of *Beauveria*, *Cordyceps*, *Fusarium*, *Myrothecium* and *Stachybotrys* sequences (Figs 3 and 9). Although there were no consistent differences in gene content of the different cluster lineages, lineages A-C occurred in fungi that produce less complex trichothecenes (i.e., with a hydroxyl or ester at C4 of EPT, a carbonyl at C8 in some cases, and a hydroxyl at C7 in one case), whereas lineage D cluster homologs occurred in fungi that can produce more complex trichothecenes (i.e., with carbonyl, hydroxyl or ester groups at up to five positions of EPT) (Fig 2) [7].

Visual inspection indicated that there was one or more well-supported branches (bootstrap value > 70) in the *TRI10*, *TRI18*, *TRI22* and *TRI101* trees that conflicted with branches in the concatenated-*TRI3-5-14*-gene tree. Results of SH and AU tests indicated that the conflicts for *TRI22* and *TRI101* were significant, but those for *TRI10* and *TRI18* were not (S2 File).

To compare phylogenetic relationships of *TRI* cluster homologs to the relationships of the fungi in which the homologs occur, we inferred a species tree from the concatenated sequences of 20 housekeeping genes. We analyzed trees inferred from each housekeeping gene individually and assessed whether conflicts between the single-gene trees affected the species tree inferred from all 20 genes. The results of these analyses are shown in S3 File and suggest that the 20-housekeeping-gene tree provides a reasonable estimate of the species phylogeny. The high bootstrap values for almost all branches in the species tree provided evidence for the hierarchical relationships of most of the genera examined (Fig 9). There were two notable conflicts in the topologies of the species tree and the concatenated-*TRI*-gene tree. First, the sister relationship of *Beauveria-Cordyceps* and *Fusarium* observed in the *TRI* tree did not exist in the housekeeping gene tree; and second, the sister relationship of *Beauveria*-*Cordyceps* and *Trichoderma* observed in the housekeeping gene tree did not exist in the *TRI* tree. We used SH and AU tests to assess the significance of the conflicts between the trees overall and between the branches noted above. In a first set of tests, the *TRI* tree was constrained to conform to the housekeeping-gene tree, and housekeeping-gene tree were constrained to conform to the *TRI* tree. In these reciprocal assessments, the constrained trees were significantly worse than the unconstrained trees (p < 0.05). In a second set of tests, the *TRI* tree was constrained to include a sister relationship of *Beauveria*-*Cordyceps* and *Trichoderma*, and the housekeeping-gene tree was constrained to include the sister relationship of *Beauveria*-*Cordyceps* and *Fusarium*. Both tests indicated that the conflicts were significant (p < 0.05). In addition, none of the trees inferred from individual housekeeping genes included a well-supported *Beauveria*-*Cordyceps*-*Fusarium* clade (S3 File), and none of the single-*TRI*-gene trees included a well-supported *Beauveria*-*Cordyceps*-*Trichoderma* clade.

Homologs of *TRI101* have been identified in trichothecene-producing and nonproducing species of *Fusarium* and other fungal genera [39,40]. In the current study, BLAST analyses indicated that the *Beauveria* and *Cordyceps TRI101* homologs were more similar to *TRI101* homologs in some other genera of trichothecene-nonproducing fungi than they were to homologs in *Fusarium*. To further investigate sequence differences of *TRI101* homologs from *Beauveria*, *Cordyceps*, and *Fusarium*, we conducted a phylogenetic analysis with *TRI101* homologs from diverse Ascomycetes. The resulting tree suggests that *TRI101* homologs from *Beauveria* and *Cordyceps* are more closely related to a homolog from the trichothecene-nonproducing fungus *Torrubiella hemipterigena* than to homologs from trichothecene-producing fusaria (Fig 8). The tree also suggests that *TRI101* homologs in trichothecene-producing fusaria are more closely related to homologs in trichothecene-nonproducing species of *Fusarium*, *Cylindrosporum*, *Ilyonectria*, and *Neonectria* than they are to the homologs in *Beauveria*-*Cordyceps*. The relatively distant relationships of *TRI101* homologs in *Beauveria*-*Cordyceps* and trichothecene-producing species of *Fusarium* were unexpected given the close relationships of other *TRI* genes in these fungi.

## Discussion

In this study, a combination of genomic, phylogenetic, functional, and biochemical analyses has provided unprecedented insights into the evolutionary history of trichothecene biosynthesis in representatives of diverse genera of filamentous fungi. Our results indicate that structural diversity of trichothecenes produced by these fungi has arisen largely from gain, loss and changes in function of *TRI* genes during the collective evolutionary histories of *TRI* loci. Further, our phylogenetic analyses indicate that the evolutionary histories of *TRI* genes do not necessarily mirror the phylogenetic relationships of trichothecene-producing fungi, a phenomenon that has been observed for multiple fungal SM biosynthetic genes [24,37,38,41,42]. Below, we discuss the evidence for gain, loss and functional changes of *TRI* genes and consider the relationships between trichothecene structural changes and divergence of *TRI* cluster homologs.

### *TRI*-gene gain

We consider gain of a *TRI* gene to be the addition of a gene to trichothecene biosynthesis that was not previously involved in the process. Gain is suggested by the absence of a *TRI* gene in the genomes of multiple trichothecene-producing fungi, particularly those with a basal *TRI* cluster (lineage A and B clusters), and the presence of the gene in the genome(s) of only one or a few fungi. *TRI1*, *TRI7*, *TRI8*, *TRI11*, and *TRI13* are examples of such genes, because they were absent in all the fungi examined except *Fusarium*. Multiple mechanisms, including neofunctionalization, horizontal gene transfer (HGT), and horizontal chromosome transfer, have the potential to contribute to gain of a SM biosynthetic gene in fungi. The presence of *TRI* gene paralogs in several fungi suggested gain of some *TRI* genes might have resulted from neofunctionalization (i.e., the process of gene duplication and subsequent divergence in function of a resulting paralog). However, with the exception of the paralogs, known *TRI* genes are more closely related to non-*TRI* genes than they are to other *TRI* genes [21,24,37]. This suggests that neofunctionalization of *TRI* genes has not contributed to *TRI*-gene gain, but instead neofunctionalization of closely related non-*TRI* genes has contributed to gain.

*TRI*-gene gain may have also resulted when non-*TRI* genes changed function due to selection or other evolutionary processes to become involved in trichothecene biosynthesis. In fungi, SM biosynthetic gene clusters can degenerate such that some genes are pseudogenized or deleted and others remain intact [42–45]. If a *TRI* gene were gained by adaptation of a gene once involved in another process, the gene could have originated in a degenerating cluster. In our search for outgroups for phylogenetic analyses of individual *TRI* genes, BLASTx analysis of the fungal protein database in GenBank indicated that distantly related homologs of *TRI* genes occur in other fungi (Fig 8, S7 Fig). Furthermore, *F*. *graminearum* and *F*. *sporotrichioides* have genes that can partially compensate for the absence of *TRI* genes in deletion mutants [46,47]. Such genes encode enzymes that can modify trichothecene structures, and suggest another possible origin of gained *TRI* genes.

The discussion above indicates that multiple mechanisms could have contributed to gain of *TRI* genes, but that the mechanisms responsible for gain of specific genes are not evident from our analyses. The trichothecene C3 acetylation gene, *TRI101*, is a possible exception. Homologs of *TRI101* are present in some trichothecene-producing fungi and in many trichothecene-nonproducing fungi [39,48]. In fact, all trichothecene-nonproducing species of *Fusarium* that have been examined have a *TRI101* homolog, which is often designated as *TRI201* [49]. All of the fungi examined here with a lineage-A–C *TRI* cluster and some fungi with a lineage-D cluster (i.e., *Myrothecium* and *Stachybotrys*) produce trichothecenes that lack a C3 acetyl group, and therefore do not require *TRI101* for trichothecene biosynthesis. But, some trichothecene-producing fungi that do not require *TRI101* for production have a *TRI101* homolog (Fig 8) that is not located near other *TRI* genes. The presence of *TRI101* in some trichothecene-nonproducing fungi and trichothecene-producing fungi that do not require C3-acetylation activity indicates that some *TRI101* homologs have a function(s) other than trichothecene biosynthesis. In most trichothecene-producing fusaria that have been examined, *TRI101* is not in the *TRI* cluster, but instead is located in the same genomic context as the homolog in some trichothecene-nonproducing species [40,49]. In addition, there is evidence that *TRI101* has translocated into the *TRI* cluster rather than out of it during the evolutionary history of the *Fusarium incarnatum*-*equiseti* species complex (FIESC) [24]. These observations plus the knowledge that *TRI101* functions in trichothecene C3 acetylation in both *Beauveria* and *Fusarium* suggest that *TRI101* has become incorporated into trichothecene biosynthesis (i.e., gained) in *Beauveria*-*Cordyceps* and *Fusarium*. The presence of *TRI101* in trichothecene-nonproducing fungi further suggests that *TRI101* gain has involved its adaptation from another function. It has been proposed that in *Fusarium TRI101* and *TRI201* are paralogs [49]. If this is the case, gain of *TRI101* could be a result of neofunctionalization, whereby the ancestral gene was and the *TRI201* paralog is involved in a process other than trichothecene biosynthesis, and the *TRI101* paralog diverged to function in trichothecene biosynthesis.

**Fig 10:**
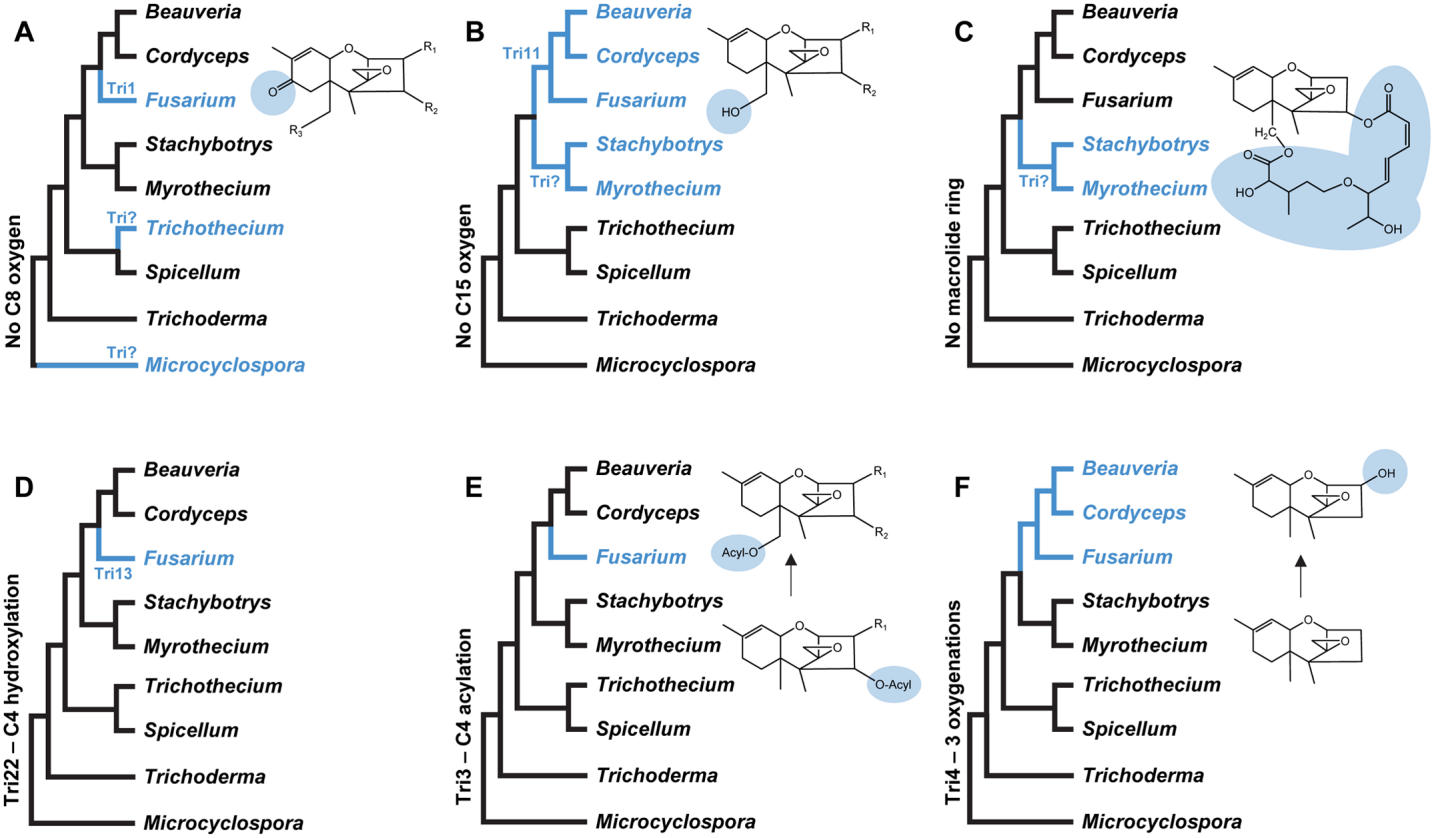
Scenarios for changes in trichothecene biosynthesis during evolutionary divergence of *TRI* cluster homologs. The scenarios presume the ancestral *TRI* cluster and trichothecene pathway presented in Fig 12. In each scenario, the tree is a simplified version of the tree inferred from concatenated sequences of *TRI3*, *TRI5*, and *TRI14* (Fig 3). A change in biosynthesis from the ancestral state is indicated by a change in color of a branch from black to blue. **A.** acquisition of C8 oxygen by gain of Tri1 in *Fusarium* and an unknown enzyme(s) in *Microcyclospora* and *Trichothecium*; **B.** acquisition of C15 oxygen by gain of Tri11 in *Fusarium*, *Beauveria* and *Cordyceps*, and an unknown enzyme in *Myrothecium* and *Stachybotrys*; **C.** acquisition of macrolide ring by gain of unknown enzymes in *Myrothecium* and *Stachybotrys*; **D.** change in C4 hydroxylation enzyme from Tri22 to Tri13 during divergence of *Fusarium TRI* cluster; **E.** change in Tri3 function from C4 acylation to C15 acetylation during divergence of *Fusarium TRI* cluster; and **F.** change in Tri4 function from 3 to 4 oxygenations during divergence of *TRI* cluster lineage in *Fusarium*, *Beauveria*, and *Cordyceps*.

Results of the phylogenetic analysis of Tri101 homologs from trichothecene-producing and nonproducing fungi (Fig 8) suggests that the gain of *TRI101* and, therefore, the evolution of the C3 acetylation in the trichothecene biosynthetic pathway occurred independently in *Fusarium* and in *Beauveria*-*Cordyceps*. Two other trichothecene structural modifications, C8 and C15 oxygenation, appear to have also evolved independently in different fungi. Some *Fusarium*, *Microcyclospora*, and *Trichothecium* trichothecenes have a C8 oxygen atom (Fig 2). Functional analyses of *F*. *graminearum* and *F*. *sporotrichioides* indicate that Tri1 catalyzes trichothecene C8 oxygenation in *Fusarium* [50–53]. The absence of *TRI1* in the *Microcyclospora* and *Trichothecium* genome sequences indicates that an enzyme other than Tri1 catalyzes C8 oxygenation in these fungi, which in turn indicates that C8 oxygenation in *Fusarium* evolved independently of its evolution in *Microcyclospora* and *Trichothecium*. (Fig 10A). It is not known whether C8 oxygenation arose independently in *Microcyclospora* and *Trichothecium*.

Among the fungi examined in this study, only *Fusarium*, *Myrothecium* and *Stachybotrys* are reported to produce trichothecenes with a C15 oxygen. In *Fusarium*, Tri11 catalyzes trichothecene C15 oxygenation [54]. The absence of a *TRI11* homolog in the *Myrothecium* and *Stachybotrys* genome sequences (Table 3) indicates that a gene other than *TRI11* is required for C15 oxygenation in these fungi, and therefore that trichothecene C15 oxygenation in *Myrothecium* and *Stachybotrys* arose independently of its evolution in *Fusarium* (Fig 10B). On the other hand, the presence of *TRI11* homologs in *Beauveria*, *Cordyceps* and *Fusarium* suggests that gain of *TRI11* occurred prior to divergence of the *TRI* cluster homologs in these fungi (Fig 10B). To our knowledge and with the exception of *TRI17*, genes required for synthesis of the macrolide rings of macrocyclic trichothecene have yet to be identified. Production of macrocyclic trichothecenes only by fungi with lineage-D *TRI* clusters suggests that the formation macrolide rings of these trichothecenes resulted from gain of genes in the *Myrothecium*-*Stachybotrys* lineage of trichothecene-producing fungi (Fig 10C).

### *TRI*-gene loss

We consider that loss of a *TRI* gene results from pseudogenization or complete deletion of the gene such that a functional version of it is no longer present in a genome. Evidence for loss is the occurrence of a gene in multiple fungi with a more basal (i.e., lineage A and B) *TRI* cluster, but absence of the gene in one or more other fungi. *TRI4*, *TRI6*, *TRI10*, *TRI12*, *TRI13*, *TRI17*, and *TRI22* are examples of genes that have likely been lost (Table 3). Gene loss is reported to contribute to structural variation of multiple fungal SMs [42,55,56]. Given the structural diversity of trichothecenes, *TRI* gene loss was expected to contribute to variation in gene content among the fungi examined, and indeed was previously reported from analyses of *Fusarium* and *Stachybotrys* [21,50,55]. However, absence of *TRI4* in the *Spicellum* genomes was unexpected, because Tri4 catalyzes multiple reactions that are essential for formation of the EPT structure common to all trichothecenes (Fig 2) [18–20]. Production of trichothecenes by *S*. *roseum* 209012 (S4 Fig) indicates that the fungus has a gene(s) that compensates for the absence of *TRI4*. Furthermore, production of low levels of trichothecenes by *T*. *arundinaceum tri4* deletion mutants indicates the existence of a gene that can partially compensate for the absence of *TRI4* in this fungus [14]. The absence of *TRI4* in *Spicellum* strains raises a question: what caused the loss of an enzyme that catalyzes multiple reactions essential for trichothecene biosynthesis and its replacement with another enzyme?

The absence of *TRI6* and *TRI10* in the *B*. *bassiana* and *C*. *confragosa* genomes was also unexpected given that these genes regulate expression of *TRI* genes in *Fusarium* [57–59]. Assuming *B*. *bassiana* and *C*. *confragosa* produce trichothecenes under some conditions, the absence of *TRI6* and *TRI10* indicates the existence of two fundamentally different regulatory systems for trichothecene biosynthesis in fungi. Among the fungi examined, *B*. *bassiana* and *C*. *confragosa* are the only insect pathogens. This raises a question: does the apparent change in regulation of *TRI* gene expression in *B*. *bassiana* and *C*. *confragosa* reflect an adaptation of trichothecene production for a lifestyle that includes insect pathogenesis?

The absence of the MFS transporter gene *TRI12* was previously noted in analyses of the FIESC [24] and *Stachybotrys* species [21]. As a result, the absence of *TRI12* in the *B*. *bassiana* and *C*. *confragosa* genomes was not surprising, but instead contributes to evidence that *TRI12* is not essential for trichothecene production in fungi [60]. Presumably, another transporter(s) can compensate for the absence of Tri12 in trichothecene-producing fungi that lack *TRI12*. The presence of *TRI12* in all fungi with a lineages A–C *TRI* cluster and its absence in some fungi with a lineage-D cluster (Fig 3) suggests that *TRI12* was present in the ancestral *TRI* cluster. Further, the variable presence of *TRI12* in lineage-D *TRI* clusters suggests three independent losses of the gene: once in *Stachybotrys* after divergence from *Myrothecium*; once in the *Beauveria*-*Cordyceps* clade after divergence from *Fusarium*; and once in FIESC after divergence from other fusaria.

### *TRI13* and *TRI22*: An unusual case of gain and loss

Except for *F*. *graminearum*, all known trichothecene-producing fungi examined here can produce trichothecenes that have a hydroxyl or ester group at C4 (Fig 2). This suggests that C4 oxygenation arose early in the evolutionary history of trichothecene biosynthesis, and therefore, that the common ancestor of extant *TRI* clusters likely encoded an enzyme that catalyzed this reaction. The results of the current and previous studies indicate that C4 hydroxylation is catalyzed by Tri22 in *T*. *arundinaceum* [17] and *B*. *bassiana* (Fig 6E and 6F) but by Tri13 in *Fusarium* [55]. Together, the presence of *TRI22* in all the fungi examined here except *Fusarium* and the presence of *TRI13* only in *Fusarium* (Table 3) suggest that *TRI22* is the ancestral C4 hydroxylase gene, and that *TRI13* was acquired after the *Fusarium TRI* cluster diverged from the cluster in other genera (Fig 10D). With the exception of the position of *Spicellum* and absence of *Fusarium*, the topology of the *TRI22* tree is similar to the topology of the combined *TRI3-TRI5-TRI14* tree, suggesting that the evolutionary history of *TRI22* mirrors that of the *TRI* cluster to some extent. This, in turn, is consistent with the hypothesis that *TRI22* is the ancestral C4 hydroxylase gene. If this hypothesis is correct, *TRI22* would have been lost from and *TRI13* would have been gained during the evolutionary divergence of the *Fusarium* cluster. Within *Fusarium*, production of trichothecenes that lack a C4 oxygen (e.g., deoxynivalenol) results from pseudogenization of *TRI13* [55,61]. This observation suggests a possible scenario to explain how the gene conferring trichothecene C4 hydroxylation changed from *TRI22* to *TRI13*. The scenario is based on the idea that if some extant fusaria produce trichothecenes that lack a C4 oxygen, ancestral trichothecene-producing fusaria could have produced trichothecenes that lack a C4 oxygen as well. In the scenario, *TRI22* conferred C4 hydroxylation in ancestral trichothecene-producing fungi. Subsequently, during early divergence of the *Fusarium TRI* cluster, selection for production of trichothecenes with a C4 oxygen was relaxed and, as a result, *TRI22* was lost. This gave rise to production of trichothecenes that lack a C4 oxygen. Subsequent to *TRI22* loss, selection for production of trichothecene with a C4 oxygen was restored and, as a result, *TRI13* was gained. Because all trichothecene-producing fusaria that have been examined to date have a functional or pseudogenized *TRI13*, we surmise that gain of *TRI13* occurred early in divergence of the *Fusarium TRI* cluster [16,24,62]. After gain of *TRI13*, the gene was pseudogenized in one lineage of *Fusarium*, resulting in a mixed population in which some individuals produced trichothecenes that lacked C4 oxygen and others produced trichothecenes that have a C4 oxygen, a situation that still occurs in some lineages of *Fusarium* [37,63]. Thus, according to this scenario, trichothecene C4 hydroxylation has undergone a cycle whereby it existed in the ancestral trichothecene-producing fungus, was lost, then reacquired, and subsequently lost again.

### Changes in *TRI* gene function

The results of the current and previous studies indicate that some trichothecene structural diversity has resulted from changes in function of *TRI3* (Fig 4) and *TRI4* (Fig 6A and 6B) [18–20,25]. Tri3 catalyzes C4 acylation in *T*. *arundinaceum* and C15 acylation (acetylation) in *Fusarium*. We propose that the C4 acylation activity is ancestral and C15 acylation is derived, because trichothecenes produced by fungi with a lineage A or B *TRI* cluster (i.e., *Microcyclospora* and *Trichoderma*) have an acyl group at C4 but not at C15 (Fig 10E). The proposed ancestral and derived activities of Tri3 are consistent with the acquisition of C15 oxygenation in fungi with a lineage C and D *TRI* cluster (Fig 10B), because a hydroxyl group at C15 is a prerequisite for C15 acetylation catalyzed by the *Fusarium* Tri3 [33,64]. If the proposed ancestral and derived activities of Tri3 are correct, the low level of C4 acetylation activity reported for recombinant *F*. *graminearum* Tri3 [64] indicates that some of the ancestral activity has been retained in this fungus.

The results of this and previous studies indicate *Myrothecium* and *Trichoderma* Tri4 homologs catalyze oxygenation at three positions of trichodiene, whereas *Beauveria* (Fig 6A and 6B) and *Fusarium* Tri4 homologs catalyze oxygenation at four positions [18–20,25]. The three oxygenations result in formation of trichothecenes that lack a C3 hydroxyl, while the four oxygenations result in formation of trichothecenes that have a C3 hydroxyl. Further, trichothecenes that lack a C3 hydroxyl are produced by all the fungi with a lineage-A–C *TRI* cluster and some fungi with a lineage-D cluster, whereas trichothecenes that have a C3 hydroxyl are produced only by some fungi with a lineage-D cluster (i.e., *Fusarium* and presumably *Beauveria* and *Cordyceps*). Based on this information, we propose that the ability to catalyze three oxygenations is the ancestral condition of Tri4, and the ability to catalyze four reactions is derived (Fig 10F). This hypothesis is consistent with the idea that C3 acetylation is also a derived condition, because an oxygen atom at C3 is a prerequisite for C3 acetylation catalyzed by Tri101 [3,16]. There is evidence for changes in functions of other *TRI* genes/enzymes as well, because Tri1 and Tri8 are reported to differ in function within and/or among *Fusarium* species [26,50–52].

### Synthesis of polyketide-derived substituents of trichothecenes

**Fig 11:**
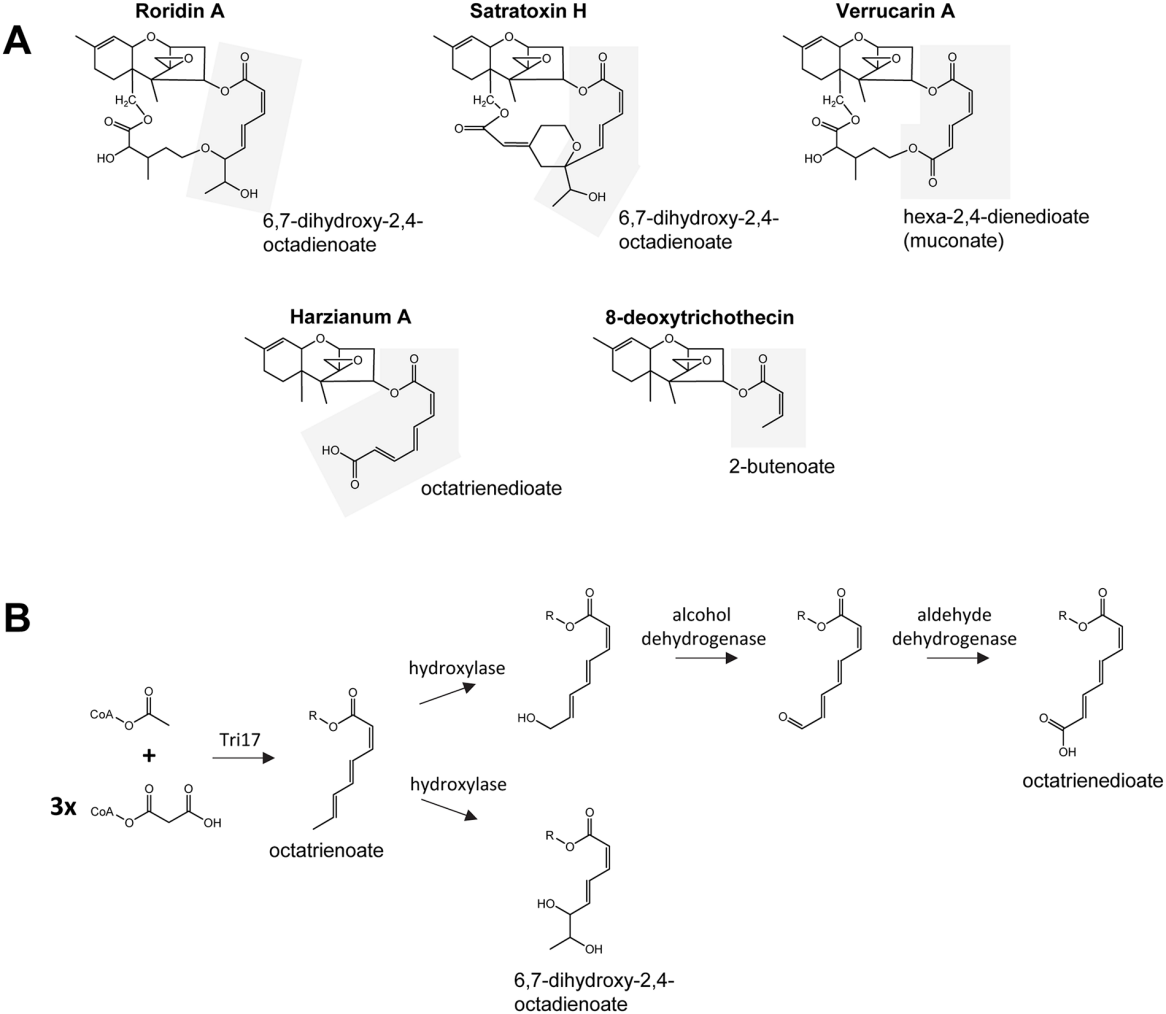
(**A**) Variation in the structure of polyketide-derived substituents in selected trichothecene analogs. The polyketide-derived substituent of each structure is highlighted with a gray background. Names below and/or to the right of highlighted areas are chemical or trivial names of the polyketide-derived substituents. Trichothecene names are indicated above each structure. (**B**) Proposed schemes for synthesis of octatrienedioate and 6,7-dihydroxy-2,4-octadienoate, the polyketide-derived substituents that occur in the structures of harzianum A and the macrocyclic trichothecene roridin A, respectively. In the proposed schemes, the parent compound of both substituents is 2,4,6-octatrienoate, an eight-carbon polyketide with three carbon-carbon double (enoyl) bonds. To form octatrienedioate, the polyketide would undergo a hydroxylation reaction followed by two oxidation reactions, first to form an aldehyde and second to form a carboxylic acid group. To form 6,7-dihydroxy-2,4-octadienoate, the polyketide would undergo two hydroxylation reactions during which an enoyl bond is lost. Formation of hexa-2,4-dienedioate, the polyketide-derived substituent in verrucarin A is not included in the figure, but would involve the same reactions as for synthesis of octatrienedioate, except that the parent polyketide would be a six-carbon polyketide with two enoyl bonds. It is not known whether the structural modifications of the polyketides occur before or after esterification to EPT. Thus, in the proposed schemes, R could be C4 of EPT, Co-enzyme A, or a hydrogen atom.

Functional analyses of the *T*. *arundinaceum* and *M*. *roridum TRI17* homologs indicate that Tri17 catalyzes synthesis of the polyketide precursors of the substituents esterified to C4 of some trichothecene analogs (Fig 5, S5 Fig). The polyketide-derived substituents of harzianum A and macrocyclic trichothecenes are made up of linear molecules that are either six (hexa-2,4-dienedioate) or eight (octatrienedioate and 6,7-dihydroxy-2,4-octadienoate) carbon atoms long (Fig 11A) [15,65]. The presence of *TRI17* in *Spicellum* and *Trichothecium* suggests that the Tri17 homologs in these fungi catalyze synthesis of the four-carbon chain (2-butenoyl) that is esterified to the C4 oxygen of trichothecenes produced by these fungi (Fig 11A). The variable lengths of the polyketide-derived substituents in trichothecenes produced by *Myrothecium*, *Stachybotrys*, *Spicellum*, *Trichoderma*, and *Trichothecium* suggest that collectively, Tri17 homologs can catalyze synthesis of four, six or eight-carbon polyketides. Furthermore, single species of *Myrothecium* and *Stachybotrys* and even single isolates of some species can produce macrocyclic trichothecenes that have six- or eight-carbon polyketide-derived substituents [66,67]. Thus, it is likely that in some species, a single Tri17 homolog can catalyze synthesis of both six and eight-carbon polyketides.

The Tri17 protein is predicted to include an enoyl reductase (ER) domain [21]. During polyketide biosynthesis, an ER domain catalyzes reduction of carbon-carbon double bonds to carbon-carbon single bonds [68]. PKSs that have the appropriate combination of other functional domains but lack a functional enoyl reductase domain catalyze synthesis of polyketides that have alternating double and single bonds. The polyketide-derived substituents in *Trichoderma*, *Myrothecium*, and *Stachybotrys* trichothecenes have such alternating double and single bonds. Thus, the Tri17 enoyl reductase domain is almost certainly nonfunctional.

In polyketide biosynthesis, carbon-chain length is controlled by the PKS enzyme [69]. In trichothecene biosynthesis, therefore, differences in polyketide-chain length (i.e., four carbons vs. six or eight carbons) likely result from differences in amino acid sequence of Tri17 homologs. In phylogenetic trees inferred from predicted amino acid sequences of Tri17 and related PKSs, *Spicellum* and *Trichothecium* Tri17 homologs, which likely catalyze synthesis of a four-carbon polyketide, form a clade basal to the *Myrothecium*, *Stachybotrys* and *Trichoderma* homologs, which likely catalyze synthesis of six- and eight-carbon polyketides (Fig 8). These phylogenetic relationships suggest that Tri17 homologs that catalyze synthesis of a four-carbon polyketide represent the ancestral Tri17 condition, whereas homologs that catalyze synthesis of six and/or eight-carbon polyketides represent a derived condition.

Given the predicted Tri17 functional domains and the structures of polyketide-derived substituents of harzianum A and macrocyclic trichothecenes, the polyketides precursors of these substituents are likely modified after release from Tri17. For example, the polyketide precursor of octatrienedioate is likely a linear, eight-carbon polyketide with alternating double and single carbon-carbon bonds, and one carboxylic acid group (Fig 11B). Because octatrienedioate has two carboxylic acid groups, its polyketide precursor likely undergoes modifications to form a second carboxylic acid group (Fig 11B). Likewise, the 6,7-dihydroxy-2,4-octadienoate substituent in some macrocyclic trichothecenes contains two adjacent hydroxyl groups. Because the polyketide precursor of this substituent is likely the same as the octatrienedioate precursor, formation of 6,7-dihydroxy-2,4-octadienoate would also require modifications of its polyketide precursor (Fig 11B).

### Inference of an ancestral trichothecene biosynthetic pathway

**Fig 12:**
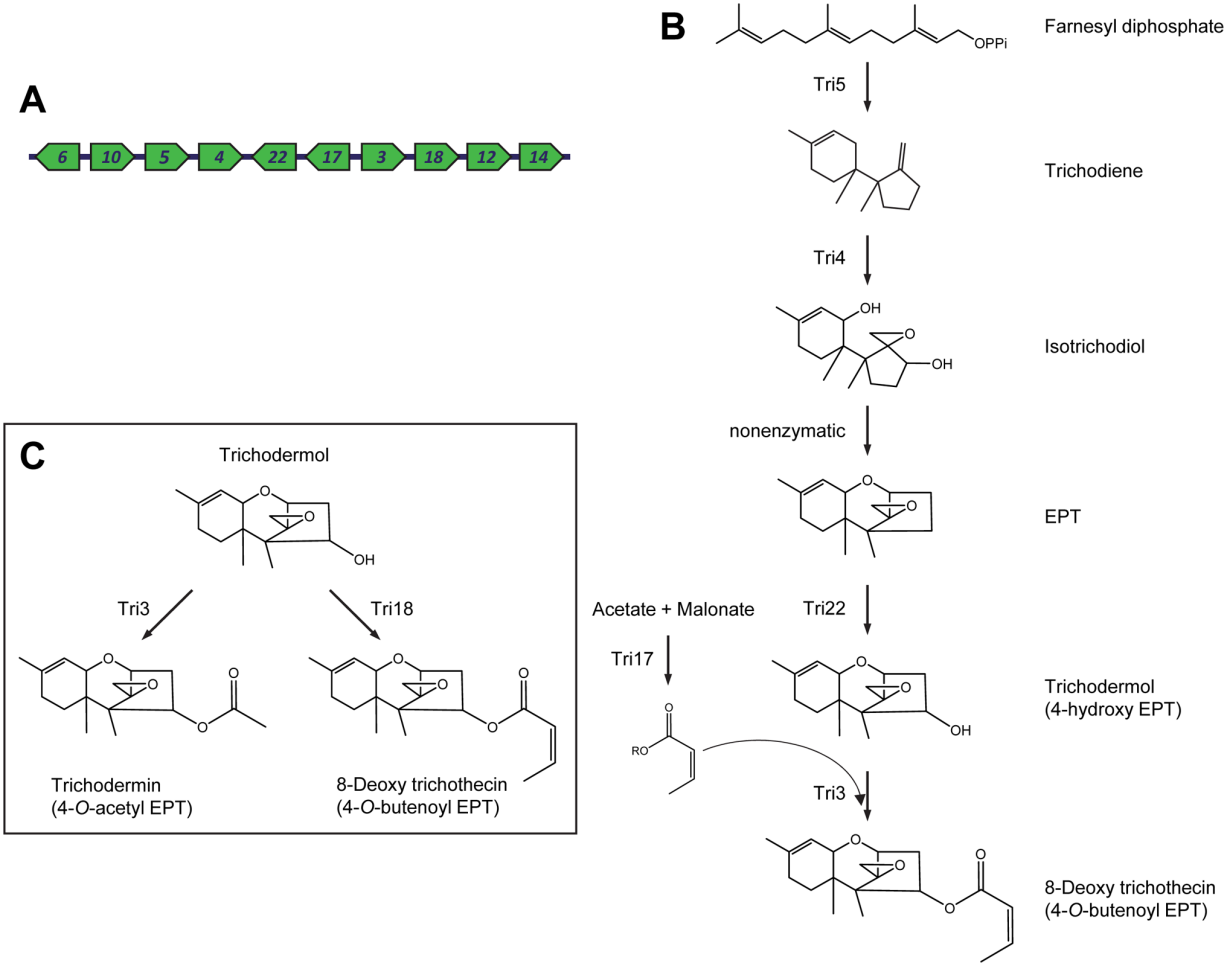
(**A**) Proposed ancestral *TRI* cluster based on the distribution of *TRI* genes among fungi examined in the current study. (**B**) Proposed ancestral trichothecene biosynthetic pathway based on inferred gene content of the ancestral *TRI* cluster and knowledge of *TRI* gene functions. (**C**) The presence of two acetyl/acyl transferase genes in the proposed ancestral cluster raises the possibility of a branch at the end of the ancestral pathway that would result in the formation of two trichothecene analogs: 4-acetyl EPT and 4-butenoyl EPT.

The structural diversity of trichothecene analogs produced by the fungi examined in this study combined with information on the distribution, phylogenetic relationships and functions of *TRI* genes allow for inference of ancestral states of the *TRI* cluster and trichothecene biosynthetic pathway (Fig 12). The inferred ancestral cluster included the structural genes *TRI3*, *TRI4*, *TRI5*, *TRI17*, *TRI18* and *TRI22*, the regulatory genes *TRI6* and *TRI10*, the transporter gene *TRI12*, and *TRI14* (Fig 12A). The presence of these 10 genes in the ancestral cluster is consistent with their presence in all fungi examined and/or their presence in all lineages of the *TRI* cluster (Fig 3). Based on observations discussed above, *TRI4* in this ancestral cluster conferred the ability to catalyze three oxygenation reactions to yield EPT [18,19], and *TRI3* conferred the ability to catalyze C4 rather than C15 acylation (Fig 12B). In addition to rationales described above, this role for *TRI3* in the ancestral cluster is consistent with the observation that during trichothecene biosynthesis in *T*. *arundinaceum*, Tri22 and Tri3 function in tandem; Tri22 catalyzes C4 hydroxylation, and Tri3 catalyzes acylation of the resulting C4 hydroxyl (Fig 4) [17]. This tandem function of Tri22 and Tri3 is also consistent with the presence of both *TRI22* and *TRI3* in fungi that have a C4 but not C15 ester. As noted above, the ancestral Tri17 likely catalyzed synthesis of a four-carbon polyketide. Thus, the product of the inferred ancestral pathway would be 8-deoxy trichothecin (4-*O*-butenoyl EPT) (Fig 12B).

We have included *TRI18* in the inferred ancestral cluster because of its widespread distribution among trichothecene-producing fungi. However, we do not know its function in these fungi nor its likely function in the ancestral trichothecene biosynthetic pathway. In all fungi examined, when *TRI17* is present, *TRI18* is located adjacent to or near it (Fig 3). This consistent physical linkage of *TRI17* and *TRI18* suggests that the two genes could function together in biosynthesis. Given that *TRI18* is predicted to encode an acyltransferase (Table 1), the most obvious possibility is that Tri18 catalyzes C4 esterification of the polyketide product of Tri17 to the C4 hydroxyl; i.e., Tri18 could have the same function as the proposed ancestral function of Tri3. Consistent with this idea is the evidence that there is a gene in *T*. *arundinaceum* that can partially compensate for *TRI3* in *tri3* deletion mutants of the fungus (Fig 4). Thus, one possibility is that in the ancestral trichothecene pathway, both Tri3 and Tri18 catalyzed esterification of the Tri17 product (butenoyl) to the hydroxyl group at C4. Another possibility is that both enzymes catalyzed trichothecene C4 esterification, but esterified different molecules to the C4 hydroxyl (e.g., acetyl and butenoyl). Such a difference in function of Tri3 and Tri18 would have caused a branch at the end of the ancestral pathway, with one branch leading to 8-deoxy trichothecin (4-*O*-butenoyl EPT) and the other leading to trichodermin (4-*O*-acetyl EPT) (Fig 12C). It is noteworthy that *S*. *roseum* can produce both of these metabolites (S4 Fig), and has homologs of both *TRI3* and *TRI18*. Thus, the trichothecene products of the ancestral *TRI* cluster could be the same as those produced by an extant species of *Spicellum*.

We have not included C8 oxygenation in the proposed ancestral pathway. However, the presence and absence of a C8 oxygenation step in the ancestral pathway are both consistent with currently available data. Our rational for not including a C8 oxygenation step in the pathway was based on: 1) the hypothesis that the ancestral pathway would have been simpler than most extant pathways; and 2) the trichothecene-C8-oxygenase gene(s) in *Microcyclospora* and *Trichothecium* has not been identified, and therefore its distribution is not known. Given this, it is not possible to say whether the gene(s) was likely to have been present in the ancestral *TRI* cluster. If the ancestral pathway included a C8 oxygenation step, this ability would have to have been lost four or more times and re-acquired at least once to account for the distribution of the C8-oxygenation ability among trichothecene-producing fungi examined in this study. On the other hand, if C8 oxygenation was absent in the ancestral pathway, three independent gain events could account for the current distribution of the ability to produce C8-oxygenated trichothecenes (Fig 10A). Identification of gene(s) required for C8 oxygenation in *Microcyclospora* and *Trichothecium* should provide insight into the whether the ancestral pathway included this reaction.

### Distribution and phylogenetic relationships of *TRI* genes

The sampling of fungi in the current study represents a majority of fungal genera in which trichothecene production has been reported [4–8]. The fungi are also phylogenetically diverse; *M*. *tardicrescens* is a member of the class Dothideomycetes, while the other fungi are members of five lineages within the class Sordariomycetes (order Hypocreales). Thus, the *TRI* cluster and trichothecene production are uncommon and discontinuously distributed among ascomycetous fungi. How the current distribution of the *TRI* cluster arose is not clear, but comparison of *TRI*-gene and species trees inferred in the current study suggest some possibilities (Fig 9). The trees suggest that *Fusarium TRI* genes are more closely related than expected to those of *Beauveria* and *Cordyceps*, whereas *Trichoderma TRI* genes are more distantly related than expected to those of *Beauveria* and *Cordyceps*. In other studies, similar conflicts among phylogenetic trees have been attributed to lineage sorting and HGT [37,38,41]. If the conflicts were caused by lineage sorting, the *Beauveria*-*Cordyceps*, *Fusarium*, and *Trichoderma TRI* clusters could represent ancestral alleles or ancient paralogs that have been differentially inherited by these fungi such that *Beauveria*-*Cordyceps* and *Fusarium* inherited one allele (or paralog), and *Trichoderma* inherited another allele (or paralog). On the other hand, the close relationship of *TRI* cluster homologs in *Beauveria*-*Cordyceps* and *Fusarium* could have resulted from a HGT event between these two fungal lineages. The distant relationship of *Trichoderma* and *Beauveria*-*Cordyceps TRI* clusters could have also resulted from HGT events in which these two fungal lineages were recipients of distantly related *TRI* clusters. It is also possible that the distribution and phylogenetic relationships of the *TRI* cluster among the fungi examined here are a product of lineage sorting in some cases and HGT in others. Future studies aimed at identification and analysis of additional *TRI* cluster homologs in phylogenetically more diverse fungi could provide more definitive evidence for processes that have contributed to the distribution of the cluster.

The conflict between the *TRI22* tree (Fig 7) and the concatenated *TRI*-gene tree (Fig 9) with respect to the position of *Spicellum* suggests that the evolutionary history of *TRI22* homologs has differed from other *TRI* genes in this fungal genus. This conflict could be attributed to lineage sorting or HGT, but either process would have involved *TRI22* but not other extant *TRI* genes in *Spicellum*. Similar conflicts among *TRI* gene trees in closely related species of *Fusarium* were attributed to sorting of ancestral *TRI*-cluster alleles, which provide a mechanism to maintain production of two acetylated forms of deoxynivalenol [37]. It is unclear how a similar scenario could apply to *TRI22* given that it confers C4 hydroxylation, and therefore, alleles of *TRI22* are not likely to contribute to trichothecene structural diversity. It is noteworthy that the *Spicellum* strains were unusual among the fungi examined with respect to their position in the *TRI22* tree as well as the absence of *TRI4* in their genomes. Analyses of additional *Spicellum* species and their close relatives may provide insight into whether these two unusual features of *Spicellum TRI* genes resulted from related or independent events.

### Conclusions

Together, the results of the current and previous studies provide insights into evolutionary processes that have given rise to trichothecene structural diversity in fungi. The findings of the current study have facilitated inference of an ancestral trichothecene biosynthetic pathway (Fig 12) that is consistent with extant pathways that collectively yield over 150 structurally diverse trichothecene analogs. Knowledge obtained from functional analyses of *TRI* genes in *Fusarium* and *Trichoderma* has contributed significantly to insights of the evolutionary history of trichothecene biosynthesis. However, it is likely that functional analyses of *TRI* homologs in other fungi will provide evidence that refines or disproves evolutionary scenarios proposed in this study. Functional studies in other fungi should also lead to identification of additional *TRI* genes responsible for structural diversity of trichothecene analogs, including genes responsible for: i) C8 oxygenation in *Microcyclospora* and *Trichothecium*; ii) structural modification of the polyketide precursors of macrocyclic trichothecenes and harzianum A; and iii) formation of macrolide rings of macrocyclic trichothecenes. Future studies that sample numerous strains of the same species should provide evidence for whether gains and losses of genes are consistent across species.

Trichothecene structural diversity appears to have arisen largely from gain, loss, and changes in function of *TRI* genes, evolutionary processes that have also attributed to structural diversity of ergot alkaloids produced by fungi of the family Clavicipitaceae [56]. Our results also indicate that the presence of some substituents of trichothecenes have evolved independently in different lineages of fungi through gain of different genes with the same function. In addition, at least one trichothecene modification (C4 oxygenation) appears to have been lost, reacquired, and subsequently lost again during divergence of the *Fusarium TRI* cluster. Structural diversity of trichothecene analogs likely reflects differences in selection experienced by fungi that produce the analogs [37]. Thus, the cycle of loss, reacquisition, and subsequent loss of C4-oxygenated trichothecenes likely reflects changes in selection for biological activity conferred by the analogs. Trichothecene production contributes to pathogenesis of some fusaria on some hosts [10], and there is evidence that trichothecenes structural diversity in one lineage of *Fusarium* has been maintained by balancing selection [37]. It is not clear whether trichothecene production contributes to the pathogenicity of other fungi as well, but adaptation to pathogenesis on different plants and insects could provide selection pressure that has driven structural diversification of trichothecenes.

## Materials and methods

### Genome sequence and RNAseq analyses

Genome sequences of *B*. *bassiana* [29], *C*. *confragosa* [30], *F*. *graminearum* [31] and *Stachybotrys* species [21] have been reported previously, and were downloaded from the National Center for Biotechnology Information (NCBI) database. Genome sequences for all other fungi were generated as part of the current study, primarily with a MiSeq Illumina platform (Illumina, Inc.). In the initial genome sequence assemblies for the *Spicellum* and *Trichothecium* strains, almost every *TRI* gene was on a different contig, most of which were less than 5 kb in length. We partially overcame this limitation by generating a single genome sequence assembly from sequence reads generated with MiSeq, TruSeq (Illumina, Inc.), and an Ion Torrent Ion Proton Sequencer (Thermo Fisher Scientific Inc.). In the resulting assemblies, *TRI* genes were present on only five or six contigs (Fig 3, S2 Fig). To prepare DNA for genome sequencing, fungal strains were grown in YEPD medium (0.1% yeast extract, 0.1% peptone, 2% glucose) for 2 days at room temperature with shaking at 200 rpm. The exception to this was *M*. *tardicrescens* HJS 1936, which was grown in liquid YMG medium (0.4% yeast extract, 1% malt extract, 0.4% glucose) for 10 days as previously described [7]. Mycelia were harvested by filtration, lyophilized, ground to a powder, and genomic DNA was extracted using the ZR Fungal/Bacterial DNA MiniPrep kit (Zymo Research) or the chloroform-phenol method as previously described [70]. DNA sequencing libraries were prepared as follows. For the MiSeq platform, the Nextera XT DNA library Preparation Kit was used as specified by the manufacturer (Illumina). For the TruSeq platform, genomic DNA was first sonicated for four cycles with Diagenode Bioruptor system as specified by the manufacture to obtain 500-bp fragments (Diagenode). Sequencing libraries were prepared from the fragmented DNA with the TruSeq DNA LT Library Preparation Kit as specified by the manufacturer (Illumina). For the Ion Torrent Ion Proton Sequencer, the NEBNext Fast DNA Fragmentation & Library Prep Set for Ion Torrent was used as specified by the manufacturer (New England BioLabs).

Sequence reads obtained from each platform were processed and assembled using CLC Genomics Workbench (Qiagen Inc.). Gene predictions were done using the program Augustus [71] and FGENESH (Softberry, Inc., Mount Kisco, New York). All *TRI* and housekeeping genes obtained from the genome sequences and used in phylogenetic analyses were also manually annotated. The 18 *TRI* genes used as queries in BLAST analyses have been described in *Fusarium* [55], *Trichoderma* [17] and/or *Stachybotrys* [21]. BLASTx and BLASTn analyses were done against our in-house genome sequence database using CLC Genomics Workbench. Once contigs with *TRI* genes were identified in genome sequence assemblies, a portion of or the entire contig (depending on contig length) were subjected to gene prediction via FGENESH, and the resulting coding regions, as well as genomic sequences, were subjected to BLASTx analysis against the NCBI Non-redundant Protein Sequences database at NCBI to confirm the presence of *TRI* genes and to identify putative functions of other genes in the same region.

For RNAseq analysis, *Spicellum* and *Trichothecium* strains were grown in liquid YEPD medium for one, two, and three days, after which mycelia were harvested by filtration and lyophilized. RNA was isolated with the RNeasy method (Qiagen), and cDNA libraries were prepared with the MinElute Reaction Cleanup Kit (Qiagen). cDNA libraries were then sonicated for five cycles with Diagenode Bioruptor system (Diagenode) as specified by the manufacturer to obtain 100- to 300-bp fragments. Sequencing libraries were prepared from the sonicated DNA using a NEBNext Fast DNA Library Prep Set for Ion Torrent (New England BioLabs). The resulting library was then sequenced using Ion Torrent Ion Proton Sequencer platform (Thermo Fisher Scientific). The resulting sequence reads were analyzed using the RNA-Seq Analysis function in CLC Genomics Workbench.

### *TRI6* analysis

To confirm sequences of the three *TRI6* paralogs in *Trichothecium*, we amplified each paralog from three strains of *T*. *roseum* by PCR and sequenced the amplicons via Sanger Sequencing. PCR primers used for these amplifications are shown in S1 Table. The DNA polymerase GoTaq was used for amplification, and the conditions were those recommended by the manufacturer (Promega). Amplicons were purified using standard agarose gel electrophoresis and the UltraClean protocol (Mo Bio Laboratories). Amplicons were sequenced using BigDye Terminator version 3.1 and BigDye Xterminator Purification reagents (Thermo Fisher Scientific), and sequences were determined with a 3739 DNA Analyzer (Thermo Fisher Scientific). Sequences were viewed and edited using Sequencher (Gene Codes Corporation).

### Deletion and complementation of *Trichoderma TRI* genes

For deletion and complementation of *T*. *arundinaceum TRI* genes, previously described plasmid and protoplast-mediated transformation methods were used [17]. Deletion mutants and complemented deletion mutants were examined for their ability to produce harzianum A and other trichothecene analogs using the previously described two-step culture procedure [17].

*TRI3* deletion was accomplished by transformation with plasmid pΔtri3 that had been linearized with *Apa*I prior to transformation (**Fig A in** S1 File). Transformants were selected using 100 μg hygromycin B per mL of selection medium as previously described [17,72]. Transformants were analyzed by PCR with oligonucleotides Tarun-TrpC3/Tarun-compT35F (S1 Table) for the presence of a fragment expected to result from replacement of the *TRI3* coding region with the hygromycin resistance cassette (*hygB*). Transformants that yielded a PCR product were then analyzed by PCR to confirm the absence of *TRI3*. *TRI3* deletion was confirmed by Southern blot analysis using four hybridization probes (**Fig A in** S1 File). Based on these analyses, we concluded that transformants tri3.1, tri3.30, tri3.33 and tri3.48 were *tri3* deletion mutants (**Fig A in** S1 File).

*tri3* deletion mutant tri3.1 was complemented by transformation with plasmid pTCtri3-ble linearized with *Eco*RI (**Fig B in** S1 File). Transformants were selected using 75 μg phleomycin per mL of selection medium. Five transformants were analyzed by PCR for the presence of *TRI3* and the phleomycin resistance cassette (*bleR*) with oligonucleotides Tarun-T3int3/Tarun-T3int5 and Tarun-Phleo-3/Tarun-Phleo-4, respectively (S1 Table). Three transformants that yielded amplicons from both primer pairs were analyzed by Southern blot analysis with two hybridization probes to confirm the presence of *TRI3* and *bleR* (**Fig B in** S1 File).

*TRI17* deletion was accomplished by transformation with plasmid pΔtri17 linearized with *Xho*I prior to transformation (**Fig C in** S1 File). Transformants were selected with hygromycin as described above and analyzed by PCR with oligonucleotides Tarun-Db741/Tarun-Db742 to detect *hygB*, and with oligonucleotides Tarun-pks-F/Tarun-pks-R to test for the absence of *TRI17* (S1 Table). Transformants that yielded the appropriate amplicons were analyzed further by PCR with oligonucleotides Tarun-5-disrT/Tarun-TtrpC-disrT (S1 Table) to test for a fragment expected to result from replacement of the *TRI17* coding region with *hygB*. Selected transformants were also analyzed by Southern blot analysis to confirm deletion of *TRI17* (**Fig C in** S1 File). Based on these analyses, we concluded that transformants tri17.96, tri17.109 and tri17.139 were *tri17* deletion mutants.

*tri17* deletion mutant tri17.139 was complemented by transformation with plasmid pTCtri17-ble linearized with *Not*I (**Fig D in** S1 File). Transformants were selected with 100 μg of phleomycin per mL of selection medium. Transformants were analyzed by PCR for the presence of *TRI17* and *ble* with oligonucleotides Tarun-pks-F/Tarun-pks-R and Tarun-Phleo-3/Tarun-Phleo-4, respectively (S1 Table). A subset of six transformants that yielded amplicons from both primer pairs were subjected to Southern blot analysis with three hybridization probes to confirm the presence of *TRI17* and *bleR* (**Fig D in** S1 File).

*Tri17* deletion mutant tri17.139 was also complemented with a plasmid, pTCMrtri17-ble, carrying *M*. *roridum TRI17* and that had been linearized with *Eco*RI (**Fig E in** S1 File). Transformants were selected as indicated above, and analyzed by PCR for the *M*. *roridum TRI17* and *bleR* genes (**Fig E in** S1 File). Based in the PCR results, transformants tri17.MrT17.C3, .C4, .C5, and .C13 were selected for further studies.

### Heterologous expression of *B*. *bassiana TRI* genes

To express *TRI4* and *TRI22* from *B*. *bassiana* in *F*. *verticillioides*, the coding region (with intron sequences intact) plus ~500-bp of the 3’ flanking region of each gene was fused to the promoter sequence of the translation elongation factor 1α (*TEF1*) gene from *Aureobasidium pullulans*. This was done using a previously described PCR-based fusion method [26]. The primers used for the PCR are shown in S1 Table, and DNA polymerases used were iProof High Fidelity DNA polymerase (Bio-Rad Laboratories) for *TRI4* and Platinum *Taq* DNA Polymerase High Fidelity (Thermo Fisher Scientific) for *TRI22*. For the fusion PCR, the *A*. *pullulans TEF1* promoter was amplified from plasmid pTEFEGFP [73] with a reverse primer that consisted of approximately 22 nucleotides of the 3’ end of the *TEF1* promoter and approximately 22 nucleotides of the 5’ end of the *B*. *bassiana TRI4* (or *TRI22*) coding region. Likewise, the *B*. *bassiana TRI4* (or *TRI22*) coding region and 3’ flanking region were amplified from genomic DNA of *B*. *bassiana* strain ARSEF 2860 using a forward primer that was essentially the reverse complement of the primer described above; that is, the primer included sequences of both the 3’ end of the *A*. *pullulans TEF1* promoter and 5’ end of the *B*. *bassiana TRI4* (or *TRI22*) coding region. The two amplicons were then annealed and amplified by PCR without primers to generate the chimeric *TEF1* promoter::*TRI4* (or *TRI22*) construct, which was then further amplified with nested primers as previously described [26]. The fusion amplicons were cloned into the PCR cloning vector pCR-XL1 TOPO (Thermo Fisher Scientific) and sequenced to confirm that PCR amplification did not introduce nucleotide errors. The geneticin resistance gene (*genR*) was then introduced into the resulting vector via *Not*I digestion using standard molecular biology protocols as described previously [26].

The *TEF1* promoter::*TRI4* (or *TRI22*)-*genR* vector was then introduced into *F*. *verticillioides* strain M-3125 via a protoplast-mediated transformation protocol as previously described [72]. The presence of the construct in *F*. *verticillioides* transformants was confirmed by PCR with primer combinations used to generate the *TEF1* promoter::*TRI* constructs.

For precursor feeding experiments, *F*. *verticillioides* transformants were inoculated into liquid YEPD medium. Trichodiene or isotrichodermin (3-*O*-acetyl EPT), obtained from previous studies [74,75], were dissolved in acetone and then added to cultures at a final concentration of 250 μM. The final concentration of acetone was less than 1%. The cultures were incubated in the dark at 28 °C with shaking (200 rpm). After six days, cultures were extracted with ethyl acetate, and the extracts were analyzed by GC-MC as described below.

To express the *B*. *bassiana TRI101* in *S*. *cerevisiae*, the *TRI101* coding region was amplified from *B*. *bassiana* ARSEF 2860 genomic DNA using primers indicated in S1 Table and iProof High Fidelity DNA polymerase (Bio-Rad Laboratories) following the manufacturer’s recommendations. The PCR product was gel purified using the QIAEX II Gel Extraction Kit (Invitrogen), treated with 1000 units of Taq DNA polymerase (Qiagen) to add A overhangs to the amplicon 3’ ends, and then cloned into pYES2.1 using the TOPO TA Yeast Expression Kit (Invitrogen). In the resulting plasmid, the *TRI101* coding region was fused to the *GAL1* promoter and termination sequence. The cloned *TRI101* was sequenced to confirm that PCR amplification did not introduce errors, and then the plasmid was introduced into an *ayt1* mutant of *S*. *cerevisiae* (strain YLL063C; GE Healthcare Dharmacon) using the TRAFO protocol [76]. For feeding studies, yeast transformants were grown in the dark at 28 °C with shaking (200 rpm) on minimal medium [77], supplemented with leucine (1g/L), methionine (200 mg/L) and histidine (200 mg g/L. After 3 days, cultures were centrifuged and the pellet was re-suspended in YGal medium (1% yeast extract, 2% peptone, 2% galactose) to induce *TRI101* expression. Isotrichodermol (3-hydroxy EPT) was added to the cultures at a final concentration of 250 μM. After an additional four-day incubation in the dark at 28 °C with shaking (200 rpm), cultures were extracted with ethyl acetate and analyzed with GC-MS as described below.

### Chromatography and mass spectrometry

HPLC, LC-MS/MS and GC-MS were used to monitor trichothecenes and other metabolites produced by fungal strains. Harzianum A (HA), which is not detectable by GC-MS analysis, was detectable and quantified by HPLC and/or LC-MS/MS analysis. For HPLC analysis of harzianum A, *T*. *arundinaceum* cultures were extracted with an equal volume of ethyl acetate. The upper phase was recovered and evaporated to dryness in a rotary evaporator at room temperature, and then redissolved in acetonitrile at 10% of the original volume. After a final 1/5 dilution, a 20 μL aliquot of the resulting sample was used for HPLC analysis. The HPLC system consisted of a Waters 600 HPLC connected to a 996 Photodiode Array Detector (Waters Corporation) [17]. The column was a Waters YMC analytical column (150 mm length, 4.6 mm internal diameter). The initial mobile phase was 40:60 acetonitrile:water with 0.1% trifluoroacetic acid, and had a flow rate of 1 mL/min. After 30 min, the mobile phase was adjusted to 100% acetonitrile over 10 min, held for 5 min, and then returned to the initial condition [17].

For both GC-MS and LC-MS/MS analyses, 5-mL aliquots of liquid cultures (fungal biomass and medium) were combined with 2 mL ethyl acetate, and mixed vigorously for 5 min. The ethyl acetate phase was recovered and used directly in GC-MS and LC-MS/MS analyses. The GC-MS system consisted of a Hewlett Packard 6890 gas chromatograph fitted with a HP-5MS column (30 m length, 0.25 mm film thickness) and a 5973 mass detector (Hewlett Packard). The carrier gas was helium with a 20:1 split ratio and a 20 mL/min split flow. The column was held at 120 °C at injection, heated to 210 °C at 15 °C/min and held for 1 min, then heated to 260 °C at 5 °C/min and held for 8 min.

The LC-MS/MS system consisted of a ThermoDionex Ultimate 3000 UPLC fitted with a Phenomenex Kinetex F5 column (150 mm length, 2.1 mm diameter, 1.7 μm particle size) connected to the ionspray interface of an ABSciex Qtrap 3200 mass spectrometer operated in negative mode. The chromatographic separation was done with a 200 μL/min gradient flow of water and acetonitrile with 0.3% ammonium acetate. The separation utilized a gradient from 35 to 95% aqueous acetonitrile over 10 min. The column was held at 50 °C for the entire analysis.

In the analytical systems described above, identification of trichothecene analogs was confirmed by comparing their chromatographic retention times and, in MS analyses, molecular masses and mass spectra to those of standards. Novel trichothecenes were purified and their structures were determined by ^1^H and ^13^C NMR spectrometry as previously described [17,78].

### Phylogenetic analyses

For phylogenetic analyses of *TRI* and housekeeping genes, coding region sequences were aligned via the computer program MUSCLE as implemented in MEGA7 [79]. In general, nucleotide sequences were translated to amino acid sequences, aligned, and then converted back to nucleotide sequences before further analysis. Aligned sequences were subjected to maximum likelihood analysis using the computer program IQ-Tree version 1.5.5 [80]. In analyses with IQ-Tree, the best substitution model was determined by the program prior to tree building. Some alignments (e.g., *TRI6*) were also subjected to maximum parsimony analysis using MEGA7, and for some genes (e.g., *TRI17* and *TRI101*) alignments of deduced amino acid sequences were analyzed in addition to nucleotide sequences. For *TRI* genes, potential conflicts within sets of concatenated gene sequences were initially assessed using the Homogeneity Partition test as implemented in PAUP version 4.0a [81]. Bootstrap [80], SH [35], and AU [36] analyses, as implemented in IQ-Tree version 1.5.5, were also used to assess whether trees inferred from different sequences were significantly different. In preliminary analyses of individual *TRI* genes, it was not clear which strain/species should be used to root trees. To assess the most appropriate *TRI*-gene root in each tree, we conducted BLASTx analyses against the GenBank non-redundant database to identify genes that were distantly related to but would still align to *TRI* genes [82]. Four to six of the best BLAST hits were then used for alignment and tree building with the corresponding *TRI* gene, and thereby obtain information on which *TRI* gene homolog(s) was most basal.

## Supporting information

S1 File Strategy and molecular genetic analysis for deletion and complementation of *TRI3* and *TRI17* in *Trichoderma arundinaceum* strain IBT 40837 (Ta37). (DOCX)

S2 File Assessment of significance of conflicts among the concatenated *TRI3*-*TRI5*-*TRI14* tree and single-*TRI*-gene trees. (DOCX)

S3 File Assessment of concatenation of housekeeping gene sequences to infer a species phylogeny. (DOCX)

S1 Fig Phylogenetic analysis and rational for designation of *TRI22* as a distinct gene from *TRI11*. (DOCX)

S2 Fig Content and arrangement of genes at *TRI* loci in selected fungi. In the diagrams (above), green arrows represent known *TRI* genes, and gray arrows represent genes that are unique to a region in a particular genus. Numbers below arrows are locus tag numbers (with five-letter prefix, the underscore, and in some cases a zero omitted). The tables below the diagrams include predicted functions of genes based on Blast2Go analysis and supplemented with manual blast analysis in some cases. Tables also include information on contigs on which the genes occur. In the tables, genes above and below a double line are on different contigs. **A.** *Beauveria bassiana*; **B.** *Cordyceps confragosa*—orange arrows represent genes that are common to the *TRI* cluster locus in *Beauveria* and *Cordyceps*; **C.** *Microcylospora tardicrescens*; **D.** *Myrothecium roridum*—purple arrows represent genes that are common to *TRI* loci in *Myrothecium* and *Stachybotrys chartarum*; **E.** *Spicellum ovalisporum*; **F.** *Spicellum roseum*; **G.** *Stachybotrys chartarum*; **H.** *Trichoderma arundinaceum*; and **I.** *Trichothecium roseum*.
(PPTX)

S3 Fig BLASTx result using a 558-nucleotide sequence from the *TRI3*-*TRI6* intergenic region of *Spicellum roseum* DAOM 209012 as a query against the non-redundant protein sequence database at NCBI, with the organism option set at “Trichothecium (taxid:231006)”. The nucleotide sequence of the query is shown below the BLASTx results.
(PPTX)

S4 Fig Total ion chromatograms from gas chromatography-mass spectrometry analysis of an extract from a seven-day-old YEPD culture of *Spicellum roseum* strain DAOM 209012. Peaks corresponding to trichodermol (4-hydroxy EPT), trichodermin (4-*O*-acetyl EPT), and 8-deoxy trichothecin (4-*O*-butenoyl EPT) are shown. Based on mass spectral fragmentation patterns, the unlabeled peaks at 5 min and 6.1 min do not correspond to trichothecenes.
(PPTX)

S5 Fig Liquid chromatography-tandem mass spectrometry analysis of the *T*. *arundinaceum tri17* mutant tri17.139 complemented with the *M*. *roridum TRI17* homolog. Results are presented for the wild-type progenitor strain (blue trace) and complementation strains tri17.MrT17.C3 (black trace) and tri17.MrT17.C4 (red trace). The trace for *tri17* mutant strain tri17.139 (purple trace) does not rise above the base line. Identity of harzianum A in samples was confirmed by comparison of mass spectra to the spectrum of a purified harzianum A standard, the identity of which was confirmed by ^1^H and ^13^C NMR. Strains were grown in YEPD medium for one week.
(PPTX)

S6 Fig Mass spectral and NMR characterization of 4-hydroxy isotrichodermin (3-*O*-acetyl-4-hydroxy EPT) produced in YEPD cultures of *F*. *verticillioides* expressing the *B*. *bassiana TRI22* homolog and to which exogenous isotrichodermin was added. **Fig A**: Total ion chromatogram of an ethyl acetate extract of the culture (above), and mass spectrum of 4-hydroxy isotrichodermin (below). **Fig B**: ^13^C (left) and ^1^H (right) NMR spectra of 4-hydroxy isotrichodermin.
(PPTX)

S7 Fig Phylogenetic analysis of *TRI3*, *TRI5* and *TRI14* sequences to determine an appropriate root for *TRI* gene trees. In the tree shown here, a distantly related homolog was selected for each of the three *TRI* genes. For *TRI3*, we selected a homolog of *TRI18* as the outgroup. We searched GenBank by BLASTx [75] to identify non-*TRI* gene homologs to use at outgroups in the *TRI5* and *TRI14* trees; the outgroups are represented by their GenBank accession numbers. To generate trees, deduced amino acid sequences of each gene were aligned with Muscle as implemented in MEGA7 [79], and trees were inferred using the maximum likelihood method with ultrafast bootstrapping [83] as implemented in IQ-Tree [73]. Numbers near branches are bootstrap values based on 1000 pseudoreplicates.
(PPTX)

S8 Fig Maximum likelihood trees inferred from coding region sequences of *TRI10*, *TRI12*, and *TRI18*. (PPTX)

S1 Table Oligonucleotide primers used in this study. (DOCX)
