# Supplementary material for: Evolution of structural diversity of trichothecenes, a family of toxins produced by plant pathogenic and entomopathogenic fungi
Source: PLoS Pathog. 2018 Apr 12;14(4):e1006946. doi: 10.1371/journal.ppat.1006946 (PMC5897003; doi:10.1371/journal.ppat.1006946)
Supplement: S1 File — (DOCX) [file ppat.1006946.s001.docx]

**S1 File:** Strategy and molecular genetic analysis for deletion and complementation of *TRI3* and *TRI17* in *Trichoderma arundinaceum* strain IBT 40837 (Ta37).

**
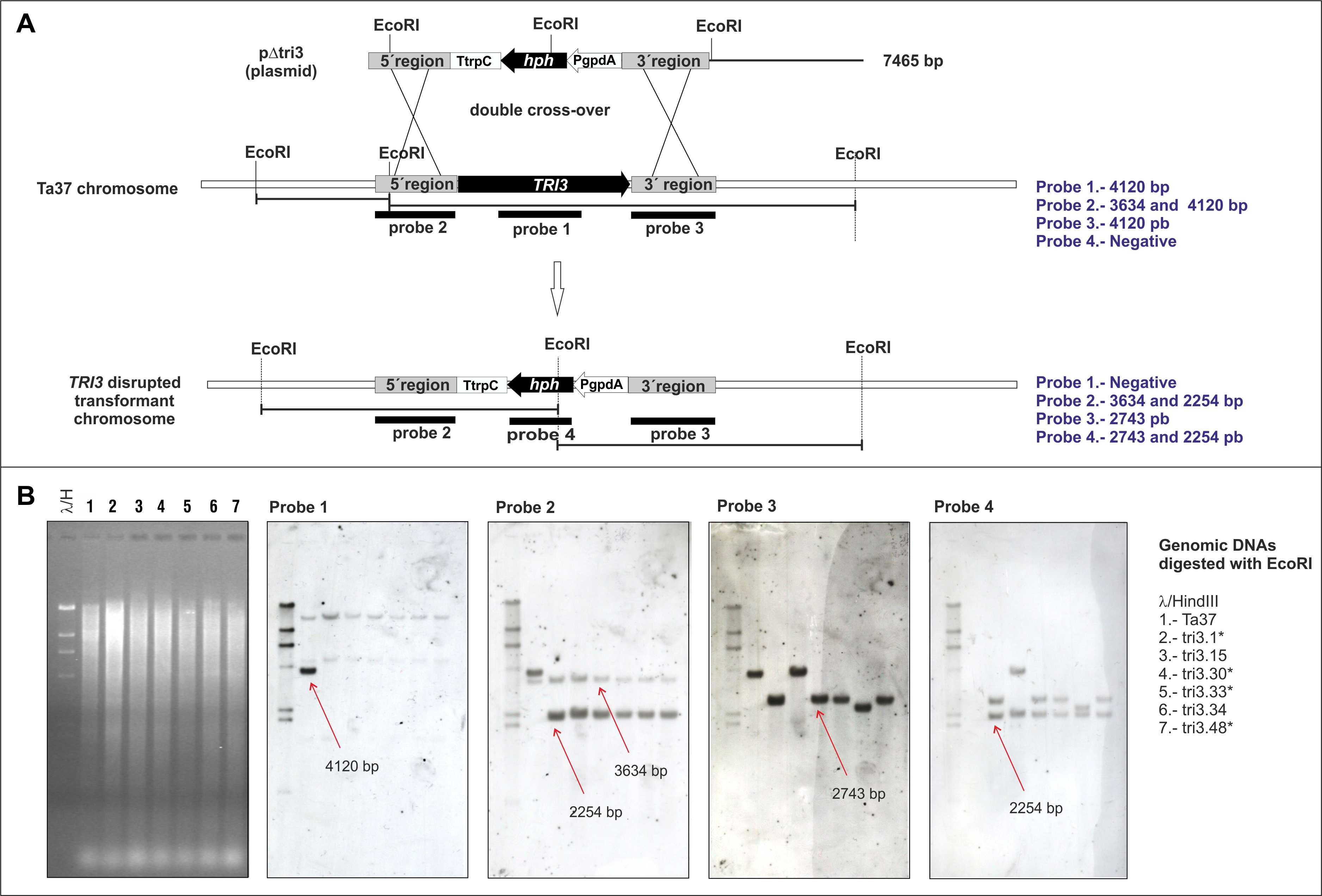
**

**Fig A**. Molecular strategy (**A**) and Southern blot analysis (**B**) for *TRI3* deletion in wild-type *Trichoderma arundinaceum* strain IBT 40837 (Ta37) with plasmid pΔtri3. In panel **A**, Hybridization probes used in Southern analyses are indicated with black bars, and sizes of expected bands resulting from *Eco*RI digestion of genomic DNA of the wild type and *tri3* deletion mutants are shown at the right. The hygromycin resistance cassette consists of: PgpdA - promoter region of the *Aspergillus nidulans* glyceraldehyde-3-phosphate dehydrogenase; TtrpC - *A. nidulans trpC* terminator region; *hph* - *E. coli* hygromycin B resistance gene. In panel **B**, some of the expected bands and their sizes are indicated with red arrows. On the right of panel **B**, transformants showing the expected bands for *tri3* deletion are indicated with an asterisk.

Construction of pΔtri3: 846-bp and 938-bp fragments corresponding to the 5´ and 3´ flanking regions, respectively, of the *TRI3* coding region were amplified with PCR using Pfu high fidelity polymerase (Fermentas, Vilnius, Lithuania) and oligonucleotides Tarun-TRI35F/Tarun-TRI35R and Tarun-TRI33F/Tarun-TRI33R, respectively (**S1 Table**). Amplicons were cloned into plasmid pSpark (Canvax, Córdoba, Spain) to yield pSpark-5’ and pSpark-3’. pSpark-3´ was digested with *Sac*II and *Eco*RV, and ligated with the 846 bp fragment corresponding to the 5´ *TRI3* region, which had been released from pSpark-5´ by *Sac*II-*Eco*RV digestion. The resulting plasmid, pSpark-T3-3´5´, was digested with *Eco*RV, dephosphorylated with alkaline phosphatase, and then ligated to *hygR* chimeric gene, which consists of the coding region of the hygromycyn phosphotransferase gene (*hph*) from *E. coli* fused to the *gpdA* promoter and *trpC* terminator sequences of *A. nidulans*. *hygR* had been released from plasmid pAN71 [1] by *Hind*III-*Sac*I digestion and treated with the Klenow fragment of DNA polymerase I (Klenow). The resulting plasmid, pΔtri3 (7465 bp), was used to delete the *TRI3* coding region by a double cross over strategy [2].

**
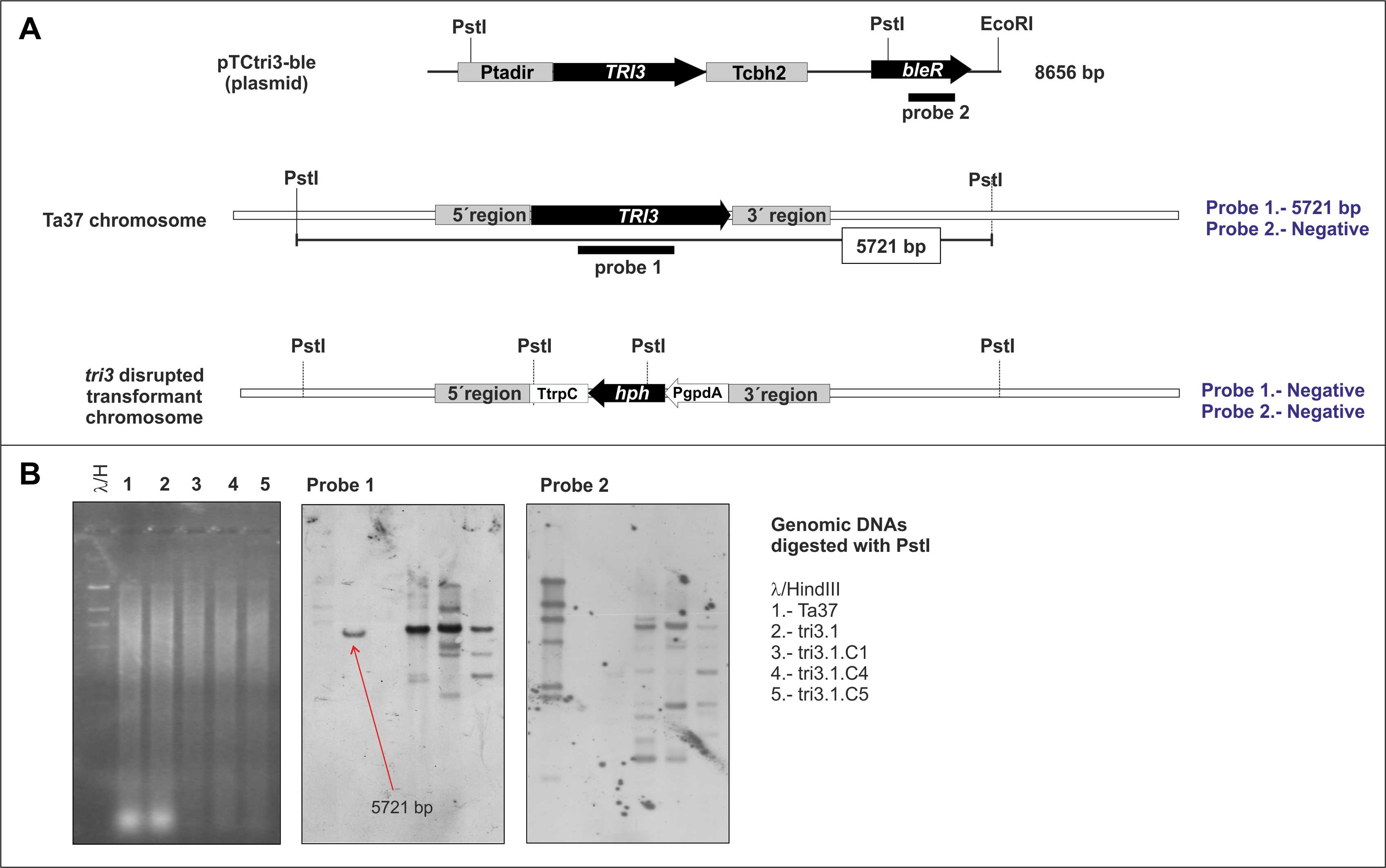
**

**Fig B**. Molecular strategy (**A**) and Southern blot analysis (**B**) for complementation of a *T. arundinaceum tri3* deletion mutant. Panel **A** shows maps of the linearized complementation plasmid pTCtri3-ble (8656 bp) used to express *T. arundinaceum* *TRI3* in the *tri3* mutant (top), the intact *TRI3* in *T. arundinaceum* strain IBT 40837 (middle); and the deleted *TRI3* gene in *tri3* mutant tri3.1(bottom). Probes used in the Southern analyses are indicated with black bars, and the sizes of expected bands resulting from *Pst*I digestion of genomic DNA of the wild type and *tri3* mutant are shown at the right. Plasmid pTCtri3-ble included the *T. arundinaceum TRI3* coding region (with intron sequences) fused to the *T. harzianum* TA gene promoter region (Ptadir) and the transcriptional terminator region (Tcbh2) of the *T. reesei* cellobiohydrolase 2 gene. pTCtri3-ble also included *bleR*, chimeric fungal-bacterial gene that confers resistance to the antibiotic phleomycin. *bleR*  consists of the following genetic elements: PpcbC - promoter region of the *Penicillium chrysogenum* isopenicillin N cyclase encoding gene; *ble* – coding region of the bleomycin/phleomycin resistance gene from *Streptoalloteichus hindustanus*; TCYC1 - Transcriptional terminator region of the *Saccharomyces cerevisiae* *CYC1* gene. In panel **B**, the expected band and its size for probe 1 in the wild type is indicated with a red arrow (**B**) Southern analysis of tri3-complemented Transformants.

Design and construction of plasmid pTCtri3-ble. A 1784-bp fragment corresponding to the wild-type *TRI3* coding region was amplified using Pfu DNA polymerase (EURx, Gdansk, Poland) and oligonucleotides Tarun-TRI3F/Tarun-TRI3R (**S1 Table**). The oligonucleotides were designed to include an *Nco*I restriction site. The amplicon was treated with polynucleotide kinase and cloned into plasmid pBluescript KS(+) (Stratagene, La Jolla, CA) that had been digested with *Eco*RV and treated with the FastAP enzyme (Thermo Scientific, Wilmington, DE). The amplicon was then subcloned into plasmid pTAcbh [3] by *Nco*I digestion. The resulting plasmid was digested with *Eco*RI, treated with Klenow (Thermo Scientific, Wilmington, DE), dephosphorylated with alkaline phosphatase and then ligated to *bleR* and *tadir* promoter sequence of *T. harzianum*, previously released from plasmid pJL43 [4] by *Bst*XI-*Xho*I digestion and treatment with Klenow (Thermo Scientific). The resulting plasmid, pTCtri3-ble (8656 bp) was used for complementation of the *tri3* mutant.


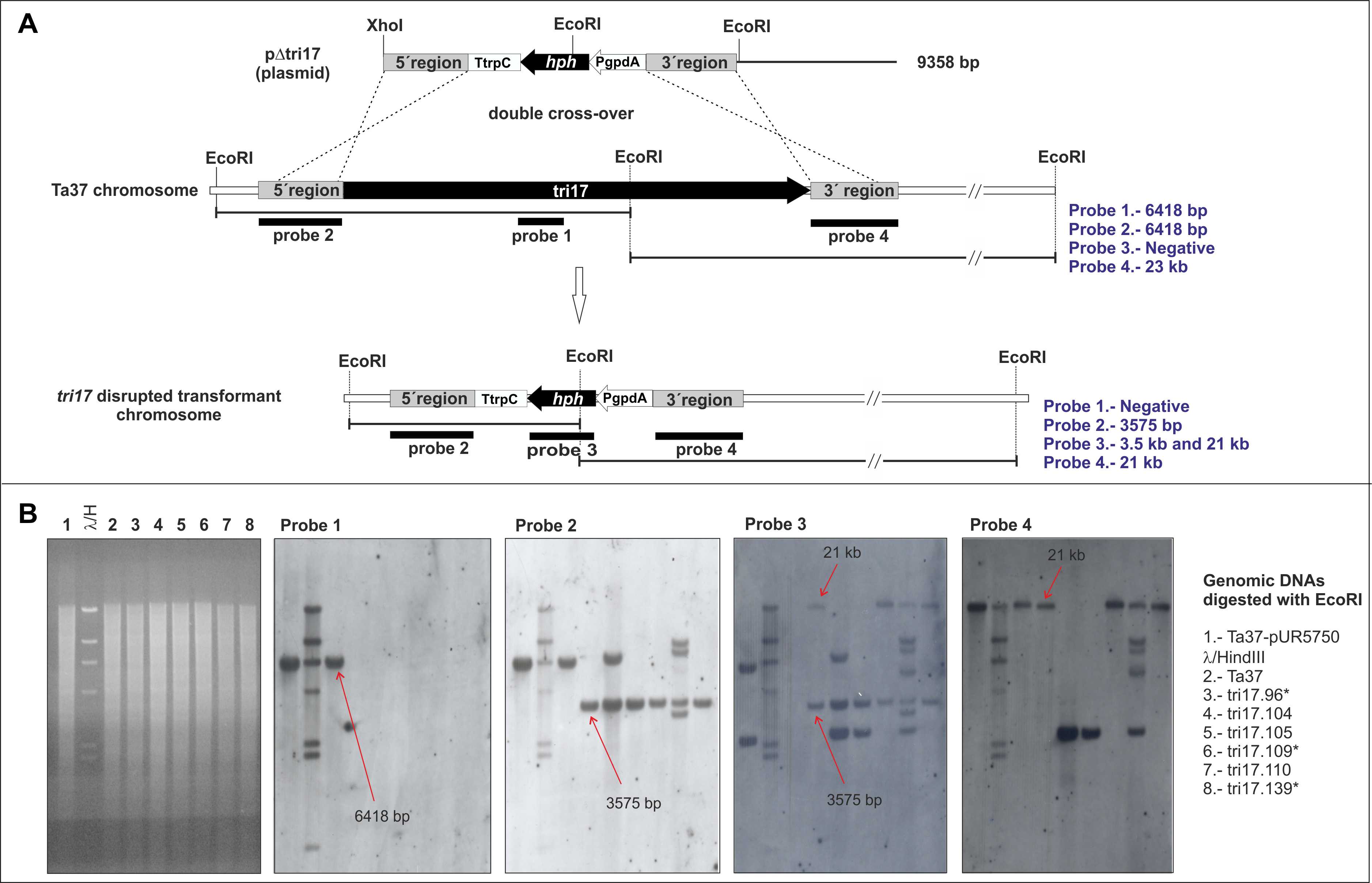


**Fig C**. Molecular strategy (**A**) and Southern blot analysis (**B**) for *TRI17* deletion in wild-type *Trichoderma arundinaceum* strain IBT 40837 (Ta37) with plasmid pΔtri17. Hybridization probes used in the Southern analyses are indicated with black bars, and the sizes of the expected bands resulting from digestion of genomic DNA with *Eco*RI are shown at the right. Genetic elements of the hygromycin resistance chimeric gene (*hygR*) are as described in **Fig A in S1 File**. In panel **B** some of the expected bands and their sizes are indicated with red arrows. Transformants showing the expected pattern for a *tri17* deletion are indicated with an asterisk to the right. Note: Disruption of *TRI17* was also confirmed by the genome sequence analysis of tri17.139 using the MiSeq Illumina platform (Illumina, Inc.).

Construction of pΔtri17: A 1309-bp fragment corresponding to the 5´ flanking region of the *TRI17* coding region was amplified by PCR from genomic DNA of wild type strain IBT 40837 using oligonucleotides Tarun-TRI1755X/Tarun-TRI1753EV (**S1 Table**). The amplified fragment was subcloned into plasmid pCRII-TOPO (Invitrogen, Carlsbad, CA), following the manufacturer instructions. The cloned fragment was released by *Xho*I-*Eco*RV digestion, and then gel purified. The same procedure was used to clone a 1379-bp fragment corresponding to the 3´ flanking region of the *TRI17* coding region. The 3’ fragment was amplified by PCR using oligonucleotides Tarun-TRI1735EV/Tarun-TRI1733Sc (**S1 Table**), and then cloned into pCRII-TOPO. The fragment was then released from the resulting vector by *Eco*RV-*Sac*I digestion. The 5’ and 3’ fragments were then ligated together into pBluescript KS+ digested with *Xho*I and *Sac*I to yield plasmid pTRI17-5b3b. The hygromycin resistance chimeric gene (*hygR*) cassette was extracted from pAN71 [1] by *Hind*III digestion, filled in with Klenow, and then digested with *Ecl*136II. The resulting 2690-bp fragment, containing the entire *hygR* chimeric gene, was ligated to pTRI17-5b3b that was digested with *Eco*RV and dephosphorylated with alkaline phosphatase. The resulting plasmid, pΔtri17, was used to delete the *TRI17* coding region by a double cross over strategy [2].


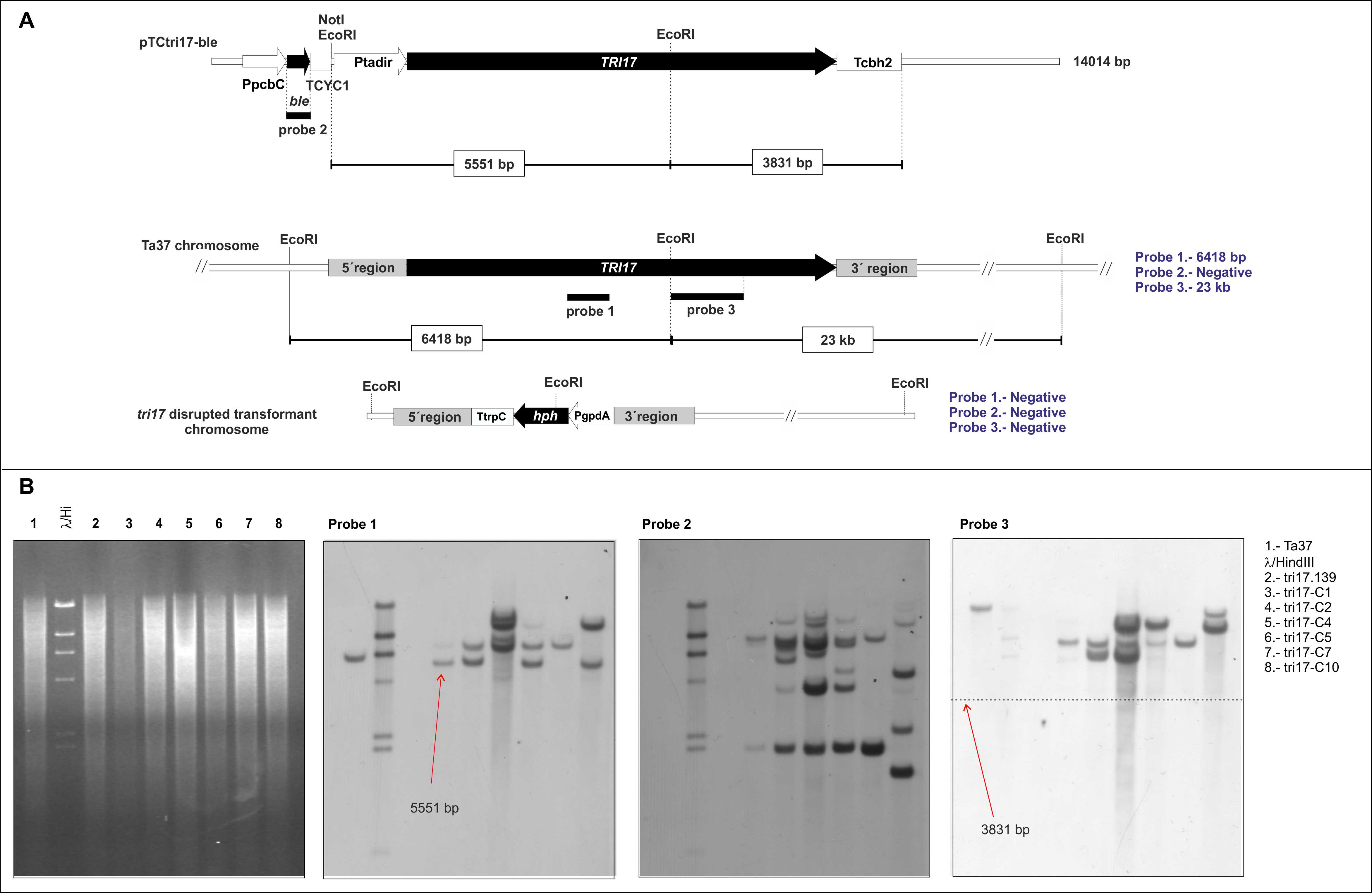


**Fig D**. Molecular strategy (**A**) and Southern blot analysis (**B**) for complementation of a *T. arundinaceum tri17* deletion mutant. Panel **A** shows maps of the linearized complementation plasmid pTCtri17-ble used to express *T. arundinaceum* *TRI17* in *tri17* mutant strain tri17.139 (top); the intact *TRI17* gene in wild-type *T. arundinaceum* (middle); and the deleted *TRI3* gene in mutant tri17.139 (bottom). Hybridization probes used in Southern analyses are indicated with black bars, and the sizes of the expected bands resulting from *Eco*RI digestion of genomic DNA of the wild type and *tri17* mutant are shown to the right. Genetic elements of the complementation vector are as described in **Fig B in S1 File**.In panel **B**, In the Southern blot for probe 1, the expected band of 5551 bp is indicated with a red arrow for one of the transformants. In the blot for probe 3, the dashed line indicates the minimal size expected to have the entire overexpression cassette integrated in the genome of transformants.

Design and construction of the plasmid pTCtri17-ble. In pTCtri17-ble, the *TRI17* gene was reconstituted from three separate fragments. The first fragment, Nt, was 379 bp in length and corresponded the to 5’ end of the genomic sequence of the *TRI17* coding region. The second fragment, Ct, was 1.5 kb and corresponded to the 3’ region of the coding region genomic sequence. The third fragment, M, was 5.8-kb *Bam*HI fragment that corresponded to the middle region of *TRI17*. M was released as a from phage 37F116, which was obtained from a genomic library of *T. arundinaceum* strain IBT 40837 that previously constructed in phage λDASHII (Stratagene, La Jolla, CA) [5]. Nt and Ct were amplified by PCR using the *Pfu* high fidelity polymerase (Fermentas, Vilnius, Lithuania) with the oligonucleotides Tarun-TRI17NtP/Tarun-TRI17NtB and Tarun-TRI17CtBN/Tarun-TRI17CtN, respectively (**S1 Table**). Ct PCR amplicon was cloned into plasmid pSpark (Canvax, Córdoba, Spain). Plasmid pTAcbh [3] was *Nco*I digested, dephosphorylated with alkaline phosphatase, and then ligated to fragment Ct, which had been released from the PCR cloning vector by *Nco*I digestion. The resulting plasmid, pTC-Ct-tri17, was partially digested with *Nco*I, filled in with Klenow and autoligated in order to remove the *Nco*I site at the 3’ end of fragment Ct but retain the *Nco*I site at the 5´ end. The resulting plasmid, pTC-Ct-tri17N*, was digested with *Nco*I, filled in with Klenow, digested with *Bam*HI, and then ligated to fragment Nt, previously digested with *Bam*HI. The resulting plasmid, pTC-CtNt-tri17, was digested with *Eco*RI, filled in with Klenow, dephosphorylated with alkaline phosphatase, and then ligated to the *bleR* chimeric gene to yield plasmid pTC-CtNt-tri17-ble. The cassette was present on a 1.5-kb fragment that had been released by *Bst*XI*-Xho*I digestion and treatment with Klenow from a plasmid pJL43 [4], in which the *Bam*HI site was previously removed by *Bam*HI digestion, klenow treatment and autoligation. The genetic elements of the *bleR* are described in **Fig B in S1 File.** Plasmid pTC-CtNt-tri17-ble was digested with *Bam*HI, dephosphorylated with alkaline phosphatase, and ligated to fragment M to yield plasmid pTCtri17-ble (14014 bp). The *TRI17* gene in pTCtri17-ble plasmid was sequenced to confirm that it did not contain any nucleotide errors.

**
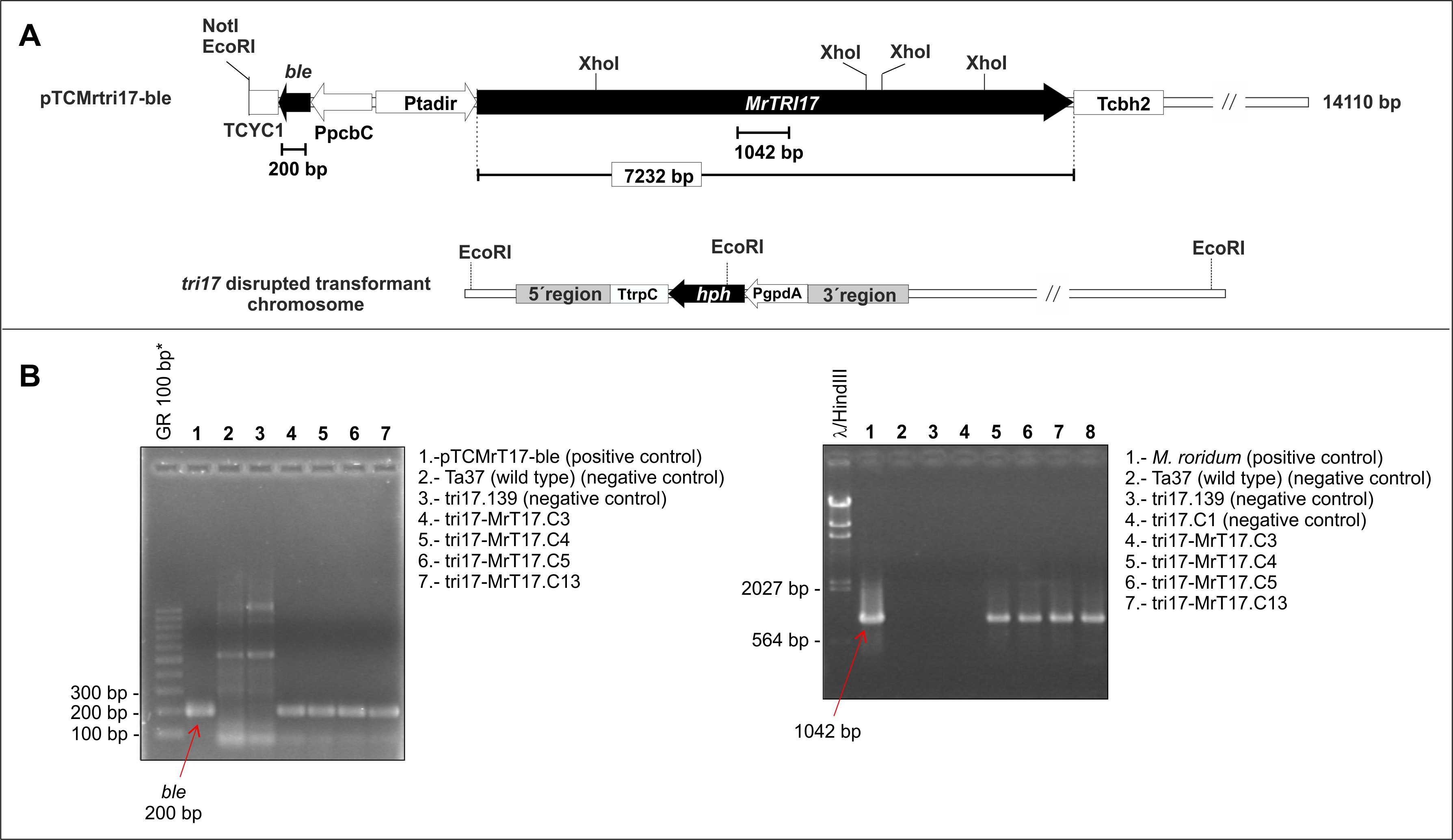
**

**Fig E**. Molecular strategy (**A**) and PCR analysis (**B**) of complementation of the *T. arundinaceum tri17* mutant with the *Myrothecium roridum TRI17* gene. Panel **A** shows maps of the plasmid, pTCMrtri17-ble, used to express *M. roridum* *TRI17* gene in *tri17* mutant strain tri17.139 (top); and the structure of the deleted *TRI17* in strain tri17.139 (bottom). pTCMrTRI17-ble contains the phleomycin resistance cassette and Ptadir promoter and Tcbh2 sequences described for other complementation plasmids (**Fig B in S1 File**). The fragments amplified by PCR to test for the presence of *ble* (200 bp) and the *M. roridum TRI17* (1042 bp) are indicated with black bars, and the lengths of the fragments are indicated below the bars. Panel **B** shows the results of PCR analyses for *ble* (left) and for *M. roridum TRI17* (right). The *ble* fragment was amplified with with oligonucleotides Tarun-Phleo-3 / Tarun-Phleo-4, and the *M. roridum TRI17* fragment was amplified with oligonucleotides Mrori-TRI17-4 / Mrori-TRI17-5rev (**S1 Table**). *GR 100 bp indicates the GeneRuler 100 bp Plus DNA Ladder (Thermo Scientific) used as molecular weight markers.

Construction of plasmid pTCMrT17-ble. To construct pTcMrT17-ble, a 7232-bp fragment consisting of the genomic sequence of the *M. roridum* *TRI17* coding region was amplified by PCR with oligonucleotides Mrori-TRI17-Nt and Mrori-TRI17-Ct (**S1 Table**) and Q5 DNA polymerase (New England Biolabs) following the manufacturer´s instructions. Plasmid pTAcbh [3] was *Nco*I digested, filled with Klenow of DNA polymerase I, dephosphorylated with alkaline phosphatase, and then ligated to the amplified *M. roridum TRI17* fragment, which was previously phosphorylated with the Polynucleotide Kinase. The resulting plasmid, pTC-TRI17-Mr, was digested with *Nco*I, filled with Klenow, dephosphorylated with alkaline phosphatase, and then ligated with a 1.5-kb fragment containing the phleomycin resistance chimeric gene (*bleR*) to yield plasmid pTCMrT17-ble (14110 bp). The phleomycin cassette had been released from plasmid pJL43 [4] by BstXI-XhoI digestion and treated with Klenow. The genetic elements of the *bleR* are as described in **Fig B in S1 File**. The entire *M. roridum TRI17* coding region present in pTCMrtri17-ble was sequenced to confirm the absence of PCR-induced mutations.

**REFERENCES**

1. Punt PJ, Oliver RP, Dingemanse MA, Pouwels PH, van den Hondel CA (1987) Transformation of *Aspergillus* based on the hygromycin B resistance marker from *Escherichia coli*. Gene 56: 117-124.

2. Casqueiro J, Gutierrez S, Banuelos O, Hijarrubia MJ, Martin JF (1999) Gene targeting in *Penicillium chrysogenum*: disruption of the *lys2* gene leads to penicillin overproduction. J Bacteriol 181: 1181-1188.

3. Cardoza RE, McCormick SP, Malmierca MG, Olivera ER, Alexander NJ, et al. (2015) Effects of trichothecene production on the plant defense response and fungal physiology: overexpression of the *Trichoderma arundinaceum* *tri4* gene in *T. harzianum*. Appl Environ Microbiol 81: 6355-6366.

4. Fierro F, Kosalkova K, Gutierrez S, Martin JF (1996) Autonomously replicating plasmids carrying the *AMA1* region in *Penicillium chrysogenum*. Curr Genet 29: 482-489.

5. Cardoza RE, Malmierca MG, Hermosa MR, Alexander NJ, McCormick SP, et al. (2011) Identification of loci and functional characterization of trichothecene biosynthesis genes in filamentous fungi of the genus Trichoderma. Appl Environ Microbiol 77: 4867-4877.
