# Supplementary material for: Evolution of structural diversity of trichothecenes, a family of toxins produced by plant pathogenic and entomopathogenic fungi
Source: PLoS Pathog. 2018 Apr 12;14(4):e1006946. doi: 10.1371/journal.ppat.1006946 (PMC5897003; doi:10.1371/journal.ppat.1006946)
Supplement: S2 File — (DOCX) [file ppat.1006946.s002.docx]

**S2 File: Assessment of significance of conflicts among the concatenated *TRI3*-*TRI5*-*TRI14* tree and single-*TRI*-gene trees.**

We assessed significance of conflicts for only *TRI10*, *TRI18*, *TRI22,* and *TRI101*; that is, the individual *TRI*-gene trees that had one or more well-supported branches that conflicted with one or more branches in the concatenated *TRI3­*-*TRI5*-*TRI14* tree. We used the Shimodaira-Hasegawa (SH) [1] and Approximately Unbiased (AU) [2] tests to assess the significance of the conflicts. For each assessment, a maximum likelihood tree was inferred using concatenated coding region sequences of *TRI3­*, *TRI5* and *TRI14* from a subset of taxa with homologs of each gene of interest (i.e., *TRI10*, *TRI18*, *TRI22,* and *TRI101*). With the exception of the absence of taxa, the concatenated *TRI3­*-*TRI5*-*TRI14* trees inferred from taxa subsets did not conflict with the tree inferred from all taxa. Therefore, only the tree inferred from all taxa is shown here (**Fig A**). Constrained trees for genes of interest were generated to conform to: 1) the entire topology of the concatenated *TRI3­*-*TRI5*-*TRI14* tree, or 2) individual branches within the concatenated *TRI3­*-*5*-*14* tree; in **Fig A** these branches are labeled as Branches A, B and C.

The result of SH and AU tests indicated that the constrained trees for *TRI22* or *TRI101* were significantly worse than the unconstrained trees (**Table A**). This, in turn, indicates that the conflicts between the *TRI22* or *TRI101* gene trees and the concatenated *TRI3­*-*5*-*14* tree were significant. In contrast, the SH and AU test results indicated that the conflicts observed for *TRI10* or *TRI18* tree were not significant (**Table A**). Evolutionary scenarios to explain the conflicts for *TRI22* and *TRI101* are described in the Discussion section of the accompanying manuscript.

**Table A:** Results of Shimodaira-Hasegawa and Approximately Unbiased tests to assess branch conflicts between the concatenated *TRI3­*-*5*-*14* tree and individual *TRI*-gene trees. Significant p-values (p < 0.05) are indicated with an asterisk (*). p-SH and p-AU are the p-values from the Shimodaira-Hasegawa test and Approximately Unbiased test, respectively. The positions of Branches A, B and C in the concatenated *TRI3­*-*5*-*14* tree are indicated in **Fig A**.

**Tree/Branch logL deltaL p-SH p-AU**

***TRI10***

Unconstrained -10379.561 0.000 1.0000 0.6470

Entire *TRI3-5-14* tree -10380.866 1.306 0.3611 0.3554

*TRI3-5-14* Branch B -10380.866 1.306 0.3611 0.3538

***TRI18***

Unconstrained -9274.666 0.000 1.0000 0.8835

Entire TRI3-5-14 tree -9274.670 0.004 0.0795 0.1381

TRI3-5-14 Branch A & B -9274.670 0.003 0.1492 0.1555

***TRI22***

Unconstrained -11406.508 0.000 1.0000 1.0000

Entire TRI3-5-14 tree -11483.214 76.706 0.0000* 0.0000*

TRI3-5-14 Branch C -11470.517 64.008 0.0000* 0.0001*

***TRI101***

Unconstrained -13102.572 0.000 1.0000 0.9898

Entire TRI3-5-14 tree -13122.347 19.774 0.0189* 0.0190*

TRI3-5-14 Branch A -13121.006 18.434 0.0082* 0.0050*

**Fig A:** Maximum likelihood tree inferred from concatenated coding region sequences of *TRI3­*, *TRI5* and *TRI14* (i.e., the concatenated *TRI3­*-*5*-*14* tree). This is the same *TRI3­*-*5*-*14* tree that is shown in **Fig 3** and **9** of the accompanying manuscript.


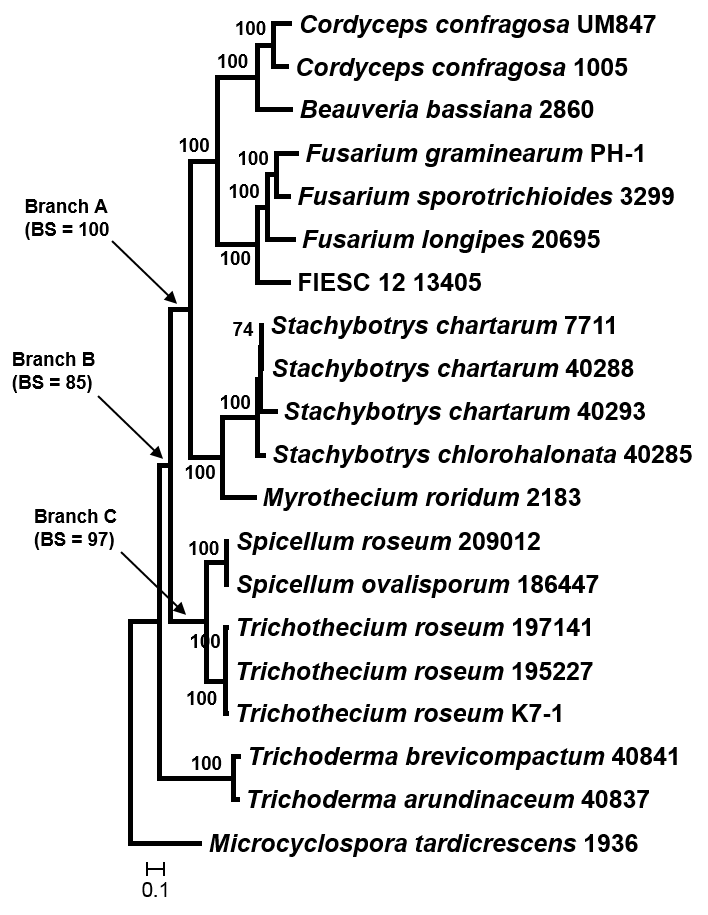


**References cited in S2 File**

1. Shimodaira H, Hasagawa M (1999) Multiple comparisons of log-likelihoods with applications to phylogenetic inference. Mol Biol Evol 16: 1114-1116.

2. Shimodaira H (2002) An approximately unbiased test of phylogenetic tree selection. Syst Biol 51: 492-508.
