# Supplementary material for: Evolution of structural diversity of trichothecenes, a family of toxins produced by plant pathogenic and entomopathogenic fungi
Source: PLoS Pathog. 2018 Apr 12;14(4):e1006946. doi: 10.1371/journal.ppat.1006946 (PMC5897003; doi:10.1371/journal.ppat.1006946)
Supplement: S3 File — (DOCX) [file ppat.1006946.s003.docx]

**S3 File: Assessment of concatenation of housekeeping gene sequences to infer a species phylogeny.**

In the accompanying study, we inferred a species phylogeny from the concatenated sequences of 20 housekeeping genes (**Table A**, **Fig A**). In this 20-gene tree, relationships between all genera in the Hypocreales were supported by bootstrap (BS) values of 92 – 100%. To assess whether the 20-gene tree was likely to be representative of a species phylogeny (**Fig A**), we examined trees inferred from each gene individually to identify supported branches (BS > 70%) that conflicted with the 20-gene tree. In most single-gene trees, deeper branches among lineages had poor bootstrap support (BS < 70) (**Fig B**). However, trees inferred from nine individual genes (*CPR1*, *DPA1*, *FAS1*, *KU70*, *LCB1*, *LCB2*, *TEF1*, *TOP1*, and *TUB2*) had one or two supported branches (BS > 70%) that conflicted with branches in the 20-gene tree (**Fig B**). Collectively, these nine single-gene trees had a total of 11 branches that conflicted with the 20-gene tree; eight of the conflicting branches occurred in only one tree, and one conflicting branch occurred in three trees (*DPA1*, *FAS1* and *LCB2*). Thus, the conflicting branches described above represent a relatively small percentage of branches that occur collectively in the single gene trees.

We subjected alignments of the nine genes that resulted in branches that conflicted with the 20-gene tree to the Shimodaira-Hasegawa (SH) [1] and Approximately Unbiased (AU) [2] tests. In these tests, we assessed whether constraining the topology of a single-gene tree to the topology of the 20-gene tree resulted in a significantly worse tree than the unconstrained tree for each gene. The results of both tests indicated that only four genes (*CPR1*, *DPA1*, *LCB2*, and *TEF1*) yielded constrained trees that were significantly worse (p < 0.1) than their corresponding unconstrained trees (**Table B**). Thus, although *FAS1*, *KU70*, *LCB1*, *TOP1*, and *TUB2* yielded single-gene trees with branches that conflicted with the 20-gene tree, the conflicts were not significant in assessments using the SH and AU tests.

We also used the SH and AU tests to assess whether constraining the 20-gene tree to include a conflicting branch from a single-gene tree was significantly worse than the unconstrained 20-gene tree. In this analysis, the results of the SH and AU tests were not always consistent (**Table C**). The SH text indicated that constraining the 20-gene tree to include a conflicting branch (BS > 70%) from the *CPR1*, *KU70*, *LCB1*, and *TEF1* single-gene trees resulted in a significantly worse tree than the unconstrained tree. In addition, the AU test indicated that all constrained trees were worse than the unconstrained tree.

To assess how inclusion of gene sequences that yielded the conflicting branches described above affected the proposed species phylogeny, we compared trees inferred from three sets of concatenated housekeeping genes: a tree inferred from concatenated sequences of all 20 housekeeping genes; an 11-gene tree inferred from concatenated sequences of only genes that yielded a single-gene tree that did not conflict with the 20-gene tree (conflicts within *Fusarium* were excluded); and a 16-gene tree inferred from concatenated sequences of all genes except for those (*CPR1*, *KU70*, *LCB1*, and *TEF1*) that yielded a p value less than 0.1 in the SH and AU test (**Table B)**. The three resulting trees did not have conflicting branches (**Fig A**). All branches were supported (BS = 87 – 100%), but support for some branches differed among the trees. Together, the multiple phylogenetic assessments described above suggest that the 20-gene tree provides a reasonable estimate of the species phylogeny.

**Table A:** Housekeeping genes used to infer a species phylogeny for fungi with trichothecene biosynthetic loci. For the trees shown in **Fig A,** all of the genes listed here were used to infer the 20-gene tree, while the closed and open circles indicate genes used to infer the 16- and 11-gene trees, respectively.

**Gene Predicted Protein Product CDS length (bases)**

*ACT1*●🞅 Actin 1494

*CPR1* Cytochrome P450 Reductase 2097

*DPA1* DNA Polymerase Alpha Subunit 4596

*DPD1*●🞅 DNA Polymerase Delta Subunit 3408

*DPE1*●🞅 DNA Polymerase Epsilon Subunit 6822

*FAS1*● Fatty Acid Synthase Alpha Subunit 5628

*FAS2*●🞅 Fatty Acid Synthase Beta Subunit 6330

*KU70*● ATP-dependent DNA helicase II 2052

*LCB1*● Sphinganine Palmitoyl Transferase Subunit 1 1677

*LCB2* Sphinganine Palmitoyl Transferase Subunit 2 2190

*PGK1*●🞅 Phosphoglycerate Kinase 1266

*RPB1*●🞅 RNA Polymerase Largest Subunit 5499

*RPB2*●🞅 RNA Polymerase 2nd Largest Subunit 3897

*TEF1* Translation Elongation Factor 1-alpha 1410

*TOP1*● Topoisomerase 2910

*TPS1*●🞅 Trehalose Phosphate Synthase 1632

*TSR1*●🞅 Ribosomal biogenesis protein 2547

*TUB1*●🞅 Tubulin alpha subunit 1353

*TUB2*● Tubulin beta subunit 1353

*UBT1*●🞅 Ubiquitin Thiolesterase 2691

**Table B:** Results of the SH and AU tests as implemented in IQ-Tree [3]. In these tests, the topologies of trees inferred from single gene alignments were constrained to the topology of the tree inferred from concatenated sequences of 20 housekeeping genes (i.e., species phylogeny). p values less than 0.1 are highlighted in gray. Sequences were aligned using Muscle as implemented in MEGA7 [4].

**Gene Tree logL deltaL p-SH p-AU**

*CPR1* Unconstrained -15788.012 0.000 1.0000 0.9905

Constrained -15811.447 23.435 0.0100 0.0095

*DPA1* Unconstrained -37311.148 0.000 1.0000 0.9301

Constrained -37326.618 15.470 0.0729 0.0698

*FAS1* Unconstrained -37716.118 0.000 1.0000 0.7919

Constrained -37724.921 8.803 0.2112 0.2081

*KU70* Unconstrained -17711.670 0.000 1.0000 0.8810

Constrained -17720.333 8.663 0.1190 0.1190

*LCB1* Unconstrained -12920.973 0.000 1.0000 0.7756

Constrained -12925.657 4.684 0.2345 0.2244

*LCB2* Unconstrained -16091.220 0.000 1.0000 0.9255

Constrained -16101.101 9.881 0.0917 0.0745

*TEF1* Unconstrained -5526.199 0.000 1.0000 0.9682

Constrained -5554.943 28.743 0.0372 0.0318

*TOP1* Unconstrained -22381.434 0.000 1.0000 0.7870

Constrained -22388.103 6.668 0.2280 0.2130

*TUB2* Unconstrained -22381.434 0.000 1.0000 0.7870

Constrained -22388.103 6.668 0.2280 0.2130

**Table C:** Results of Shimodaira-Hasegawa (SH) and Approximately Unbiased (AU) tests as implemented in IQ-Tree [3]. In these tests, the topology of trees inferred from the alignment of 20 housekeeping genes was constrained to include a supported branch (BS > 70%) that occurred in single-gene trees. The positions of the branches listed in the Branch (left) column of the table are indicated by labeled arrows in the corresponding trees shown in **Fig B**. Sequences were aligned using Muscle as implemented in MEGA7 [4], and alignments were concatenated using SequenceMatrix [5]. Concatenated sequences in the alignments used for this analysis were partitioned by gene to allow the most appropriate substitution model for each partition to be used [6].

**Branch logL deltaL p-SH p-AU**

Unconstrained -444797.446 0.000 1.0000 0.9780

CPR1-A -444942.081 144.635 0.0014 0.0008

CPR1-B -444850.109 52.663 0.1888 0.0467

DPA1-A -444832.735 35.289 0.3340 0.0244

DPA1-B -444848.071 50.625 0.1901 0.0104

FAS1-A -444832.741 35.290 0.3804 0.0240

KU70-A -444877.461 80.010 0.0628 0.0015

LCB1-A -445167.530 370.079 0.0000 0.0000

LCB2-A -444832.735 35.289 0.3340 0.0244

LCB2-B -444836.514 39.068 0.2881 0.0983

TEF1-A -444939.411 141.966 0.0020 0.0013

TEF1-B -444847.169 49.724 0.2119 0.0558

TOP1-A -444864.106 66.655 0.1312 0.0107

TUB2-A -444850.125 52.674 0.2119 0.0386

**Fig A:** Trees inferred from concatenated sequences of the housekeeping genes listed in **Table A**. Sequences were aligned using Muscle as implemented in MEGA7 [4], and alignments were concatenated using SequenceMatrix [5]. Trees were inferred using maximum likelihood with an ultrafast bootstrap method [7] as implemented in IQ-Tree [3]. Concatenated sequences in the alignments used for this analysis were partitioned to allow the most appropriate substitution model for each partition to be used [6]. All trees are rooted on *Microcyclospora tardicrescens*. Numbers near branches or under arrow labels are bootstrap values (BS) based on 1000 pseudoreplicates; only values > 70 are shown.

**20-gene tree**

***Cordyceps confragosa UM487***

***Cordyceps confragosa 1005***

***Beauveria bassiana***

***Trichoderma arundinaceum***

***Trichoderma brevicompactum***

***Stachybotrys chartarum 40288***

***Stachybotrys chartarum 40293***

***Stachybotrys chartarum 7711***

***Stachybotrys chlorohalonata***

***Myrothecium roridum***

***Fusarium graminearum***

***Fusarium sporotrichioides***

***Fusarium longipes***

***FIESC 12***

***Spicellum ovalisporum***

***Spicellum roseum***

***Trichothecium roseum 195227***

***Trichothecium roseum 197141***

***Trichothecium roseum K7-1***

***Microcyclospora tardicrescens***

**100**

**100**

**100**

**100**

**100**

**100**

**100**

**100**

**100**

**92**

**100**

**100**

**100**

**97**

**100**

**98**

0.1

**11-gene tree**

***Cordyceps confragosa UM487***

***Cordyceps confragosa 1005***

***Beauveria bassiana***

***Trichoderma arundinaceum***

***Trichoderma brevicompactum***

***Stachybotrys chartarum 40288***

***Stachybotrys chartarum 40293***

***Stachybotrys chartarum 7711***

***Stachybotrys chlorohalonata***

***Myrothecium roridum***

***Fusarium graminearum***

***Fusarium sporotrichioides***

***Fusarium longipes***

***FIESC 12***

***Spicellum ovalisporum***

***Spicellum roseum***

***Trichothecium roseum 195227***

***Trichothecium roseum 197141***

***Trichothecium roseum K7-1***

***Microcyclospora tardicrescens***

**100**

**100**

**100**

**100**

**100**

**100**

**100**

**100**

**100**

**87**

**100**

**100**

**100**

**87**

**100**

**88**

0.1

**16-gene tree**

***Cordyceps confragosa UM487***

***Cordyceps confragosa 1005***

***Beauveria bassiana***

***Trichoderma arundinaceum***

***Trichoderma brevicompactum***

***Stachybotrys chartarum 40288***

***Stachybotrys chartarum 40293***

***Stachybotrys chartarum 7711***

***Stachybotrys chlorohalonata***

***Myrothecium roridum***

***Fusarium graminearum***

***Fusarium sporotrichioides***

***Fusarium longipes***

***FIESC 12***

***Spicellum ovalisporum***

***Spicellum roseum***

***Trichothecium roseum 195227***

***Trichothecium roseum 197141***

***Trichothecium roseum K7-1***

***Microcyclospora tardicrescens***

**100**

**100**

**100**

**100**

**100**

**100**

**100**

**100**

**100**

**97**

**100**

**100**

**100**

**99**

**100**

**99**

0.1

**Fig B:** Single-gene trees inferred from the housekeeping genes listed in **Table A**. Sequences were aligned using Muscle as implemented in MEGA7 [4]. Trees were inferred using the maximum likelihood method with ultrafast bootstrap [7] as implemented in IQ-Tree [3]. Branches that conflicted with the 20-gene tree (i.e., species phylogeny) shown in **Fig A**are indicated with thick arrows. The arrow labels are the same as the branch designations listed in **Table C**. Numbers near branches or under arrow labels are bootstrap values (BS) based on 1000 pseudoreplicates; only values > 70 are shown. Note that for ease of reading, narrow blue lines point to BS values for some branches. These narrow blue lines do not indicate branches that conflict with the species phylogeny. All trees are rooted on *Microcyclospora tardicrescens*.

**
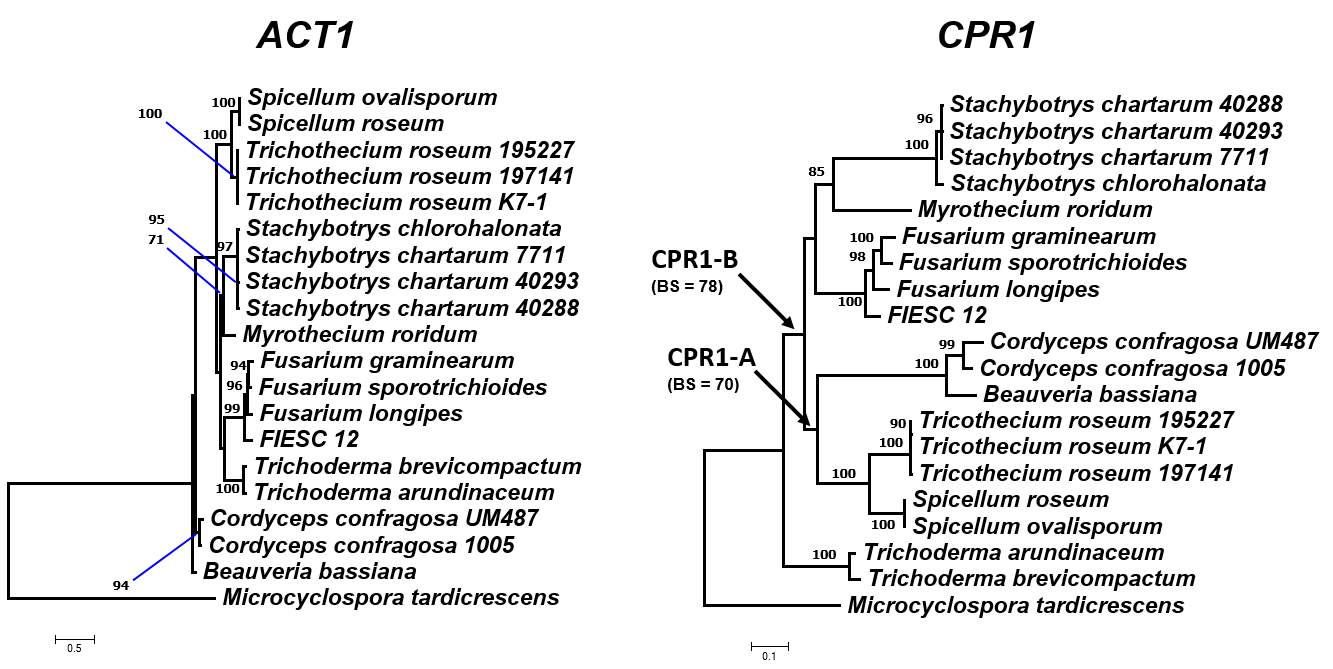
**

**
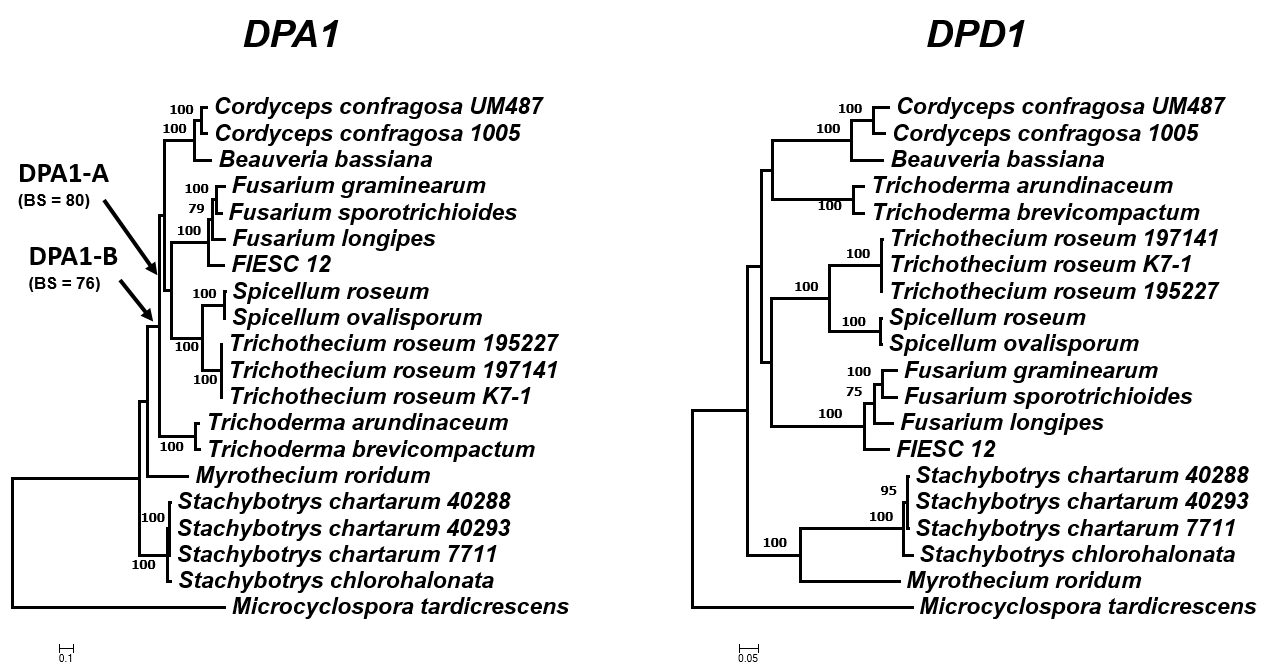
**

**Fig B** (continued)


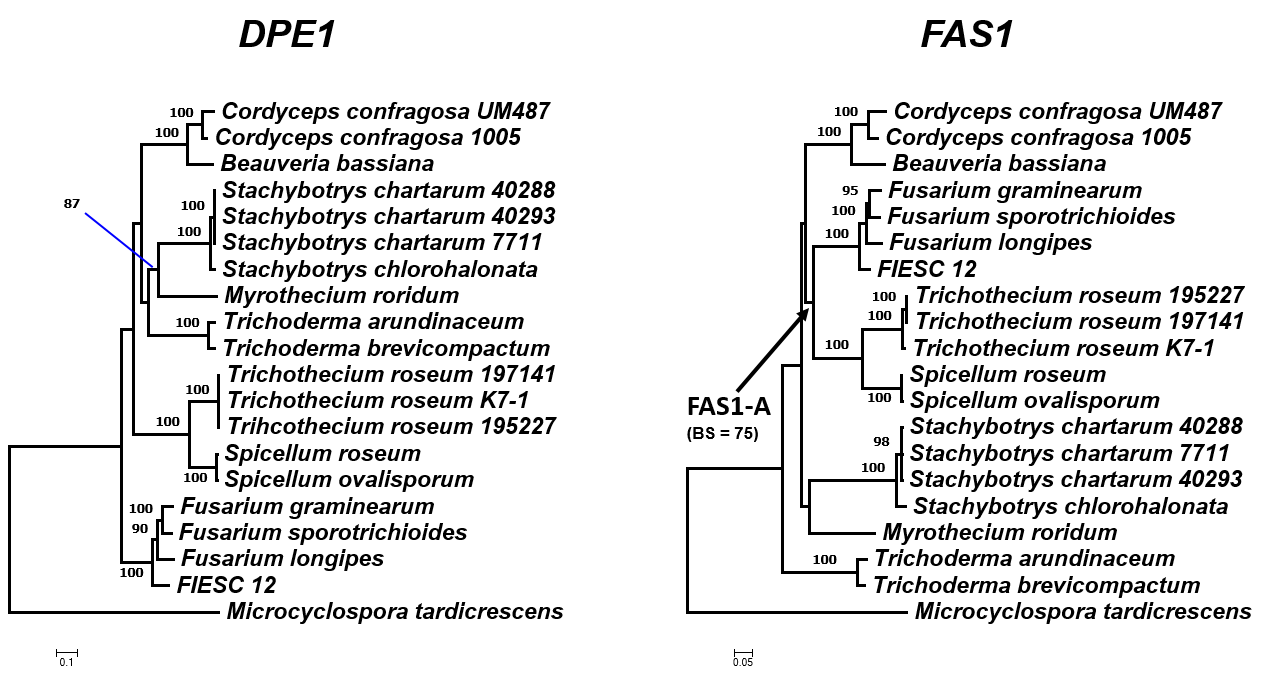


**
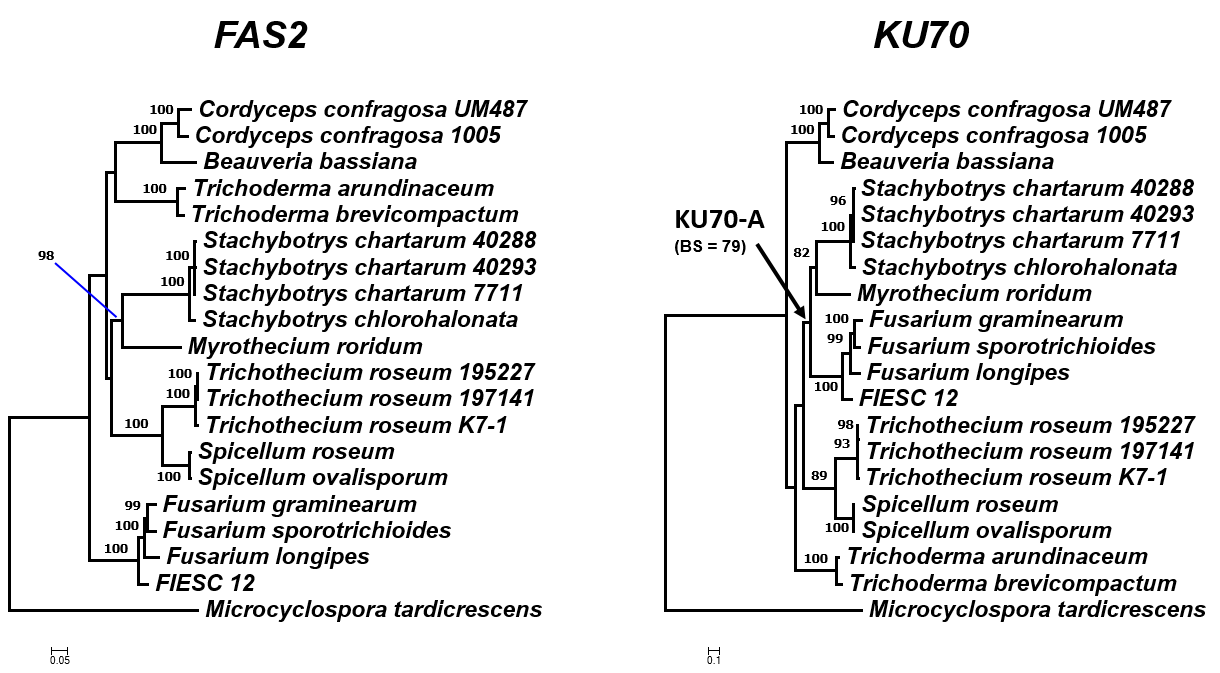
**

**Fig B** (continued)


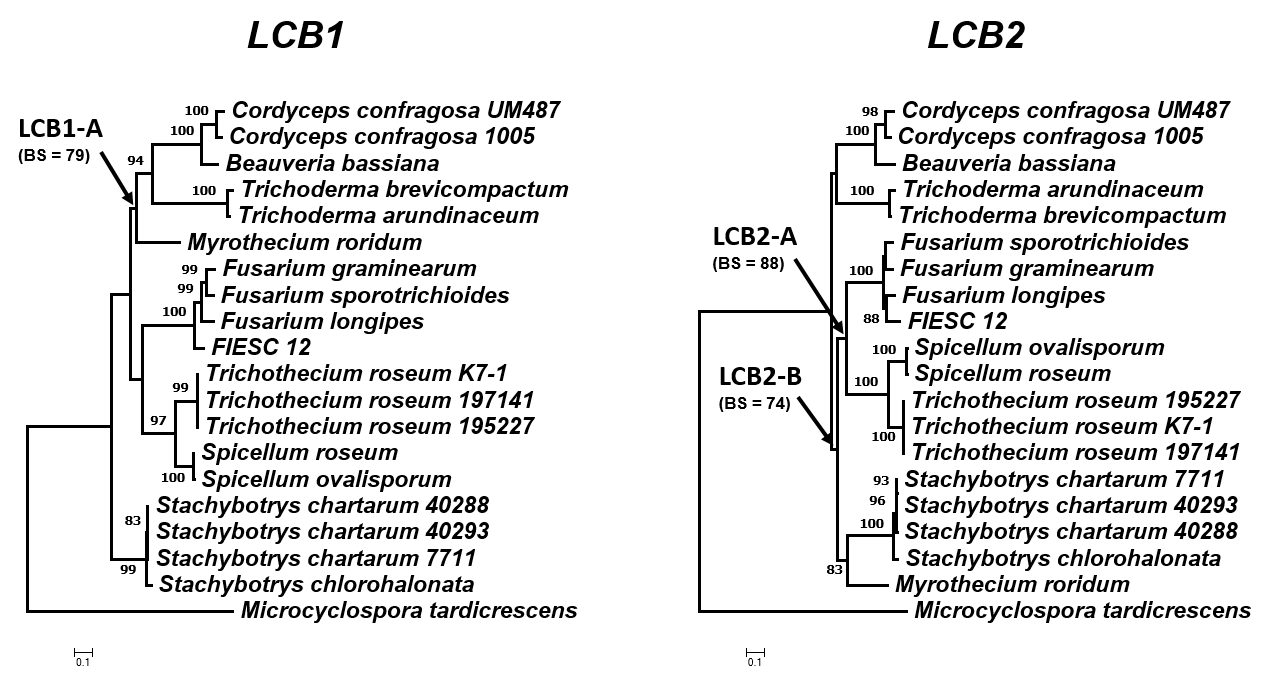


**
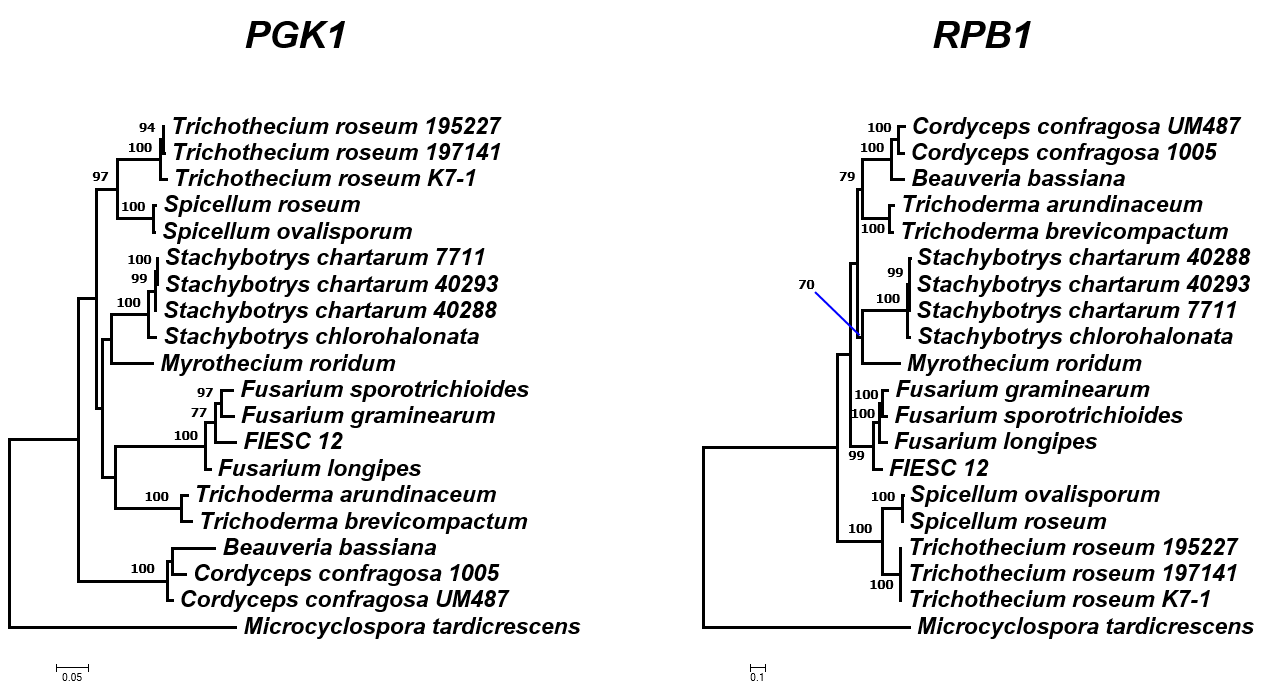
**

**Fig B** (continued)


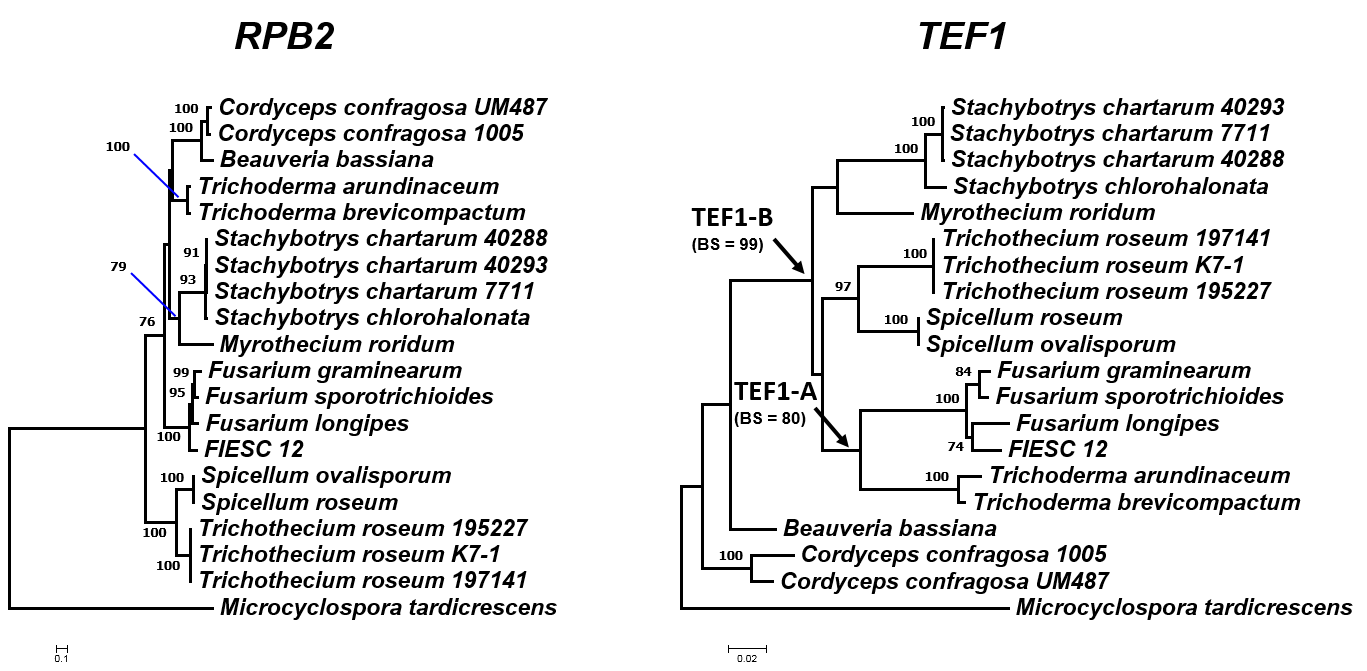


**
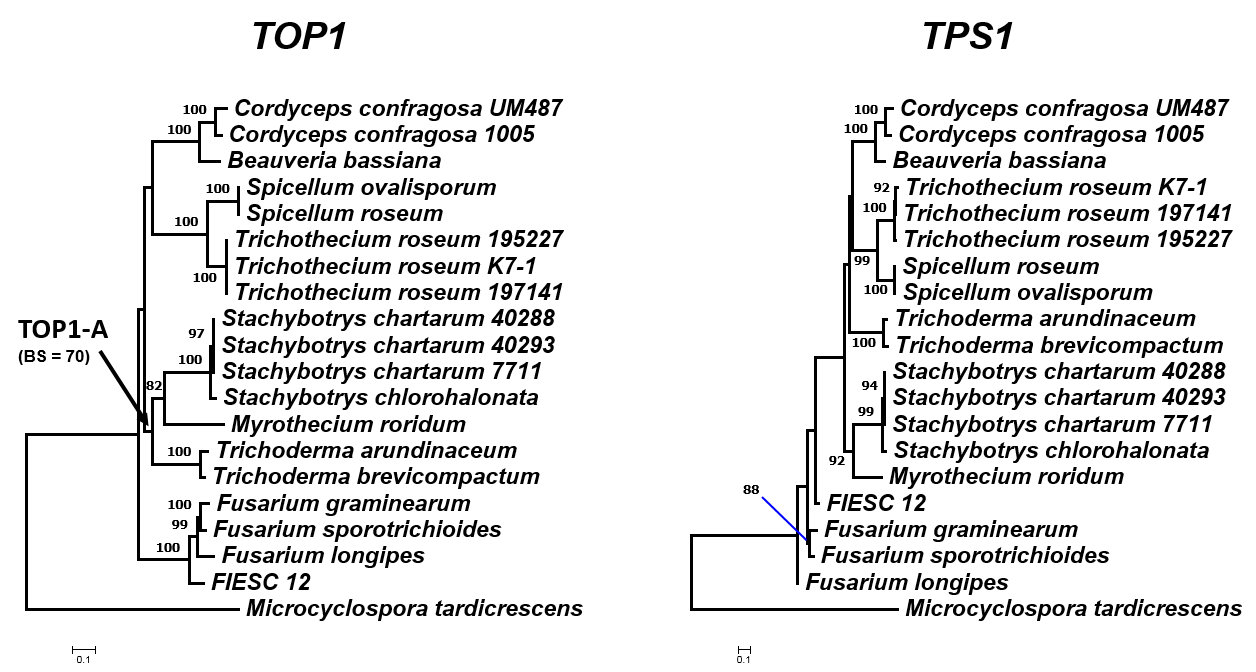
**

**Fig B** (continued)

**
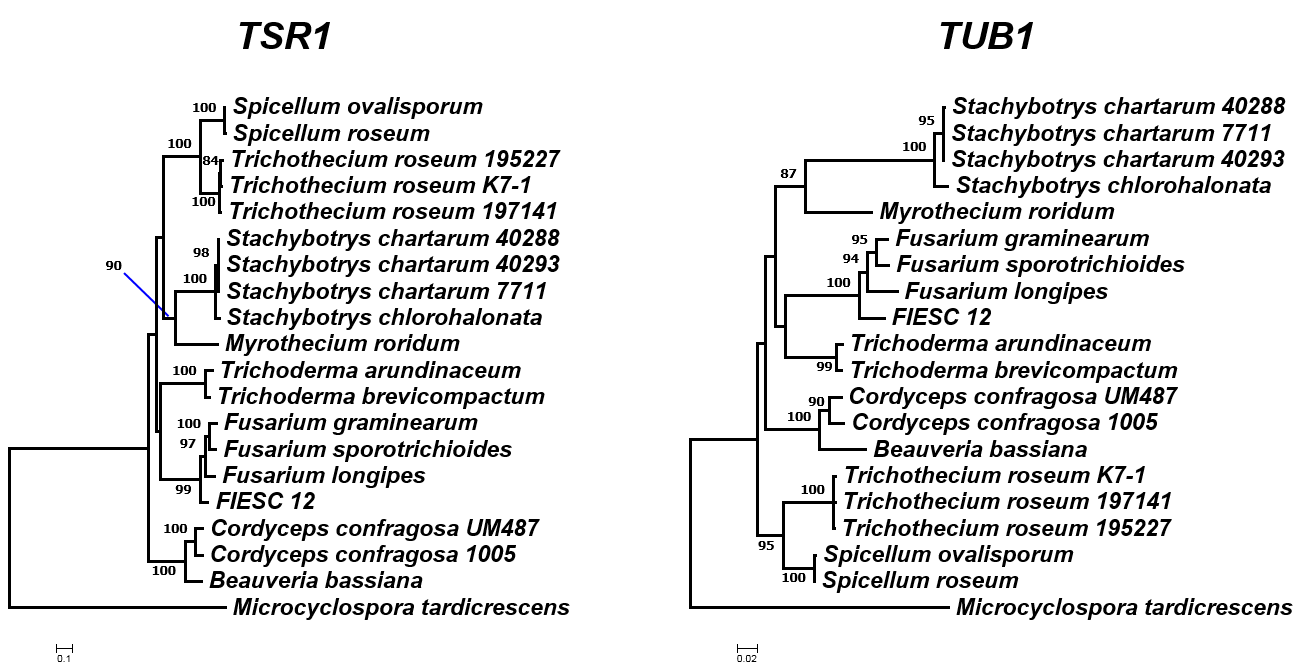
**

**
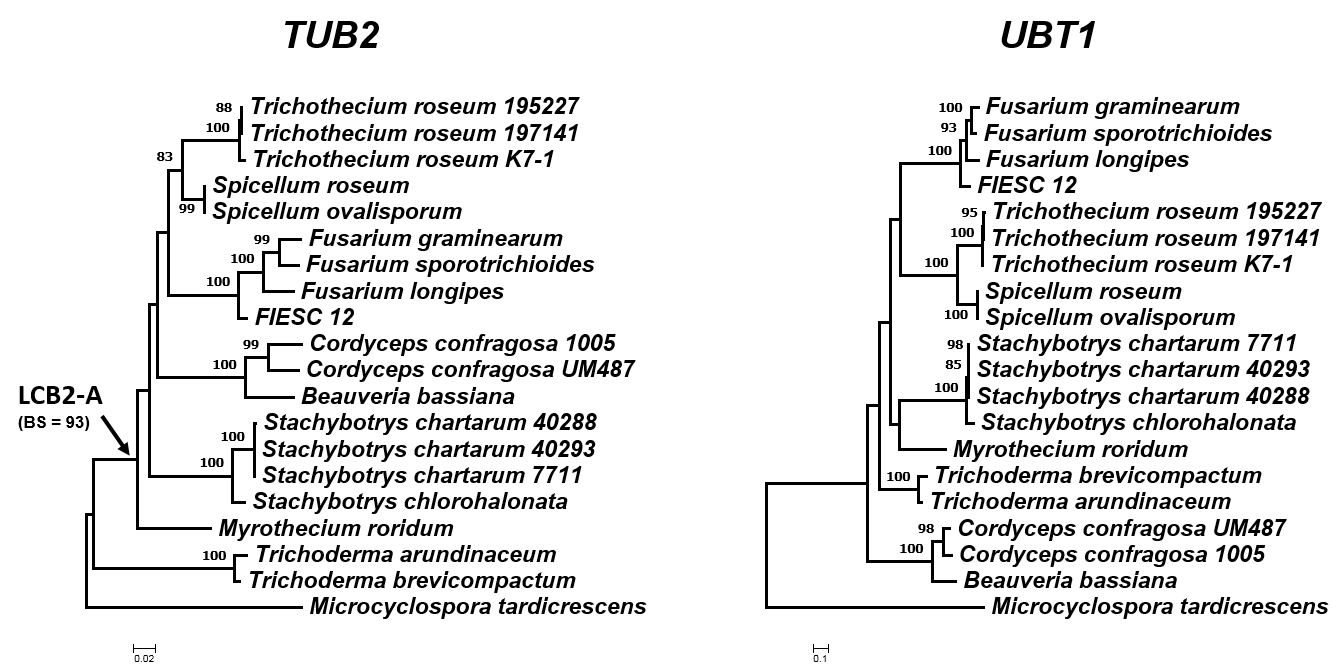
**

**References cited in S3 File**

1. Shimodaira H, Hasagawa M (1999) Multiple comparisons of log-likelihoods with applications to phylogenetic inference. Mol Biol Evol 16: 1114-1116.

2. Shimodaira H (2002) An approximately unbiased test of phylogenetic tree selection. Syst Biol 51: 492-508.

3. Nguyen L-T, Schmidt HA, von Haeseler A, Minh BQ (2014) IQ-TREE: A fast and effective stochastic algorithm for estimating maximum likelihood phylogenies. Mol Biol Evol 32: 268–274.

4. Kumar S, Stecher G, Tamura K (2016) MEGA7: Molecular Evolutionary Genetics Analysis version 7.0 for bigger datasets. Mol Biol Evol 33: 1870-1874.

5. Vaidya G, Lohman DJ, Meier R (2011) SequenceMatrix: concatenation software for the fast assembly of multi-gene datasets with character set and codon information. Cladistics 27: 171-180.

6. Chernomor O, von Haeseler A, Minh BQ (2016) Terrace Aware Data Structure for Phylogenomic Inference from Supermatrices. Syst Biol 65: 997-1008.

7. Minh BQ, Nguyen MA, von Haeseler A (2013) Ultrafast approximation for phylogenetic bootstrap. Mol Biol Evol 30: 1188-1195.
