# Supplementary material for: Evolution of structural diversity of trichothecenes, a family of toxins produced by plant pathogenic and entomopathogenic fungi
Source: PLoS Pathog. 2018 Apr 12;14(4):e1006946. doi: 10.1371/journal.ppat.1006946 (PMC5897003; doi:10.1371/journal.ppat.1006946)
Supplement: S1 Fig — (DOCX) [file ppat.1006946.s004.docx]

**S1 Figure: Phylogenetic analysis and rational for designation of *TRI22* as a distinct gene from *TRI11*.**


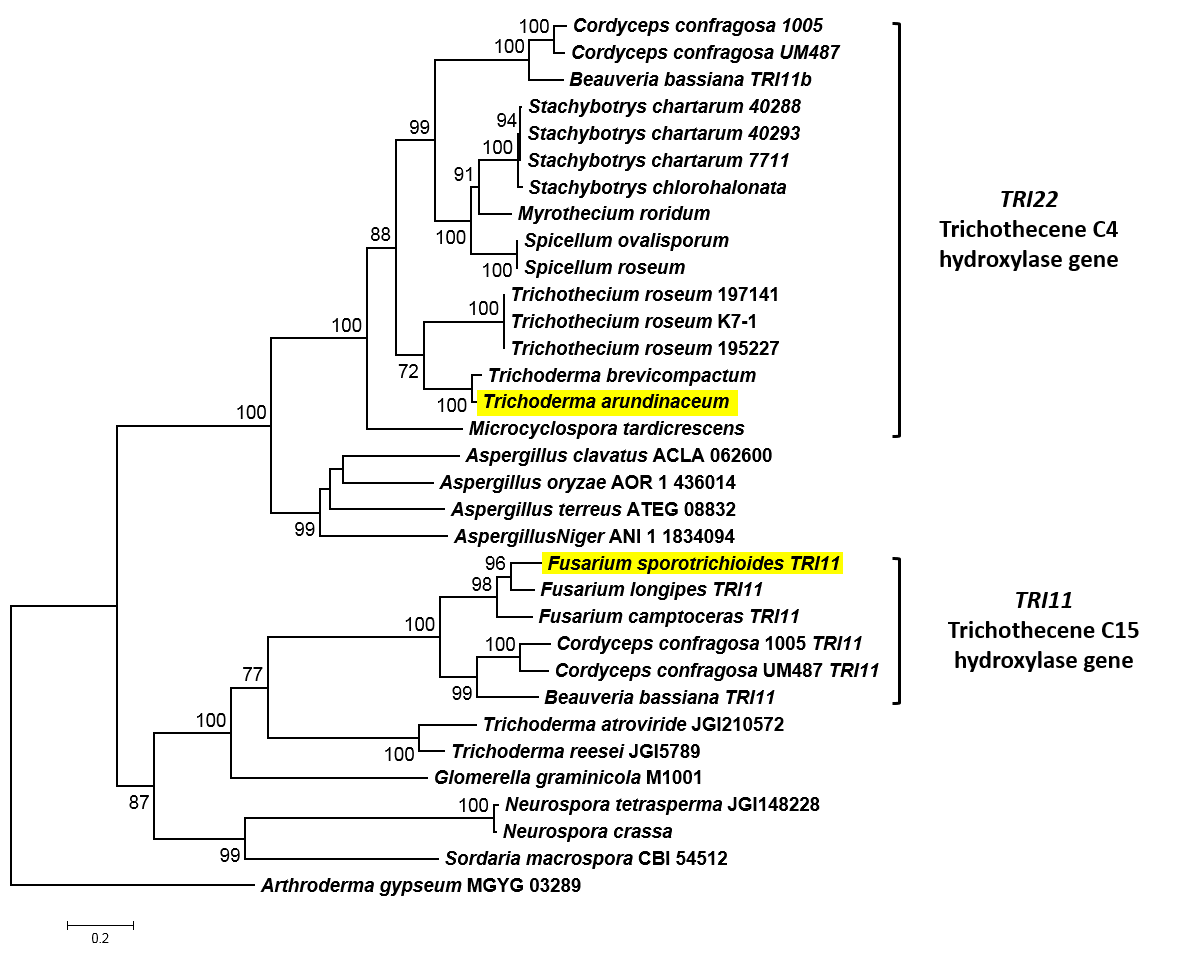


The functions of the *TRI11* gene in trichothecene biosynthesis has been determined previously in *Fusarium sporotrichioides* and *Trichoderma arundinaceum* (Alexander et al., 1998; Cardoza et al., 2011). The *Fusarium* *TRI11*-encoded enzyme (Tri11) is responsible for trichothecene hydroxylation at carbon atom 15 (C15), whereas the *Trichoderma* *TRI11*-encoded enzyme is responsible for trichothecene hydroxylation at C4. The predicted amino acid sequences of the two proteins exhibit only 37% identity. In the current study, to clarify relationships among *TRI11* homologs we conducted a phylogenetic analysis of *TRI11* homologs from trichothecene-producing fungi and *TRI11*-like homologs from trichothecene-nonproducing fungi. In the analysis, the predicted amino acid sequences of Tri11 and Tri11-like proteins were aligned using the Muscle alignment function in MEGA7 (Kumar et al., 2016), and the resulting alignment was subjected to maximum likelihood analysis using the program IQ-Tree (Nguyen et al., 2014). In the resulting phylogenetic tree, the *TRI11* homologs grouped into two well-supported clades. One clade included the C15 hydroxylase *TRI11* from *F. sporotrichioides* (highlighted in yellow), and the other clade included the C4 hydroxylase *TRI11* from *T. arundinaceum* (highlighted in yellow). The two *TRI11* clades were more distantly related to one another than they were to *TRI11*-like homologs from trichothecene-nonproducing fungi. Given the distant relationship of the *TRI11* clades, the low level of amino acid sequence identity of homologs from different clades, and the evidence for differences in function of homologs in the different clades, we propose that the two clades represent distinct genes and as such should have different gene designations. Because, the *Fusarium* homolog responsible for trichothecene C15 hydroxylation was described before the *Trichoderma* homolog responsible for C4 hydroxylation, we propose that the *Fusarium* homologs retain the *TRI11* gene designation, and the *Trichoderma* homologs be given the novel gene designation *TRI22*.

**References**

Alexander, N. J., Hohn, T. M., McCormick, S. P., 1998. The *TRI11* gene of *Fusarium sporotrichioides* encodes a cytochrome P450 monooxygenase required for C-15 hydroxylation in trichothecene biosynthesis. Appl.Environ.Microbiol. 64, 221-225.

Cardoza, R. E., Malmierca, M. G., Hermosa, M. R., Alexander, N. J., McCormick, S. P., Proctor, R. H., Tijerino, A. M., Rumbero, A., Monte, E., Gutiérrez, S., 2011. Identification of Loci and Functional Characterization of Trichothecene Biosynthesis Genes in Filamentous Fungi of the Genus *Trichoderma*. Applied and Environmental Microbiology 77, 4867-4877.

Kumar, S., Stecher, G., Tamura, K., 2016. MEGA7: Molecular Evolutionary Genetics Analysis version 7.0 for bigger datasets. Mol. Biol. Evol.

Nguyen, L.-T., Schmidt, H. A., von Haeseler, A., Minh, B. Q., 2014. IQ-TREE: A fast and effective stochastic algorithm for estimating maximum likelihood phylogenies. Mol. Biol. Evol. 32, 268–274.
