# Supplementary material for: Evolution of structural diversity of trichothecenes, a family of toxins produced by plant pathogenic and entomopathogenic fungi
Source: PLoS Pathog. 2018 Apr 12;14(4):e1006946. doi: 10.1371/journal.ppat.1006946 (PMC5897003; doi:10.1371/journal.ppat.1006946)
Supplement: S2 Fig — In the diagrams (above), green arrows represent known TRI genes, and gray arrows represent genes that are unique to a region in a particular genus. Numbers below arrows are locus tag numbers (with five-letter prefix, the underscore, and in some cases a zero omitted). The tables below the diagrams include predicted functions of genes based on Blast2Go analysis and supplemented with manual blast analysis in some cases. Tables also include information on contigs on which the genes occur. In the tables, genes above and below a double line are on different contigs. A. Beauveria bassiana; B. Cordyceps confragosa—orange arrows represent genes that are common to the TRI cluster locus in Beauveria and Cordyceps; C. Microcylospora tardicrescens; D. Myrothecium roridum—purple arrows represent genes that are common to TRI loci in Myrothecium and Stachybotrys chartarum; E. Spicellum ovalisporum; F. Spicellum roseum; G. Stachybotrys chartarum; H. Trichoderma arundinaceum; and I. Trichothecium roseum. (PPTX) [file ppat.1006946.s005.pptx]

## Slide 1
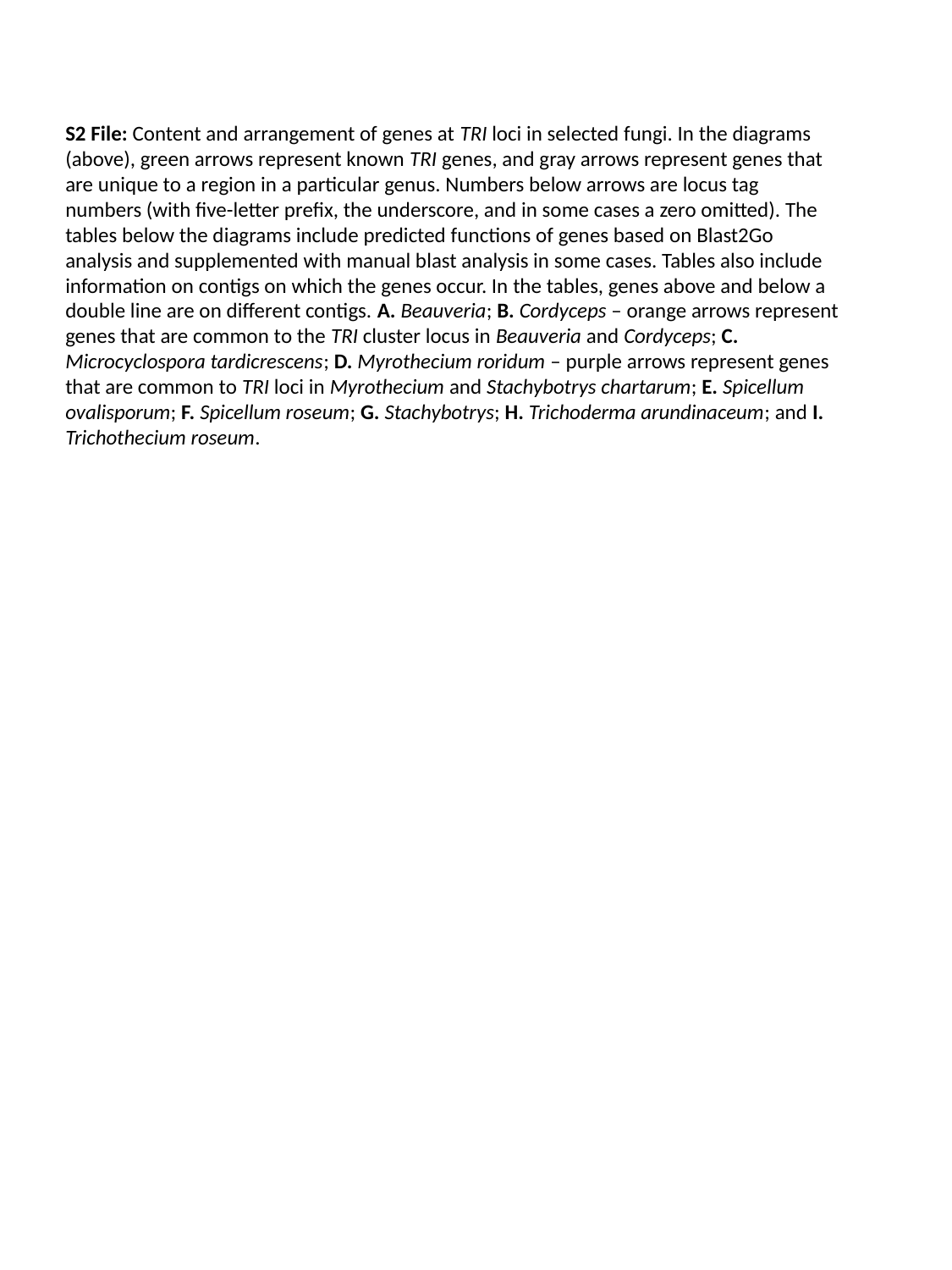

S2 File: Content and arrangement of genes at TRI loci in selected fungi. In the diagrams (above), green arrows represent known TRI genes, and gray arrows represent genes that are unique to a region in a particular genus. Numbers below arrows are locus tag numbers (with five-letter prefix, the underscore, and in some cases a zero omitted). The tables below the diagrams include predicted functions of genes based on Blast2Go analysis and supplemented with manual blast analysis in some cases. Tables also include information on contigs on which the genes occur. In the tables, genes above and below a double line are on different contigs. A. Beauveria; B. Cordyceps – orange arrows represent genes that are common to the TRI cluster locus in Beauveria and Cordyceps; C. Microcyclospora tardicrescens; D. Myrothecium roridum – purple arrows represent genes that are common to TRI loci in Myrothecium and Stachybotrys chartarum; E. Spicellum ovalisporum; F. Spicellum roseum; G. Stachybotrys; H. Trichoderma arundinaceum; and I. Trichothecium roseum.

## Slide 2
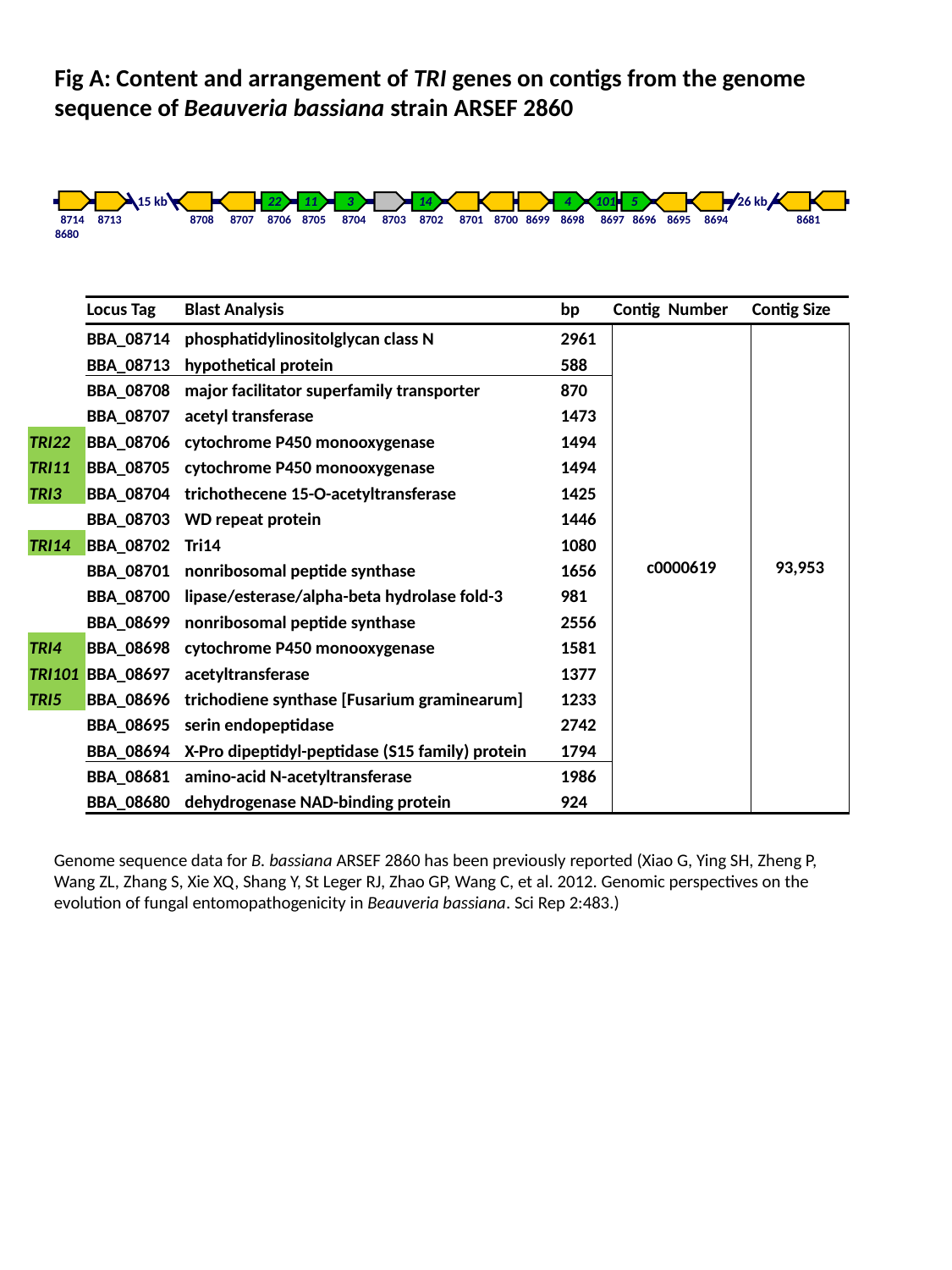

Fig A: Content and arrangement of TRI genes on contigs from the genome sequence of Beauveria bassiana strain ARSEF 2860
15 kb
 22
 11
3
14
4
 101
5
26 kb
 8714 8713 8708 8707 8706 8705 8704 8703 8702 8701 8700 8699 8698 8697 8696 8695 8694 8681 8680
| | Locus Tag | Blast Analysis | bp | Contig Number | Contig Size |
| --- | --- | --- | --- | --- | --- |
| | BBA\_08714 | phosphatidylinositolglycan class N | 2961 | c0000619 | 93,953 |
| | BBA\_08713 | hypothetical protein | 588 | | |
| | BBA\_08708 | major facilitator superfamily transporter | 870 | | |
| | BBA\_08707 | acetyl transferase | 1473 | | |
| TRI22 | BBA\_08706 | cytochrome P450 monooxygenase | 1494 | | |
| TRI11 | BBA\_08705 | cytochrome P450 monooxygenase | 1494 | | |
| TRI3 | BBA\_08704 | trichothecene 15-O-acetyltransferase | 1425 | | |
| | BBA\_08703 | WD repeat protein | 1446 | | |
| TRI14 | BBA\_08702 | Tri14 | 1080 | | |
| | BBA\_08701 | nonribosomal peptide synthase | 1656 | | |
| | BBA\_08700 | lipase/esterase/alpha-beta hydrolase fold-3 | 981 | | |
| | BBA\_08699 | nonribosomal peptide synthase | 2556 | | |
| TRI4 | BBA\_08698 | cytochrome P450 monooxygenase | 1581 | | |
| TRI101 | BBA\_08697 | acetyltransferase | 1377 | | |
| TRI5 | BBA\_08696 | trichodiene synthase [Fusarium graminearum] | 1233 | | |
| | BBA\_08695 | serin endopeptidase | 2742 | | |
| | BBA\_08694 | X-Pro dipeptidyl-peptidase (S15 family) protein | 1794 | | |
| | BBA\_08681 | amino-acid N-acetyltransferase | 1986 | | |
| | BBA\_08680 | dehydrogenase NAD-binding protein | 924 | | |
Genome sequence data for B. bassiana ARSEF 2860 has been previously reported (Xiao G, Ying SH, Zheng P, Wang ZL, Zhang S, Xie XQ, Shang Y, St Leger RJ, Zhao GP, Wang C, et al. 2012. Genomic perspectives on the evolution of fungal entomopathogenicity in Beauveria bassiana. Sci Rep 2:483.)

## Slide 3
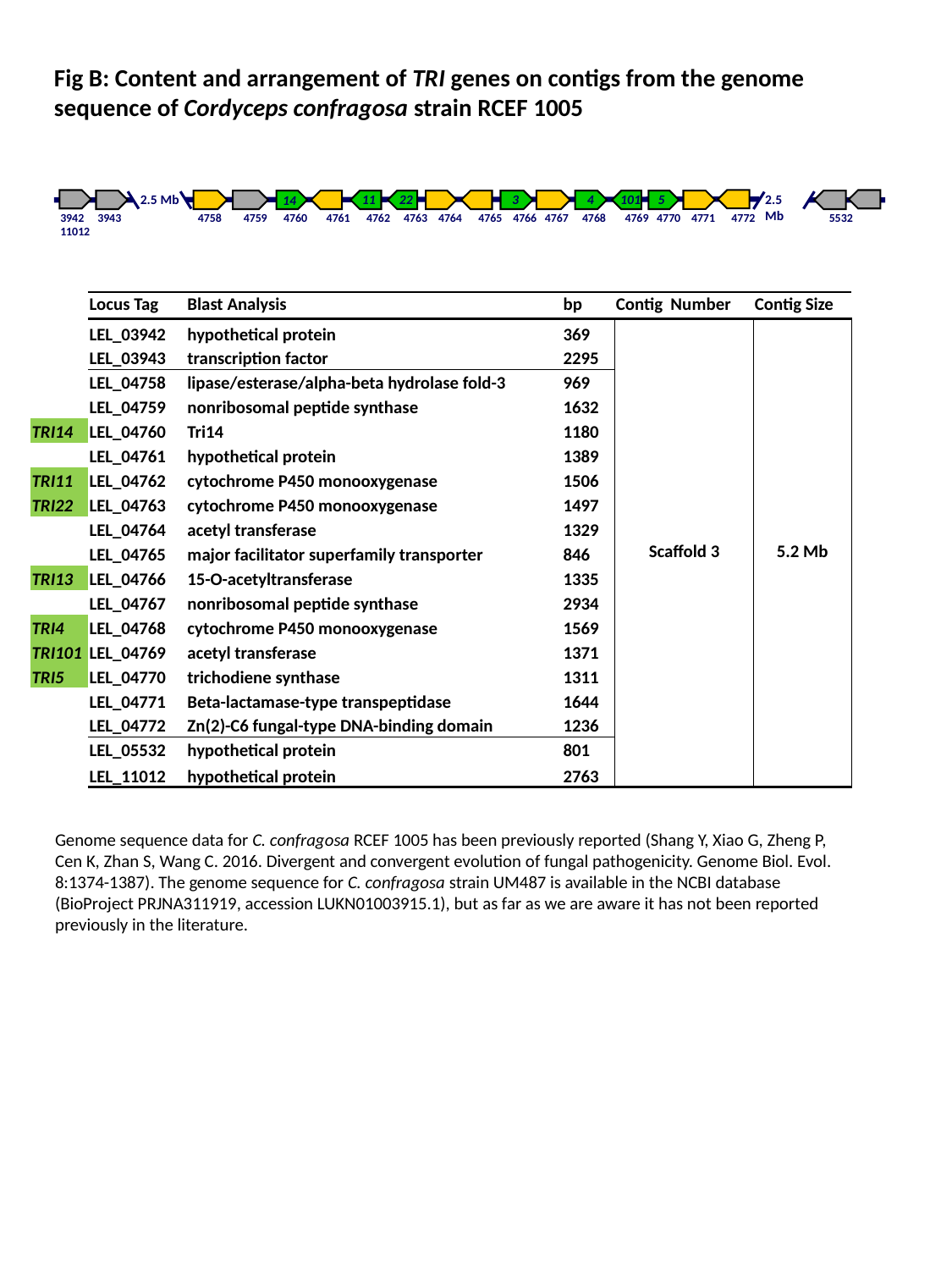

Fig B: Content and arrangement of TRI genes on contigs from the genome sequence of Cordyceps confragosa strain RCEF 1005
 11
 22
3
4
 101
5
2.5 Mb
2.5 Mb
14
3942 3943 4758 4759 4760 4761 4762 4763 4764 4765 4766 4767 4768 4769 4770 4771 4772 5532 11012
| | Locus Tag | Blast Analysis | bp | Contig Number | Contig Size |
| --- | --- | --- | --- | --- | --- |
| | LEL\_03942 | hypothetical protein | 369 | Scaffold 3 | 5.2 Mb |
| | LEL\_03943 | transcription factor | 2295 | | |
| | LEL\_04758 | lipase/esterase/alpha-beta hydrolase fold-3 | 969 | | |
| | LEL\_04759 | nonribosomal peptide synthase | 1632 | | |
| TRI14 | LEL\_04760 | Tri14 | 1180 | | |
| | LEL\_04761 | hypothetical protein | 1389 | | |
| TRI11 | LEL\_04762 | cytochrome P450 monooxygenase | 1506 | | |
| TRI22 | LEL\_04763 | cytochrome P450 monooxygenase | 1497 | | |
| | LEL\_04764 | acetyl transferase | 1329 | | |
| | LEL\_04765 | major facilitator superfamily transporter | 846 | | |
| TRI13 | LEL\_04766 | 15-O-acetyltransferase | 1335 | | |
| | LEL\_04767 | nonribosomal peptide synthase | 2934 | | |
| TRI4 | LEL\_04768 | cytochrome P450 monooxygenase | 1569 | | |
| TRI101 | LEL\_04769 | acetyl transferase | 1371 | | |
| TRI5 | LEL\_04770 | trichodiene synthase | 1311 | | |
| | LEL\_04771 | Beta-lactamase-type transpeptidase | 1644 | | |
| | LEL\_04772 | Zn(2)-C6 fungal-type DNA-binding domain | 1236 | | |
| | LEL\_05532 | hypothetical protein | 801 | | |
| | LEL\_11012 | hypothetical protein | 2763 | | |
Genome sequence data for C. confragosa RCEF 1005 has been previously reported (Shang Y, Xiao G, Zheng P, Cen K, Zhan S, Wang C. 2016. Divergent and convergent evolution of fungal pathogenicity. Genome Biol. Evol. 8:1374-1387). The genome sequence for C. confragosa strain UM487 is available in the NCBI database (BioProject PRJNA311919, accession LUKN01003915.1), but as far as we are aware it has not been reported previously in the literature.

## Slide 4
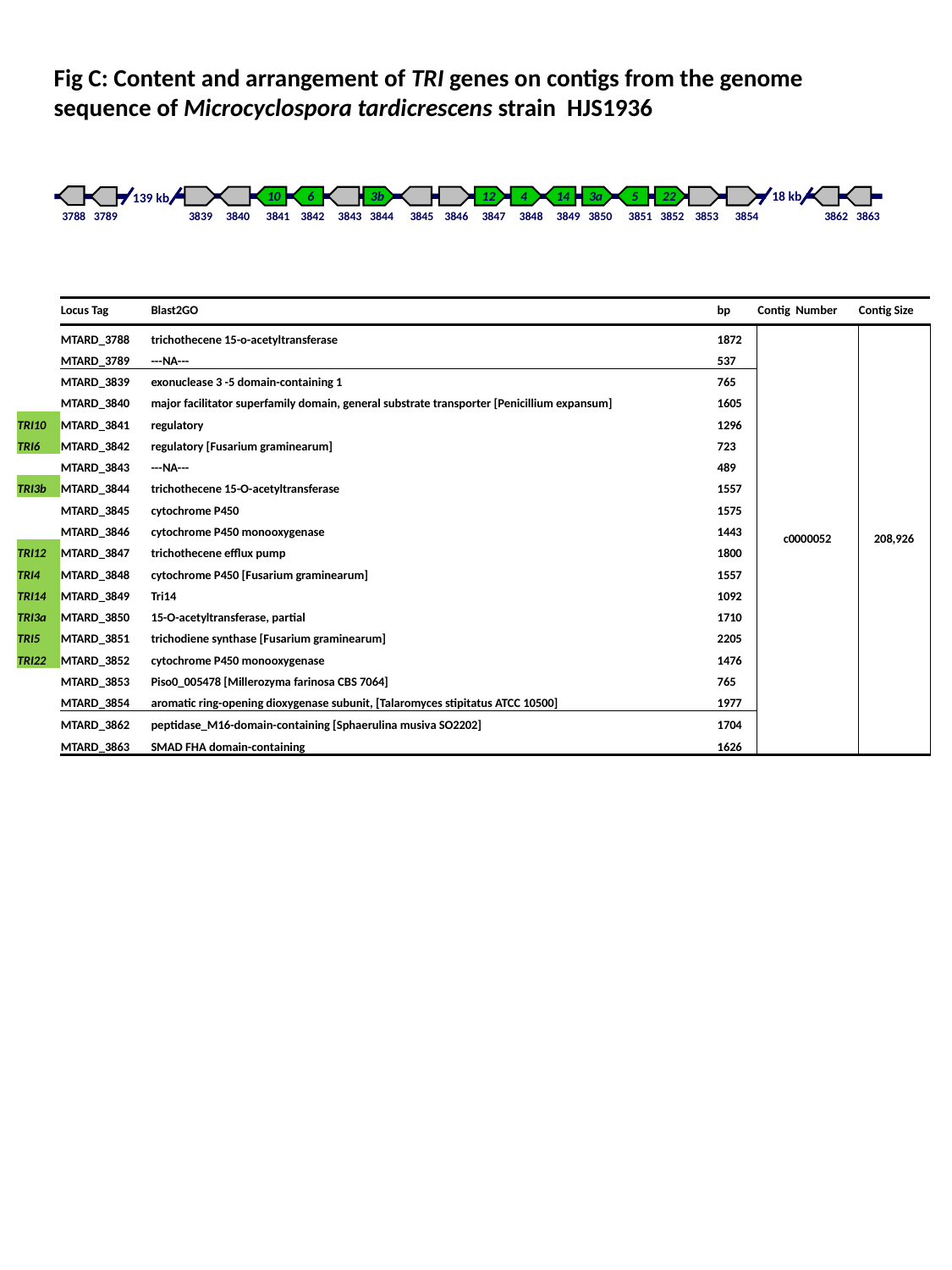

Fig C: Content and arrangement of TRI genes on contigs from the genome sequence of Microcyclospora tardicrescens strain HJS1936
 3a
18 kb
10
6
12
4
5
3b
14
22
139 kb
 3788 3789 3839 3840 3841 3842 3843 3844 3845 3846 3847 3848 3849 3850 3851 3852 3853 3854 3862 3863
| | Locus Tag | Blast2GO | bp | | Contig Number | Contig Size |
| --- | --- | --- | --- | --- | --- | --- |
| | MTARD\_3788 | trichothecene 15-o-acetyltransferase | 1872 | | c0000052 | 208,926 |
| | MTARD\_3789 | ---NA--- | 537 | | | |
| | MTARD\_3839 | exonuclease 3 -5 domain-containing 1 | 765 | | | |
| | MTARD\_3840 | major facilitator superfamily domain, general substrate transporter [Penicillium expansum] | 1605 | | | |
| TRI10 | MTARD\_3841 | regulatory | 1296 | | | |
| TRI6 | MTARD\_3842 | regulatory [Fusarium graminearum] | 723 | | | |
| | MTARD\_3843 | ---NA--- | 489 | | | |
| TRI3b | MTARD\_3844 | trichothecene 15-O-acetyltransferase | 1557 | | | |
| | MTARD\_3845 | cytochrome P450 | 1575 | | | |
| | MTARD\_3846 | cytochrome P450 monooxygenase | 1443 | | | |
| TRI12 | MTARD\_3847 | trichothecene efflux pump | 1800 | | | |
| TRI4 | MTARD\_3848 | cytochrome P450 [Fusarium graminearum] | 1557 | | | |
| TRI14 | MTARD\_3849 | Tri14 | 1092 | | | |
| TRI3a | MTARD\_3850 | 15-O-acetyltransferase, partial | 1710 | | | |
| TRI5 | MTARD\_3851 | trichodiene synthase [Fusarium graminearum] | 2205 | | | |
| TRI22 | MTARD\_3852 | cytochrome P450 monooxygenase | 1476 | | | |
| | MTARD\_3853 | Piso0\_005478 [Millerozyma farinosa CBS 7064] | 765 | | | |
| | MTARD\_3854 | aromatic ring-opening dioxygenase subunit, [Talaromyces stipitatus ATCC 10500] | 1977 | | | |
| | MTARD\_3862 | peptidase\_M16-domain-containing [Sphaerulina musiva SO2202] | 1704 | | | |
| | MTARD\_3863 | SMAD FHA domain-containing | 1626 | | | |

## Slide 5
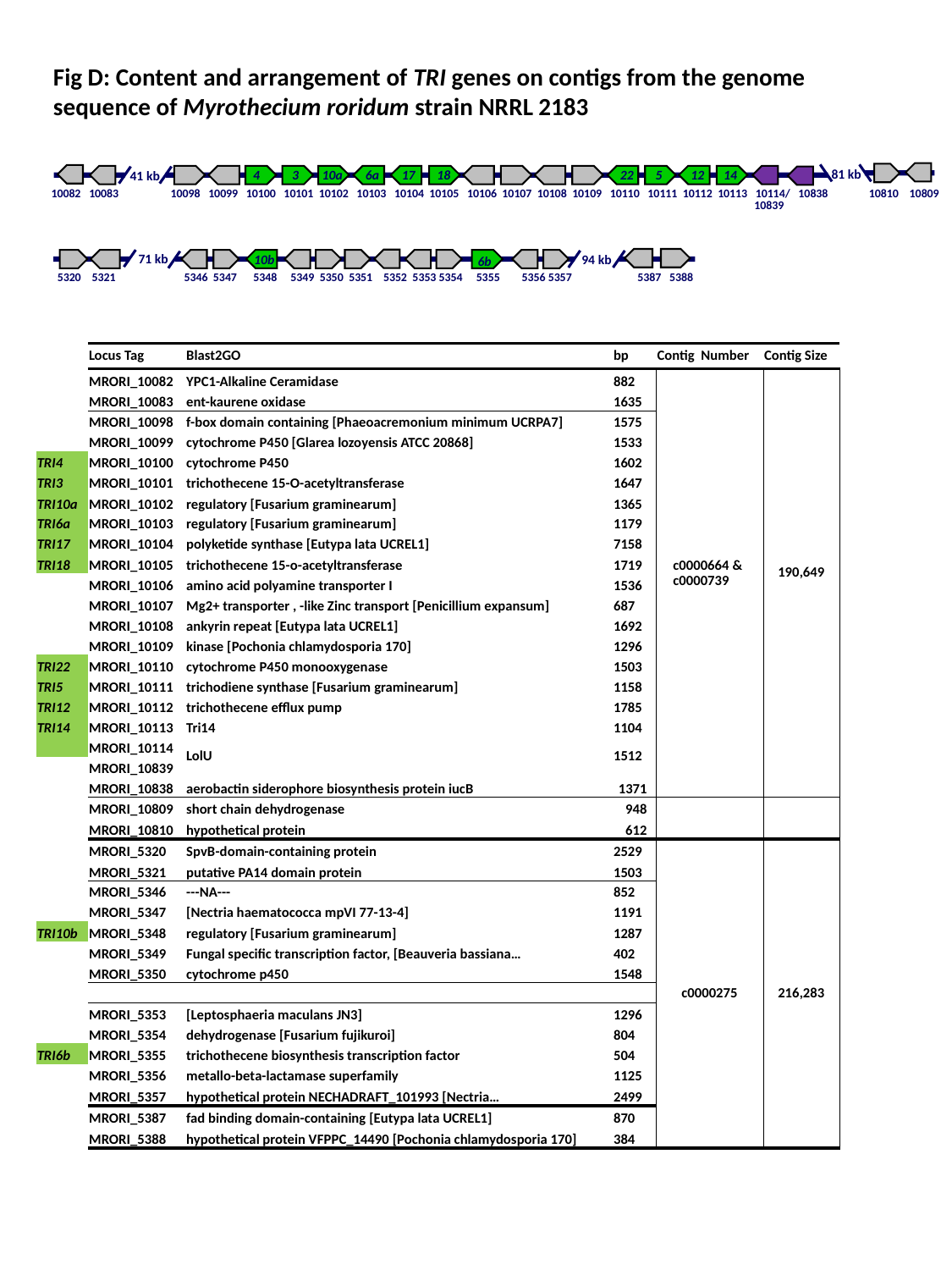

Fig D: Content and arrangement of TRI genes on contigs from the genome sequence of Myrothecium roridum strain NRRL 2183
81 kb
6a
18
5
4
3
10a
17
 22
12
14
41 kb
 10082 10083 10098 10099 10100 10101 10102 10103 10104 10105 10106 10107 10108 10109 10110 10111 10112 10113 10114/ 10838 10810 10809
 10839
71 kb
94 kb
10b
6b
5320 5321 5346 5347 5348 5349 5350 5351 5352 5353 5354 5355 5356 5357 5387 5388
| | Locus Tag | Blast2GO | bp | | Contig Number | Contig Size |
| --- | --- | --- | --- | --- | --- | --- |
| | MRORI\_10082 | YPC1-Alkaline Ceramidase | 882 | | c0000664 & | 190,649 |
| | MRORI\_10083 | ent-kaurene oxidase | 1635 | | | |
| | MRORI\_10098 | f-box domain containing [Phaeoacremonium minimum UCRPA7] | 1575 | | | |
| | MRORI\_10099 | cytochrome P450 [Glarea lozoyensis ATCC 20868] | 1533 | | | |
| TRI4 | MRORI\_10100 | cytochrome P450 | 1602 | | | |
| TRI3 | MRORI\_10101 | trichothecene 15-O-acetyltransferase | 1647 | | | |
| TRI10a | MRORI\_10102 | regulatory [Fusarium graminearum] | 1365 | | | |
| TRI6a | MRORI\_10103 | regulatory [Fusarium graminearum] | 1179 | | | |
| TRI17 | MRORI\_10104 | polyketide synthase [Eutypa lata UCREL1] | 7158 | | | |
| TRI18 | MRORI\_10105 | trichothecene 15-o-acetyltransferase | 1719 | | | |
| | MRORI\_10106 | amino acid polyamine transporter I | 1536 | | c0000739 | |
| | MRORI\_10107 | Mg2+ transporter , -like Zinc transport [Penicillium expansum] | 687 | | | |
| | MRORI\_10108 | ankyrin repeat [Eutypa lata UCREL1] | 1692 | | | |
| | MRORI\_10109 | kinase [Pochonia chlamydosporia 170] | 1296 | | | |
| TRI22 | MRORI\_10110 | cytochrome P450 monooxygenase | 1503 | | | |
| TRI5 | MRORI\_10111 | trichodiene synthase [Fusarium graminearum] | 1158 | | | |
| TRI12 | MRORI\_10112 | trichothecene efflux pump | 1785 | | | |
| TRI14 | MRORI\_10113 | Tri14 | 1104 | | | |
| | MRORI\_10114 | LolU | 1512 | | | |
| | MRORI\_10839 | | | | | |
| | MRORI\_10838 | aerobactin siderophore biosynthesis protein iucB | 1371 | | | |
| | MRORI\_10809 | short chain dehydrogenase | 948 | | | |
| | MRORI\_10810 | hypothetical protein | 612 | | | |
| | MRORI\_5320 | SpvB-domain-containing protein | 2529 | | c0000275 | 216,283 |
| | MRORI\_5321 | putative PA14 domain protein | 1503 | | | |
| | MRORI\_5346 | ---NA--- | 852 | | | |
| | MRORI\_5347 | [Nectria haematococca mpVI 77-13-4] | 1191 | | | |
| TRI10b | MRORI\_5348 | regulatory [Fusarium graminearum] | 1287 | | | |
| | MRORI\_5349 | Fungal specific transcription factor, [Beauveria bassiana… | 402 | | | |
| | MRORI\_5350 | cytochrome p450 | 1548 | | | |
| | | | | | | |
| | MRORI\_5353 | [Leptosphaeria maculans JN3] | 1296 | | | |
| | MRORI\_5354 | dehydrogenase [Fusarium fujikuroi] | 804 | | | |
| TRI6b | MRORI\_5355 | trichothecene biosynthesis transcription factor | 504 | | | |
| | MRORI\_5356 | metallo-beta-lactamase superfamily | 1125 | | | |
| | MRORI\_5357 | hypothetical protein NECHADRAFT\_101993 [Nectria… | 2499 | | | |
| | MRORI\_5387 | fad binding domain-containing [Eutypa lata UCREL1] | 870 | | | |
| | MRORI\_5388 | hypothetical protein VFPPC\_14490 [Pochonia chlamydosporia 170] | 384 | | | |

## Slide 6
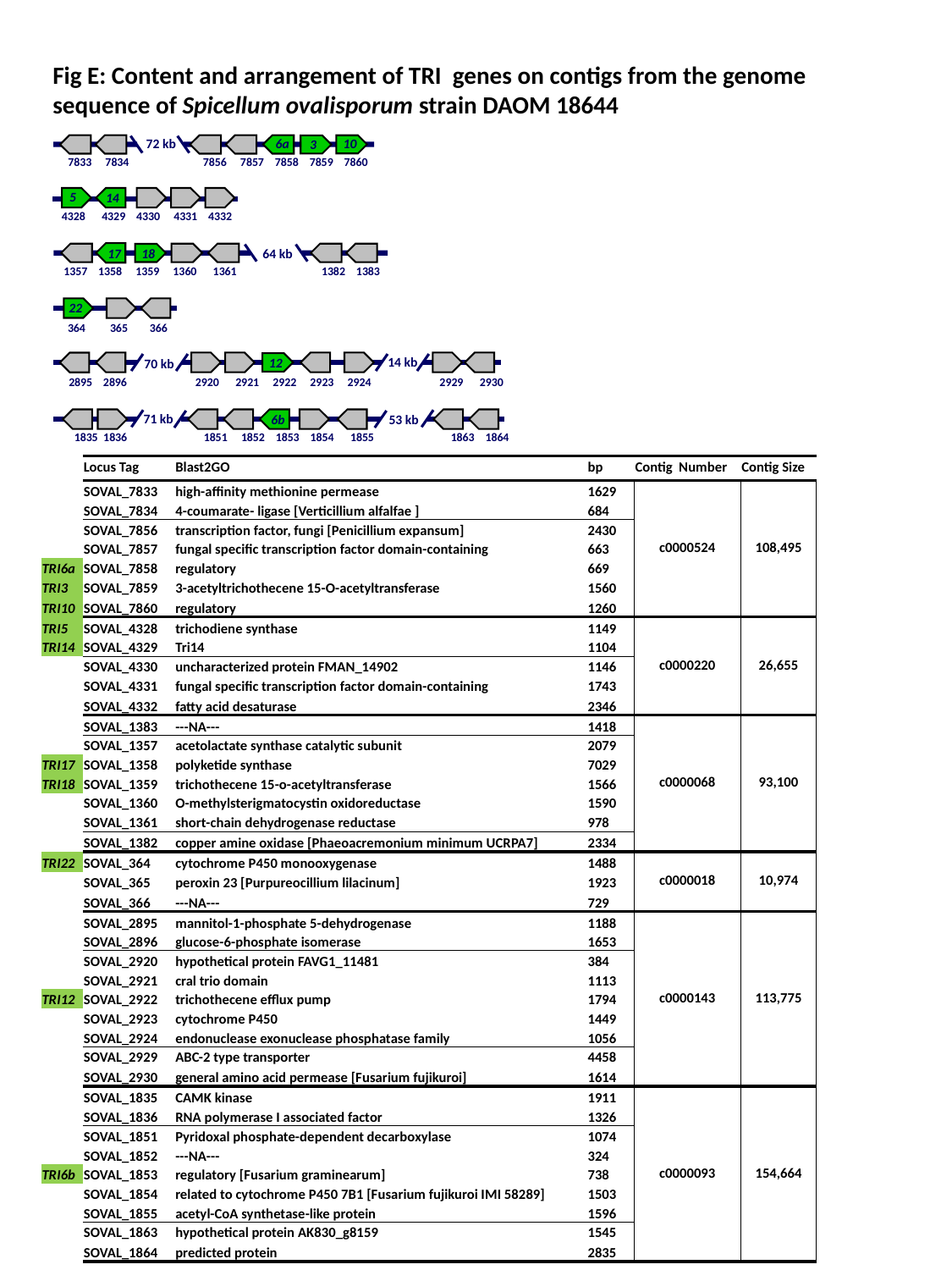

Fig E: Content and arrangement of TRI genes on contigs from the genome sequence of Spicellum ovalisporum strain DAOM 18644
6a
10
72 kb
3
7833 7834 7856 7857 7858 7859 7860
5
14
4328 4329 4330 4331 4332
17
18
64 kb
1357 1358 1359 1360 1361 1382 1383
22
364 365 366
 14 kb
12
 70 kb
2895 2896 2920 2921 2922 2923 2924 2929 2930
 71 kb
6b
 53 kb
1835 1836 1851 1852 1853 1854 1855 1863 1864
| | Locus Tag | Blast2GO | bp | | Contig Number | Contig Size |
| --- | --- | --- | --- | --- | --- | --- |
| | SOVAL\_7833 | high-affinity methionine permease | 1629 | | c0000524 | 108,495 |
| | SOVAL\_7834 | 4-coumarate- ligase [Verticillium alfalfae ] | 684 | | | |
| | SOVAL\_7856 | transcription factor, fungi [Penicillium expansum] | 2430 | | | |
| | SOVAL\_7857 | fungal specific transcription factor domain-containing | 663 | | | |
| TRI6a | SOVAL\_7858 | regulatory | 669 | | | |
| TRI3 | SOVAL\_7859 | 3-acetyltrichothecene 15-O-acetyltransferase | 1560 | | | |
| TRI10 | SOVAL\_7860 | regulatory | 1260 | | | |
| TRI5 | SOVAL\_4328 | trichodiene synthase | 1149 | | c0000220 | 26,655 |
| TRI14 | SOVAL\_4329 | Tri14 | 1104 | | | |
| | SOVAL\_4330 | uncharacterized protein FMAN\_14902 | 1146 | | | |
| | SOVAL\_4331 | fungal specific transcription factor domain-containing | 1743 | | | |
| | SOVAL\_4332 | fatty acid desaturase | 2346 | | | |
| | SOVAL\_1383 | ---NA--- | 1418 | | c0000068 | 93,100 |
| | SOVAL\_1357 | acetolactate synthase catalytic subunit | 2079 | | | |
| TRI17 | SOVAL\_1358 | polyketide synthase | 7029 | | | |
| TRI18 | SOVAL\_1359 | trichothecene 15-o-acetyltransferase | 1566 | | | |
| | SOVAL\_1360 | O-methylsterigmatocystin oxidoreductase | 1590 | | | |
| | SOVAL\_1361 | short-chain dehydrogenase reductase | 978 | | | |
| | SOVAL\_1382 | copper amine oxidase [Phaeoacremonium minimum UCRPA7] | 2334 | | | |
| TRI22 | SOVAL\_364 | cytochrome P450 monooxygenase | 1488 | | c0000018 | 10,974 |
| | SOVAL\_365 | peroxin 23 [Purpureocillium lilacinum] | 1923 | | | |
| | SOVAL\_366 | ---NA--- | 729 | | | |
| | SOVAL\_2895 | mannitol-1-phosphate 5-dehydrogenase | 1188 | | c0000143 | 113,775 |
| | SOVAL\_2896 | glucose-6-phosphate isomerase | 1653 | | | |
| | SOVAL\_2920 | hypothetical protein FAVG1\_11481 | 384 | | | |
| | SOVAL\_2921 | cral trio domain | 1113 | | | |
| TRI12 | SOVAL\_2922 | trichothecene efflux pump | 1794 | | | |
| | SOVAL\_2923 | cytochrome P450 | 1449 | | | |
| | SOVAL\_2924 | endonuclease exonuclease phosphatase family | 1056 | | | |
| | SOVAL\_2929 | ABC-2 type transporter | 4458 | | | |
| | SOVAL\_2930 | general amino acid permease [Fusarium fujikuroi] | 1614 | | | |
| | SOVAL\_1835 | CAMK kinase | 1911 | | c0000093 | 154,664 |
| | SOVAL\_1836 | RNA polymerase I associated factor | 1326 | | | |
| | SOVAL\_1851 | Pyridoxal phosphate-dependent decarboxylase | 1074 | | | |
| | SOVAL\_1852 | ---NA--- | 324 | | | |
| TRI6b | SOVAL\_1853 | regulatory [Fusarium graminearum] | 738 | | | |
| | SOVAL\_1854 | related to cytochrome P450 7B1 [Fusarium fujikuroi IMI 58289] | 1503 | | | |
| | SOVAL\_1855 | acetyl-CoA synthetase-like protein | 1596 | | | |
| | SOVAL\_1863 | hypothetical protein AK830\_g8159 | 1545 | | | |
| | SOVAL\_1864 | predicted protein | 2835 | | | |

## Slide 7
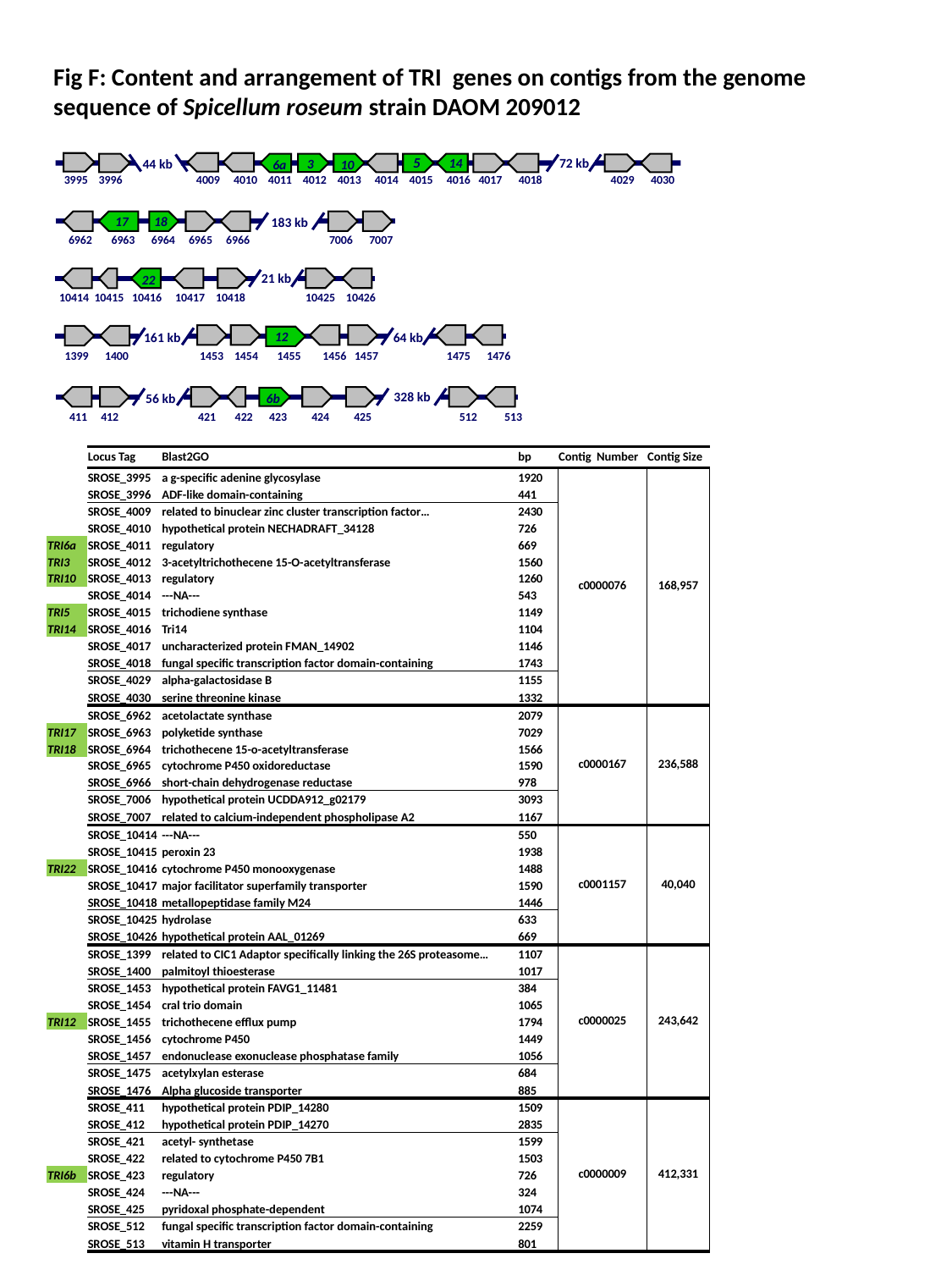

Fig F: Content and arrangement of TRI genes on contigs from the genome sequence of Spicellum roseum strain DAOM 209012
5
 72 kb
14
3
44 kb
6a
10
 3995 3996 4009 4010 4011 4012 4013 4014 4015 4016 4017 4018 4029 4030
17
18
 183 kb
 6962 6963 6964 6965 6966 7006 7007
21 kb
22
10414 10415 10416 10417 10418 10425 10426
12
161 kb
64 kb
1399 1400 1453 1454 1455 1456 1457 1475 1476
 328 kb
56 kb
6b
411 412 421 422 423 424 425 512 513
| | Locus Tag | Blast2GO | bp | | Contig Number | Contig Size |
| --- | --- | --- | --- | --- | --- | --- |
| | SROSE\_3995 | a g-specific adenine glycosylase | 1920 | | c0000076 | 168,957 |
| | SROSE\_3996 | ADF-like domain-containing | 441 | | | |
| | SROSE\_4009 | related to binuclear zinc cluster transcription factor… | 2430 | | | |
| | SROSE\_4010 | hypothetical protein NECHADRAFT\_34128 | 726 | | | |
| TRI6a | SROSE\_4011 | regulatory | 669 | | | |
| TRI3 | SROSE\_4012 | 3-acetyltrichothecene 15-O-acetyltransferase | 1560 | | | |
| TRI10 | SROSE\_4013 | regulatory | 1260 | | | |
| | SROSE\_4014 | ---NA--- | 543 | | | |
| TRI5 | SROSE\_4015 | trichodiene synthase | 1149 | | | |
| TRI14 | SROSE\_4016 | Tri14 | 1104 | | | |
| | SROSE\_4017 | uncharacterized protein FMAN\_14902 | 1146 | | | |
| | SROSE\_4018 | fungal specific transcription factor domain-containing | 1743 | | | |
| | SROSE\_4029 | alpha-galactosidase B | 1155 | | | |
| | SROSE\_4030 | serine threonine kinase | 1332 | | | |
| | SROSE\_6962 | acetolactate synthase | 2079 | | c0000167 | 236,588 |
| TRI17 | SROSE\_6963 | polyketide synthase | 7029 | | | |
| TRI18 | SROSE\_6964 | trichothecene 15-o-acetyltransferase | 1566 | | | |
| | SROSE\_6965 | cytochrome P450 oxidoreductase | 1590 | | | |
| | SROSE\_6966 | short-chain dehydrogenase reductase | 978 | | | |
| | SROSE\_7006 | hypothetical protein UCDDA912\_g02179 | 3093 | | | |
| | SROSE\_7007 | related to calcium-independent phospholipase A2 | 1167 | | | |
| | SROSE\_10414 | ---NA--- | 550 | | c0001157 | 40,040 |
| | SROSE\_10415 | peroxin 23 | 1938 | | | |
| TRI22 | SROSE\_10416 | cytochrome P450 monooxygenase | 1488 | | | |
| | SROSE\_10417 | major facilitator superfamily transporter | 1590 | | | |
| | SROSE\_10418 | metallopeptidase family M24 | 1446 | | | |
| | SROSE\_10425 | hydrolase | 633 | | | |
| | SROSE\_10426 | hypothetical protein AAL\_01269 | 669 | | | |
| | SROSE\_1399 | related to CIC1 Adaptor specifically linking the 26S proteasome… | 1107 | | c0000025 | 243,642 |
| | SROSE\_1400 | palmitoyl thioesterase | 1017 | | | |
| | SROSE\_1453 | hypothetical protein FAVG1\_11481 | 384 | | | |
| | SROSE\_1454 | cral trio domain | 1065 | | | |
| TRI12 | SROSE\_1455 | trichothecene efflux pump | 1794 | | | |
| | SROSE\_1456 | cytochrome P450 | 1449 | | | |
| | SROSE\_1457 | endonuclease exonuclease phosphatase family | 1056 | | | |
| | SROSE\_1475 | acetylxylan esterase | 684 | | | |
| | SROSE\_1476 | Alpha glucoside transporter | 885 | | | |
| | SROSE\_411 | hypothetical protein PDIP\_14280 | 1509 | | c0000009 | 412,331 |
| | SROSE\_412 | hypothetical protein PDIP\_14270 | 2835 | | | |
| | SROSE\_421 | acetyl- synthetase | 1599 | | | |
| | SROSE\_422 | related to cytochrome P450 7B1 | 1503 | | | |
| TRI6b | SROSE\_423 | regulatory | 726 | | | |
| | SROSE\_424 | ---NA--- | 324 | | | |
| | SROSE\_425 | pyridoxal phosphate-dependent | 1074 | | | |
| | SROSE\_512 | fungal specific transcription factor domain-containing | 2259 | | | |
| | SROSE\_513 | vitamin H transporter | 801 | | | |

## Slide 8
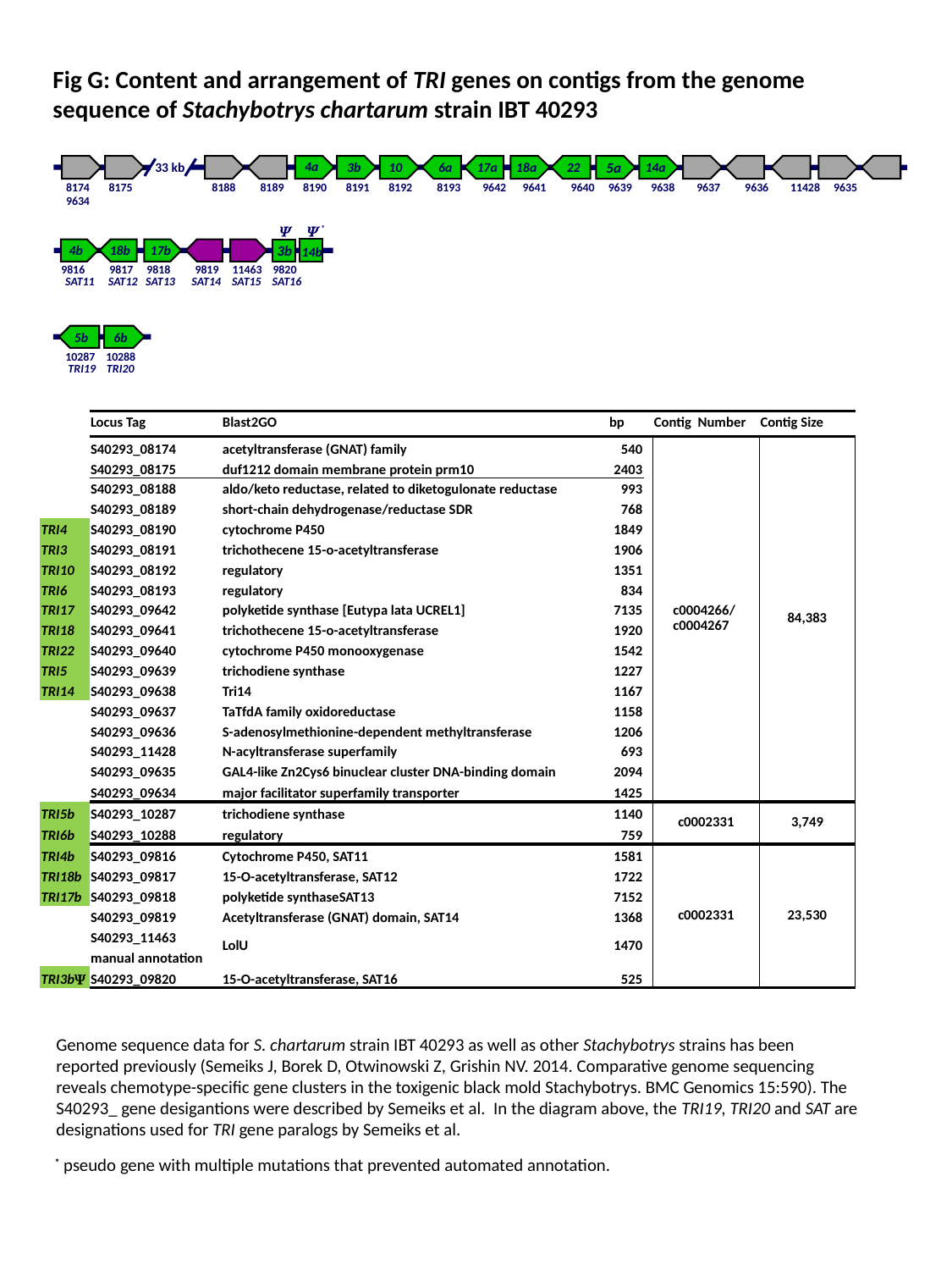

Fig G: Content and arrangement of TRI genes on contigs from the genome sequence of Stachybotrys chartarum strain IBT 40293
5a
4a
33 kb
3b
10
 6a
17a
18a
 22
14a
8174 8175 8188 8189 8190 8191 8192 8193 9642 9641 9640 9639 9638 9637 9636 11428 9635 9634

 *
3b
4b
18b
17b
14b
9816 9817 9818 9819 11463 9820
SAT11 SAT12 SAT13 SAT14 SAT15 SAT16
5b
6b
TRI19 TRI20
10287 10288
| | Locus Tag | Blast2GO | bp | | Contig Number | Contig Size |
| --- | --- | --- | --- | --- | --- | --- |
| | S40293\_08174 | acetyltransferase (GNAT) family | 540 | | | 84,383 |
| | S40293\_08175 | duf1212 domain membrane protein prm10 | 2403 | | | |
| | S40293\_08188 | aldo/keto reductase, related to diketogulonate reductase | 993 | | | |
| | S40293\_08189 | short-chain dehydrogenase/reductase SDR | 768 | | | |
| TRI4 | S40293\_08190 | cytochrome P450 | 1849 | | | |
| TRI3 | S40293\_08191 | trichothecene 15-o-acetyltransferase | 1906 | | | |
| TRI10 | S40293\_08192 | regulatory | 1351 | | | |
| TRI6 | S40293\_08193 | regulatory | 834 | | | |
| TRI17 | S40293\_09642 | polyketide synthase [Eutypa lata UCREL1] | 7135 | | c0004266/ | |
| TRI18 | S40293\_09641 | trichothecene 15-o-acetyltransferase | 1920 | | c0004267 | |
| TRI22 | S40293\_09640 | cytochrome P450 monooxygenase | 1542 | | | |
| TRI5 | S40293\_09639 | trichodiene synthase | 1227 | | | |
| TRI14 | S40293\_09638 | Tri14 | 1167 | | | |
| | S40293\_09637 | TaTfdA family oxidoreductase | 1158 | | | |
| | S40293\_09636 | S-adenosylmethionine-dependent methyltransferase | 1206 | | | |
| | S40293\_11428 | N-acyltransferase superfamily | 693 | | | |
| | S40293\_09635 | GAL4-like Zn2Cys6 binuclear cluster DNA-binding domain | 2094 | | | |
| | S40293\_09634 | major facilitator superfamily transporter | 1425 | | | |
| TRI5b | S40293\_10287 | trichodiene synthase | 1140 | | c0002331 | 3,749 |
| TRI6b | S40293\_10288 | regulatory | 759 | | | |
| TRI4b | S40293\_09816 | Cytochrome P450, SAT11 | 1581 | | c0002331 | 23,530 |
| TRI18b | S40293\_09817 | 15-O-acetyltransferase, SAT12 | 1722 | | | |
| TRI17b | S40293\_09818 | polyketide synthaseSAT13 | 7152 | | | |
| | S40293\_09819 | Acetyltransferase (GNAT) domain, SAT14 | 1368 | | | |
| | S40293\_11463 | LolU | 1470 | | | |
| | manual annotation | | | | | |
| TRI3bY | S40293\_09820 | 15-O-acetyltransferase, SAT16 | 525 | | | |
Genome sequence data for S. chartarum strain IBT 40293 as well as other Stachybotrys strains has been reported previously (Semeiks J, Borek D, Otwinowski Z, Grishin NV. 2014. Comparative genome sequencing reveals chemotype-specific gene clusters in the toxigenic black mold Stachybotrys. BMC Genomics 15:590). The S40293_ gene desigantions were described by Semeiks et al. In the diagram above, the TRI19, TRI20 and SAT are designations used for TRI gene paralogs by Semeiks et al.
* pseudo gene with multiple mutations that prevented automated annotation.

## Slide 9
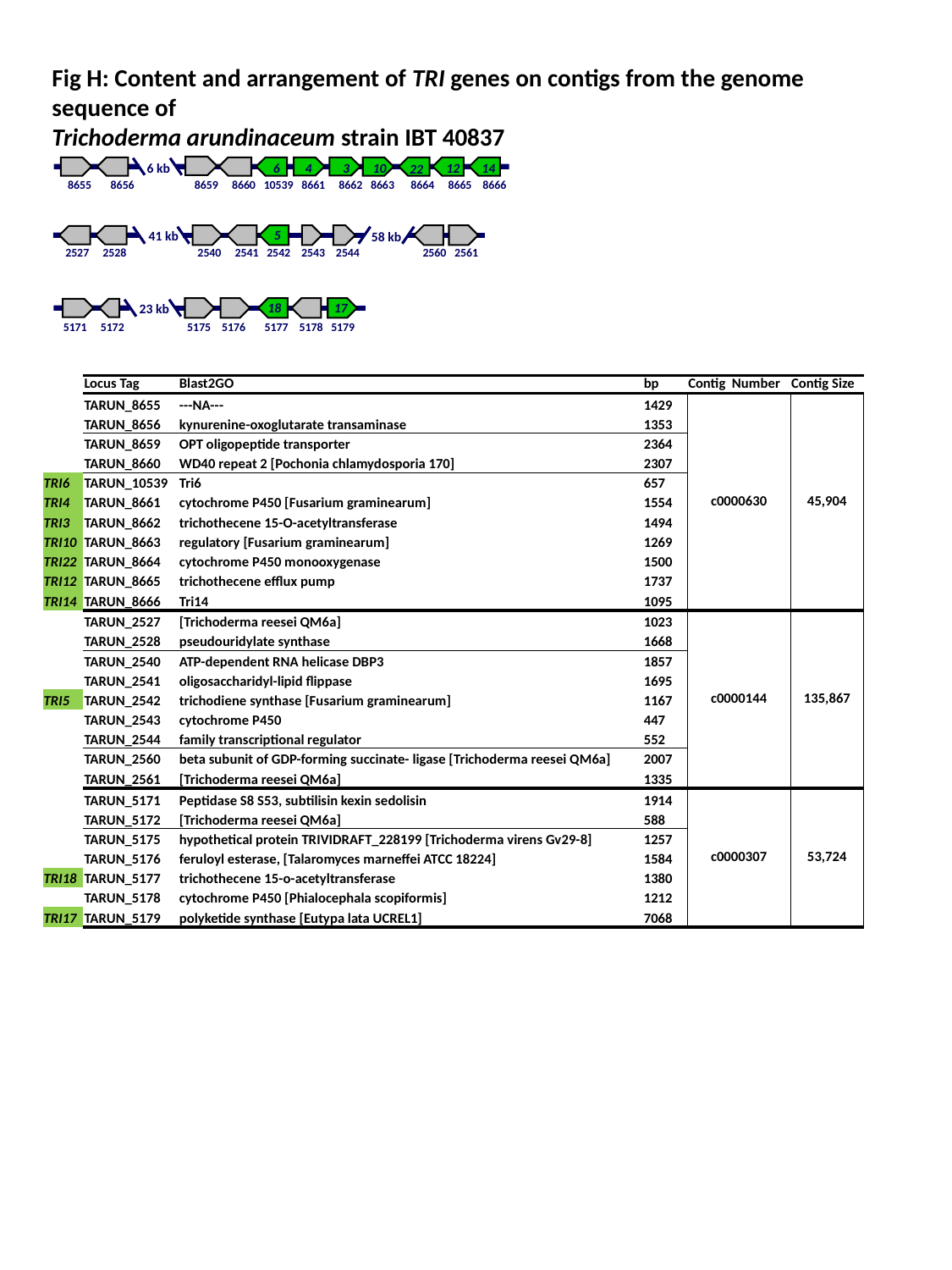

Fig H: Content and arrangement of TRI genes on contigs from the genome sequence of
Trichoderma arundinaceum strain IBT 40837
6
4
3
10
12
14
6 kb
22
8655 8656 8659 8660 10539 8661 8662 8663 8664 8665 8666
5
41 kb
58 kb
2527 2528 2540 2541 2542 2543 2544 2560 2561
18
17
23 kb
5171 5172 5175 5176 5177 5178 5179
| | Locus Tag | Blast2GO | bp | | Contig Number | Contig Size |
| --- | --- | --- | --- | --- | --- | --- |
| | TARUN\_8655 | ---NA--- | 1429 | | c0000630 | 45,904 |
| | TARUN\_8656 | kynurenine-oxoglutarate transaminase | 1353 | | | |
| | TARUN\_8659 | OPT oligopeptide transporter | 2364 | | | |
| | TARUN\_8660 | WD40 repeat 2 [Pochonia chlamydosporia 170] | 2307 | | | |
| TRI6 | TARUN\_10539 | Tri6 | 657 | | | |
| TRI4 | TARUN\_8661 | cytochrome P450 [Fusarium graminearum] | 1554 | | | |
| TRI3 | TARUN\_8662 | trichothecene 15-O-acetyltransferase | 1494 | | | |
| TRI10 | TARUN\_8663 | regulatory [Fusarium graminearum] | 1269 | | | |
| TRI22 | TARUN\_8664 | cytochrome P450 monooxygenase | 1500 | | | |
| TRI12 | TARUN\_8665 | trichothecene efflux pump | 1737 | | | |
| TRI14 | TARUN\_8666 | Tri14 | 1095 | | | |
| | TARUN\_2527 | [Trichoderma reesei QM6a] | 1023 | | c0000144 | 135,867 |
| | TARUN\_2528 | pseudouridylate synthase | 1668 | | | |
| | TARUN\_2540 | ATP-dependent RNA helicase DBP3 | 1857 | | | |
| | TARUN\_2541 | oligosaccharidyl-lipid flippase | 1695 | | | |
| TRI5 | TARUN\_2542 | trichodiene synthase [Fusarium graminearum] | 1167 | | | |
| | TARUN\_2543 | cytochrome P450 | 447 | | | |
| | TARUN\_2544 | family transcriptional regulator | 552 | | | |
| | TARUN\_2560 | beta subunit of GDP-forming succinate- ligase [Trichoderma reesei QM6a] | 2007 | | | |
| | TARUN\_2561 | [Trichoderma reesei QM6a] | 1335 | | | |
| | TARUN\_5171 | Peptidase S8 S53, subtilisin kexin sedolisin | 1914 | | c0000307 | 53,724 |
| | TARUN\_5172 | [Trichoderma reesei QM6a] | 588 | | | |
| | TARUN\_5175 | hypothetical protein TRIVIDRAFT\_228199 [Trichoderma virens Gv29-8] | 1257 | | | |
| | TARUN\_5176 | feruloyl esterase, [Talaromyces marneffei ATCC 18224] | 1584 | | | |
| TRI18 | TARUN\_5177 | trichothecene 15-o-acetyltransferase | 1380 | | | |
| | TARUN\_5178 | cytochrome P450 [Phialocephala scopiformis] | 1212 | | | |
| TRI17 | TARUN\_5179 | polyketide synthase [Eutypa lata UCREL1] | 7068 | | | |

## Slide 10
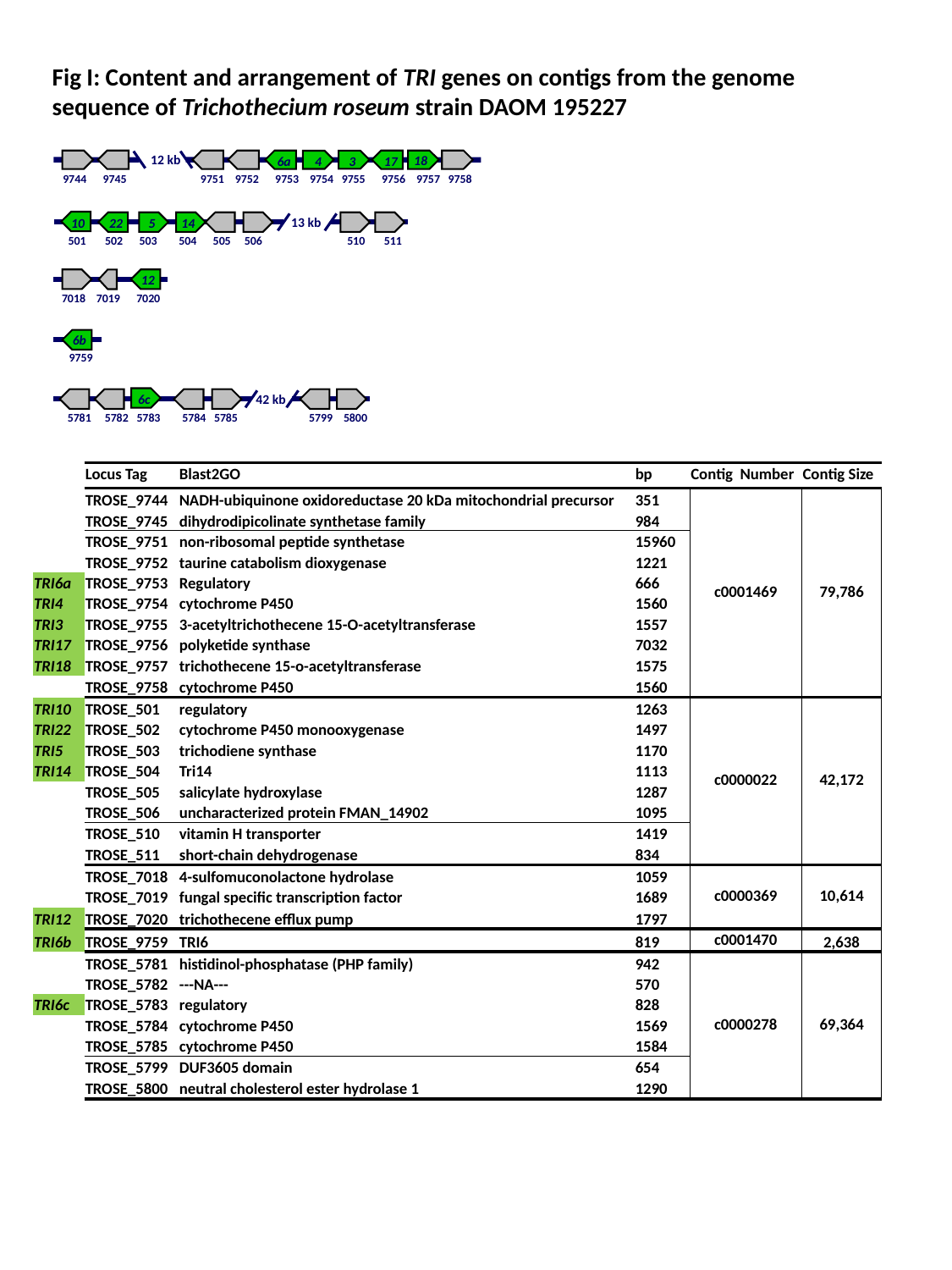

Fig I: Content and arrangement of TRI genes on contigs from the genome sequence of Trichothecium roseum strain DAOM 195227
12 kb
18
6a
4
3
17
9744 9745 9751 9752 9753 9754 9755 9756 9757 9758
 13 kb
10
22
5
14
 501 502 503 504 505 506 510 511
12
7018 7019 7020
6b
9759
42 kb
6c
5781 5782 5783 5784 5785 5799 5800
| | Locus Tag | Blast2GO | bp | | Contig Number | Contig Size |
| --- | --- | --- | --- | --- | --- | --- |
| | TROSE\_9744 | NADH-ubiquinone oxidoreductase 20 kDa mitochondrial precursor | 351 | | c0001469 | 79,786 |
| | TROSE\_9745 | dihydrodipicolinate synthetase family | 984 | | | |
| | TROSE\_9751 | non-ribosomal peptide synthetase | 15960 | | | |
| | TROSE\_9752 | taurine catabolism dioxygenase | 1221 | | | |
| TRI6a | TROSE\_9753 | Regulatory | 666 | | | |
| TRI4 | TROSE\_9754 | cytochrome P450 | 1560 | | | |
| TRI3 | TROSE\_9755 | 3-acetyltrichothecene 15-O-acetyltransferase | 1557 | | | |
| TRI17 | TROSE\_9756 | polyketide synthase | 7032 | | | |
| TRI18 | TROSE\_9757 | trichothecene 15-o-acetyltransferase | 1575 | | | |
| | TROSE\_9758 | cytochrome P450 | 1560 | | | |
| TRI10 | TROSE\_501 | regulatory | 1263 | | c0000022 | 42,172 |
| TRI22 | TROSE\_502 | cytochrome P450 monooxygenase | 1497 | | | |
| TRI5 | TROSE\_503 | trichodiene synthase | 1170 | | | |
| TRI14 | TROSE\_504 | Tri14 | 1113 | | | |
| | TROSE\_505 | salicylate hydroxylase | 1287 | | | |
| | TROSE\_506 | uncharacterized protein FMAN\_14902 | 1095 | | | |
| | TROSE\_510 | vitamin H transporter | 1419 | | | |
| | TROSE\_511 | short-chain dehydrogenase | 834 | | | |
| | TROSE\_7018 | 4-sulfomuconolactone hydrolase | 1059 | | c0000369 | 10,614 |
| | TROSE\_7019 | fungal specific transcription factor | 1689 | | | |
| TRI12 | TROSE\_7020 | trichothecene efflux pump | 1797 | | | |
| TRI6b | TROSE\_9759 | TRI6 | 819 | | c0001470 | 2,638 |
| | TROSE\_5781 | histidinol-phosphatase (PHP family) | 942 | | c0000278 | 69,364 |
| | TROSE\_5782 | ---NA--- | 570 | | | |
| TRI6c | TROSE\_5783 | regulatory | 828 | | | |
| | TROSE\_5784 | cytochrome P450 | 1569 | | | |
| | TROSE\_5785 | cytochrome P450 | 1584 | | | |
| | TROSE\_5799 | DUF3605 domain | 654 | | | |
| | TROSE\_5800 | neutral cholesterol ester hydrolase 1 | 1290 | | | |
