# Supplementary material for: Evolution of structural diversity of trichothecenes, a family of toxins produced by plant pathogenic and entomopathogenic fungi
Source: PLoS Pathog. 2018 Apr 12;14(4):e1006946. doi: 10.1371/journal.ppat.1006946 (PMC5897003; doi:10.1371/journal.ppat.1006946)
Supplement: S3 Fig — The nucleotide sequence of the query is shown below the BLASTx results. (PPTX) [file ppat.1006946.s006.pptx]

## Slide 1
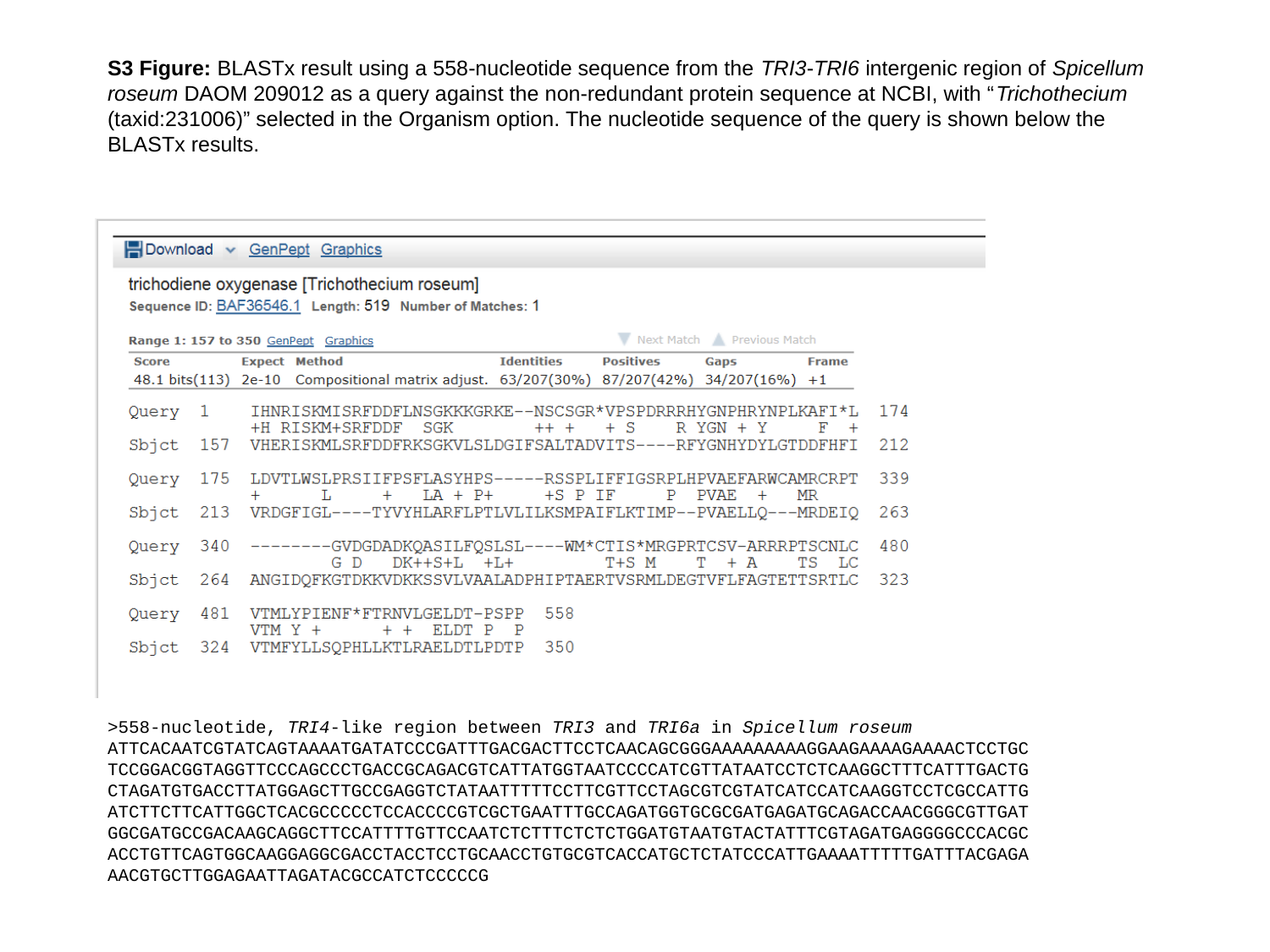

S3 Figure: BLASTx result using a 558-nucleotide sequence from the TRI3-TRI6 intergenic region of Spicellum roseum DAOM 209012 as a query against the non-redundant protein sequence at NCBI, with “Trichothecium (taxid:231006)” selected in the Organism option. The nucleotide sequence of the query is shown below the BLASTx results.
>558-nucleotide, TRI4-like region between TRI3 and TRI6a in Spicellum roseum ATTCACAATCGTATCAGTAAAATGATATCCCGATTTGACGACTTCCTCAACAGCGGGAAAAAAAAAGGAAGAAAAGAAAACTCCTGCTCCGGACGGTAGGTTCCCAGCCCTGACCGCAGACGTCATTATGGTAATCCCCATCGTTATAATCCTCTCAAGGCTTTCATTTGACTGCTAGATGTGACCTTATGGAGCTTGCCGAGGTCTATAATTTTTCCTTCGTTCCTAGCGTCGTATCATCCATCAAGGTCCTCGCCATTGATCTTCTTCATTGGCTCACGCCCCCTCCACCCCGTCGCTGAATTTGCCAGATGGTGCGCGATGAGATGCAGACCAACGGGCGTTGATGGCGATGCCGACAAGCAGGCTTCCATTTTGTTCCAATCTCTTTCTCTCTGGATGTAATGTACTATTTCGTAGATGAGGGGCCCACGCACCTGTTCAGTGGCAAGGAGGCGACCTACCTCCTGCAACCTGTGCGTCACCATGCTCTATCCCATTGAAAATTTTTGATTTACGAGAAACGTGCTTGGAGAATTAGATACGCCATCTCCCCCG
