# Supplementary material for: Evolution of structural diversity of trichothecenes, a family of toxins produced by plant pathogenic and entomopathogenic fungi
Source: PLoS Pathog. 2018 Apr 12;14(4):e1006946. doi: 10.1371/journal.ppat.1006946 (PMC5897003; doi:10.1371/journal.ppat.1006946)
Supplement: S4 Fig — Peaks corresponding to trichodermol (4-hydroxy EPT), trichodermin (4-O-acetyl EPT), and 8-deoxy trichothecin (4-O-butenoyl EPT) are shown. Based on mass spectral fragmentation patterns, the unlabeled peaks at 5 min and 6.1 min do not correspond to trichothecenes. (PPTX) [file ppat.1006946.s007.pptx]

## Slide 1
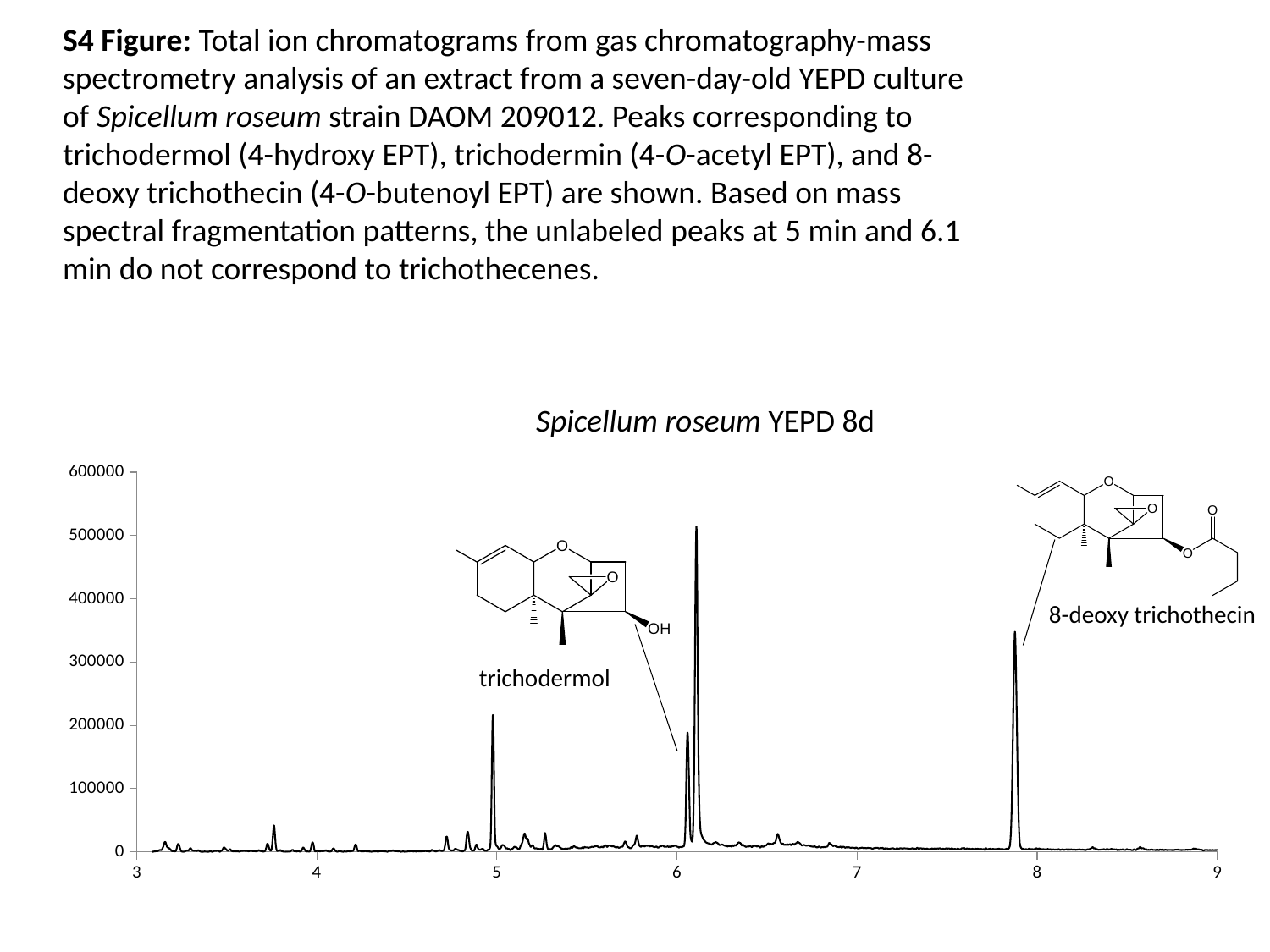

S4 Figure: Total ion chromatograms from gas chromatography-mass spectrometry analysis of an extract from a seven-day-old YEPD culture of Spicellum roseum strain DAOM 209012. Peaks corresponding to trichodermol (4-hydroxy EPT), trichodermin (4-O-acetyl EPT), and 8-deoxy trichothecin (4-O-butenoyl EPT) are shown. Based on mass spectral fragmentation patterns, the unlabeled peaks at 5 min and 6.1 min do not correspond to trichothecenes.
Spicellum roseum YEPD 8d
### Chart
| Category | |
|---|---|8-deoxy trichothecin
trichodermol
