# Supplementary material for: Evolution of structural diversity of trichothecenes, a family of toxins produced by plant pathogenic and entomopathogenic fungi
Source: PLoS Pathog. 2018 Apr 12;14(4):e1006946. doi: 10.1371/journal.ppat.1006946 (PMC5897003; doi:10.1371/journal.ppat.1006946)
Supplement: S5 Fig — Results are presented for the wild-type progenitor strain (blue trace) and complementation strains tri17.MrT17.C3 (black trace) and tri17.MrT17.C4 (red trace). The trace for tri17 mutant strain tri17.139 (purple trace) does not rise above the base line. Identity of harzianum A in samples was confirmed by comparison of mass spectra to the spectrum of a purified harzianum A standard, the identity of which was confirmed by 1H and 13C NMR. Strains were grown in YEPD medium for one week. (PPTX) [file ppat.1006946.s008.pptx]

## Slide 1
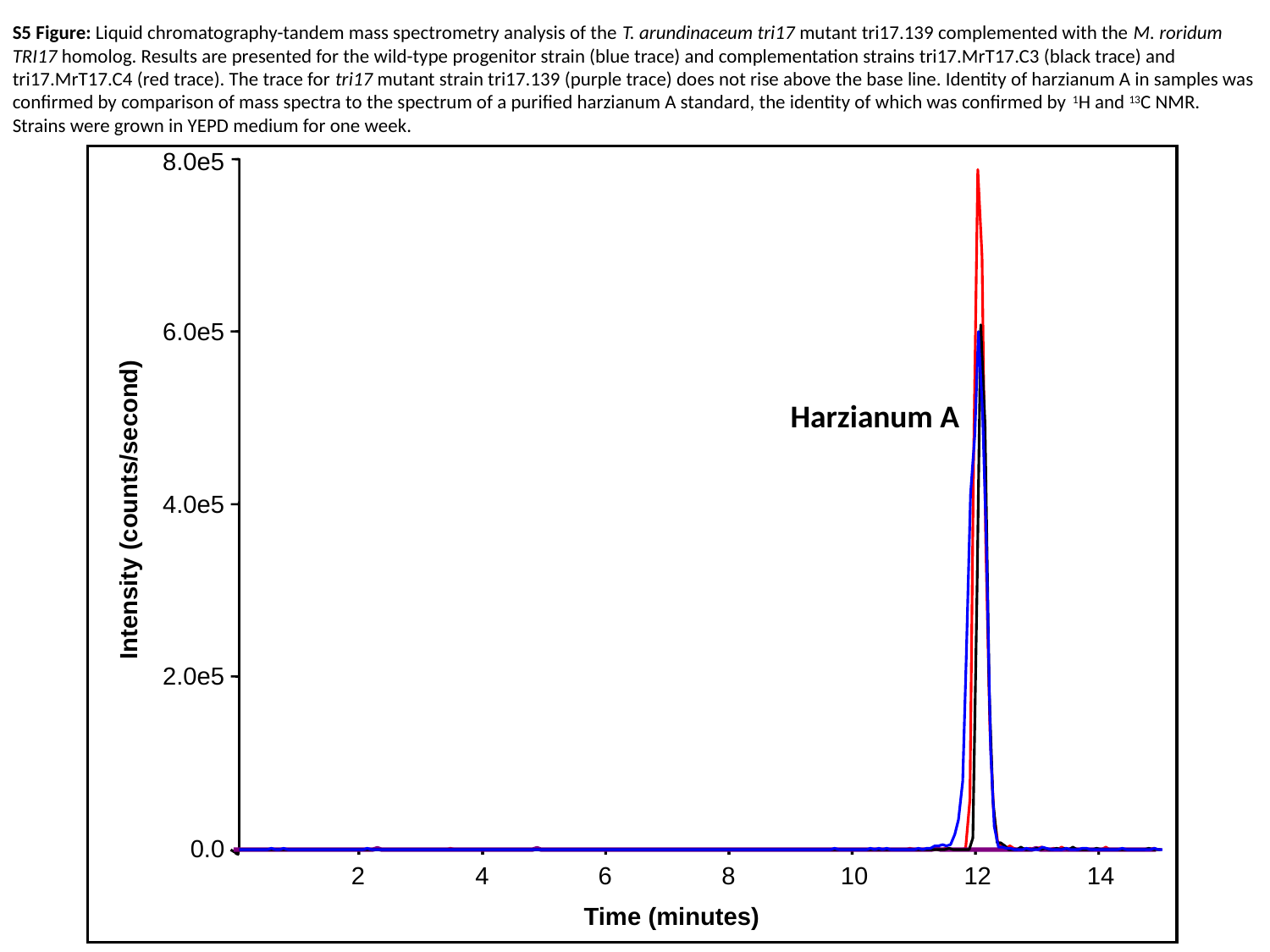

S5 Figure: Liquid chromatography-tandem mass spectrometry analysis of the T. arundinaceum tri17 mutant tri17.139 complemented with the M. roridum TRI17 homolog. Results are presented for the wild-type progenitor strain (blue trace) and complementation strains tri17.MrT17.C3 (black trace) and tri17.MrT17.C4 (red trace). The trace for tri17 mutant strain tri17.139 (purple trace) does not rise above the base line. Identity of harzianum A in samples was confirmed by comparison of mass spectra to the spectrum of a purified harzianum A standard, the identity of which was confirmed by 1H and 13C NMR. Strains were grown in YEPD medium for one week.
8.0e5
6.0e5
4.0e5
Intensity (counts/second)
2.0e5
0.0
2
4
6
8
10
12
14
Time (minutes)
Harzianum A
