# Supplementary material for: Evolution of structural diversity of trichothecenes, a family of toxins produced by plant pathogenic and entomopathogenic fungi
Source: PLoS Pathog. 2018 Apr 12;14(4):e1006946. doi: 10.1371/journal.ppat.1006946 (PMC5897003; doi:10.1371/journal.ppat.1006946)
Supplement: S6 Fig — Fig A: Total ion chromatogram of an ethyl acetate extract of the culture (above), and mass spectrum of 4-hydroxy isotrichodermin (below). Fig B: 13C (left) and 1H (right) NMR spectra of 4-hydroxy isotrichodermin. (PPTX) [file ppat.1006946.s009.pptx]

## Slide 1
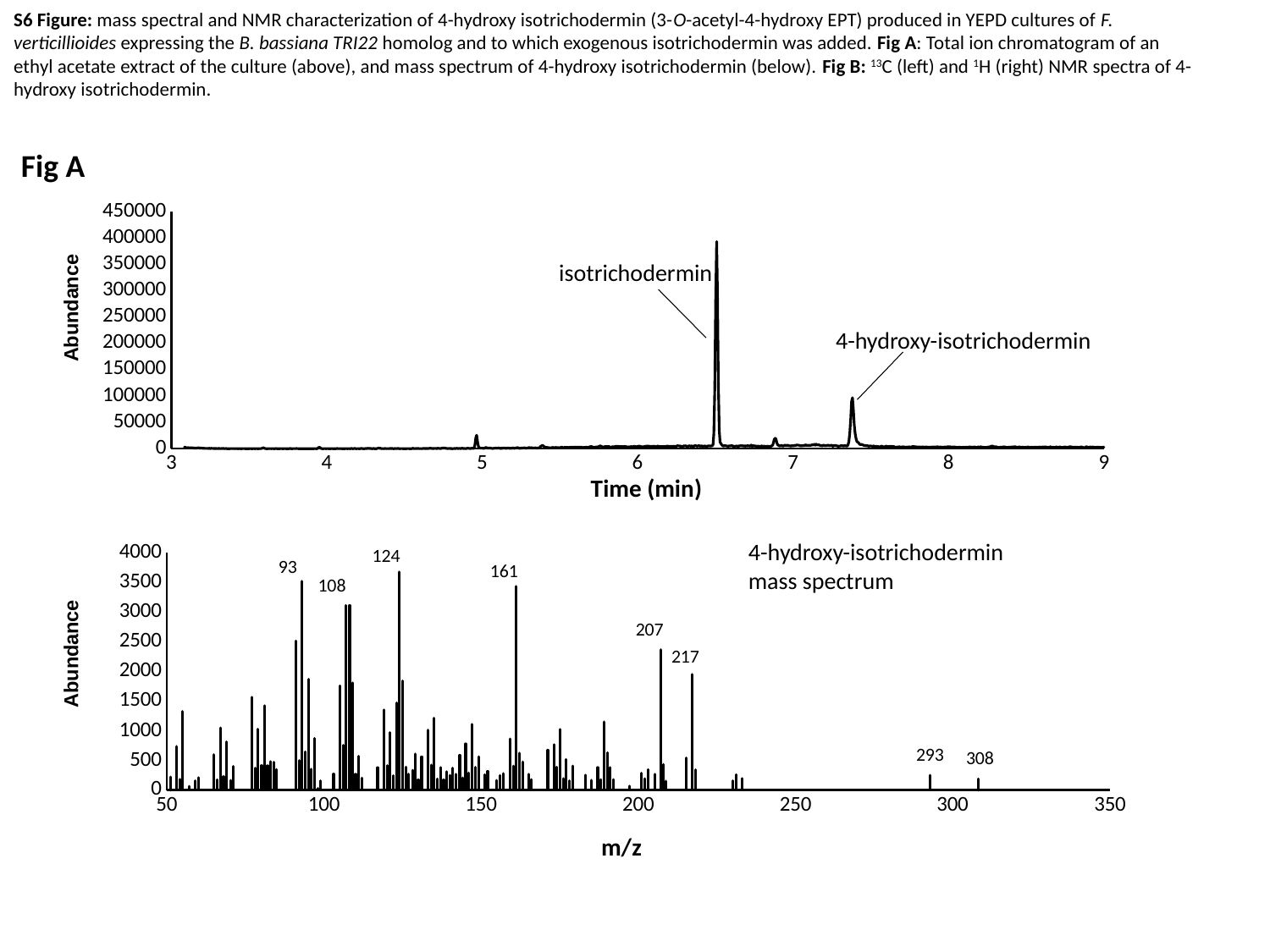

S6 Figure: mass spectral and NMR characterization of 4-hydroxy isotrichodermin (3-O-acetyl-4-hydroxy EPT) produced in YEPD cultures of F. verticillioides expressing the B. bassiana TRI22 homolog and to which exogenous isotrichodermin was added. Fig A: Total ion chromatogram of an ethyl acetate extract of the culture (above), and mass spectrum of 4-hydroxy isotrichodermin (below). Fig B: 13C (left) and 1H (right) NMR spectra of 4-hydroxy isotrichodermin.
Fig A
### Chart
| Category | |
|---|---|isotrichodermin
Abundance
4-hydroxy-isotrichodermin
Time (min)
4-hydroxy-isotrichodermin
mass spectrum
### Chart
| Category | |
|---|---|124
93
161
108
207
Abundance
217
293
308
m/z

## Slide 2
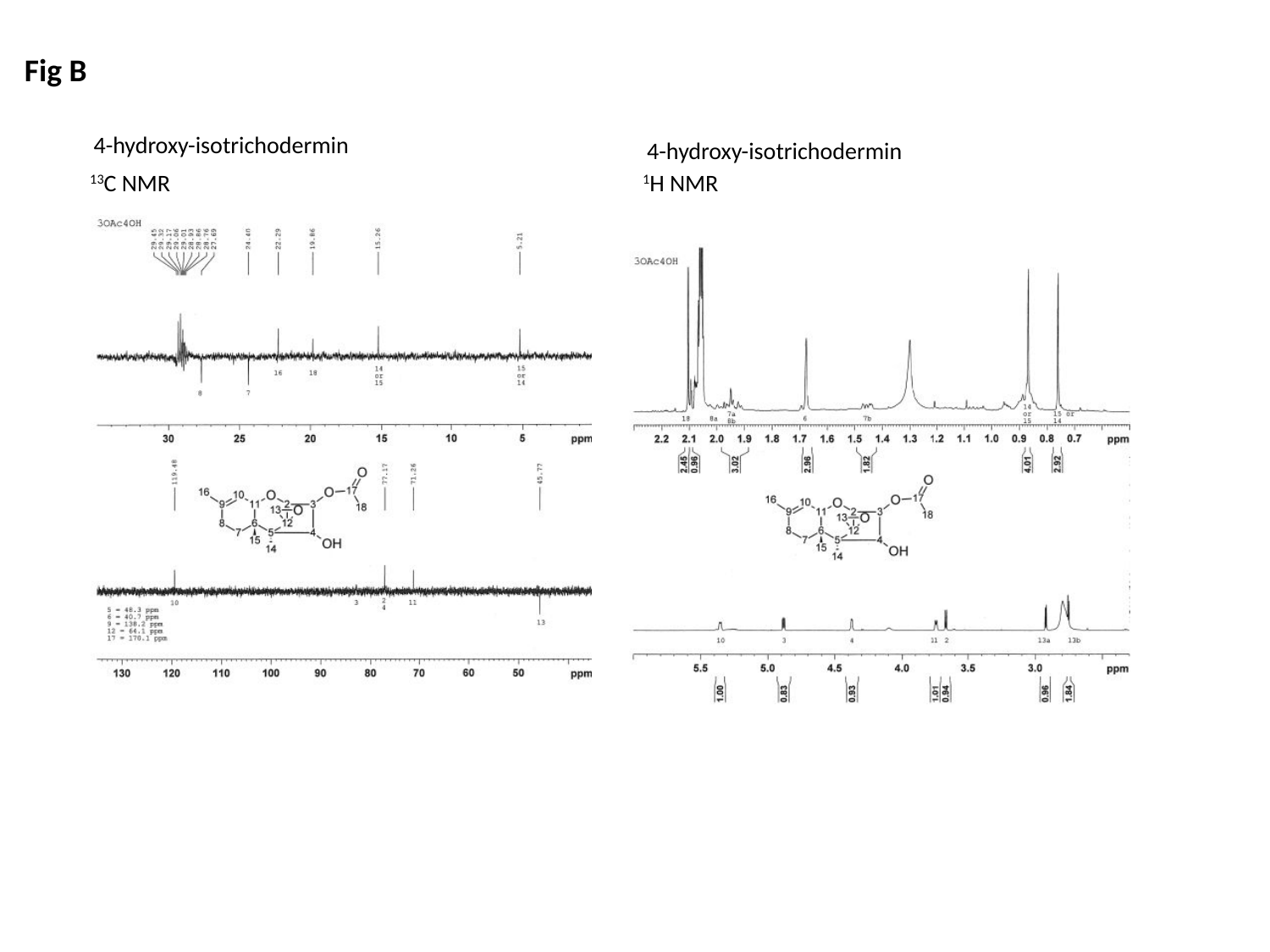

Fig B
4-hydroxy-isotrichodermin
4-hydroxy-isotrichodermin
13C NMR
1H NMR
