# Supplementary material for: Evolution of structural diversity of trichothecenes, a family of toxins produced by plant pathogenic and entomopathogenic fungi
Source: PLoS Pathog. 2018 Apr 12;14(4):e1006946. doi: 10.1371/journal.ppat.1006946 (PMC5897003; doi:10.1371/journal.ppat.1006946)
Supplement: S7 Fig — In the tree shown here, a distantly related homolog was selected for each of the three TRI genes. For TRI3, we selected a homolog of TRI18 as the outgroup. We searched GenBank by BLASTx [75] to identify non-TRI gene homologs to use at outgroups in the TRI5 and TRI14 trees; the outgroups are represented by their GenBank accession numbers. To generate trees, deduced amino acid sequences of each gene were aligned with Muscle as implemented in MEGA7 [79], and trees were inferred using the maximum likelihood method with ultrafast bootstrapping [83] as implemented in IQ-Tree [73]. Numbers near branches are bootstrap values based on 1000 pseudoreplicates. (PPTX) [file ppat.1006946.s010.pptx]

## Slide 1
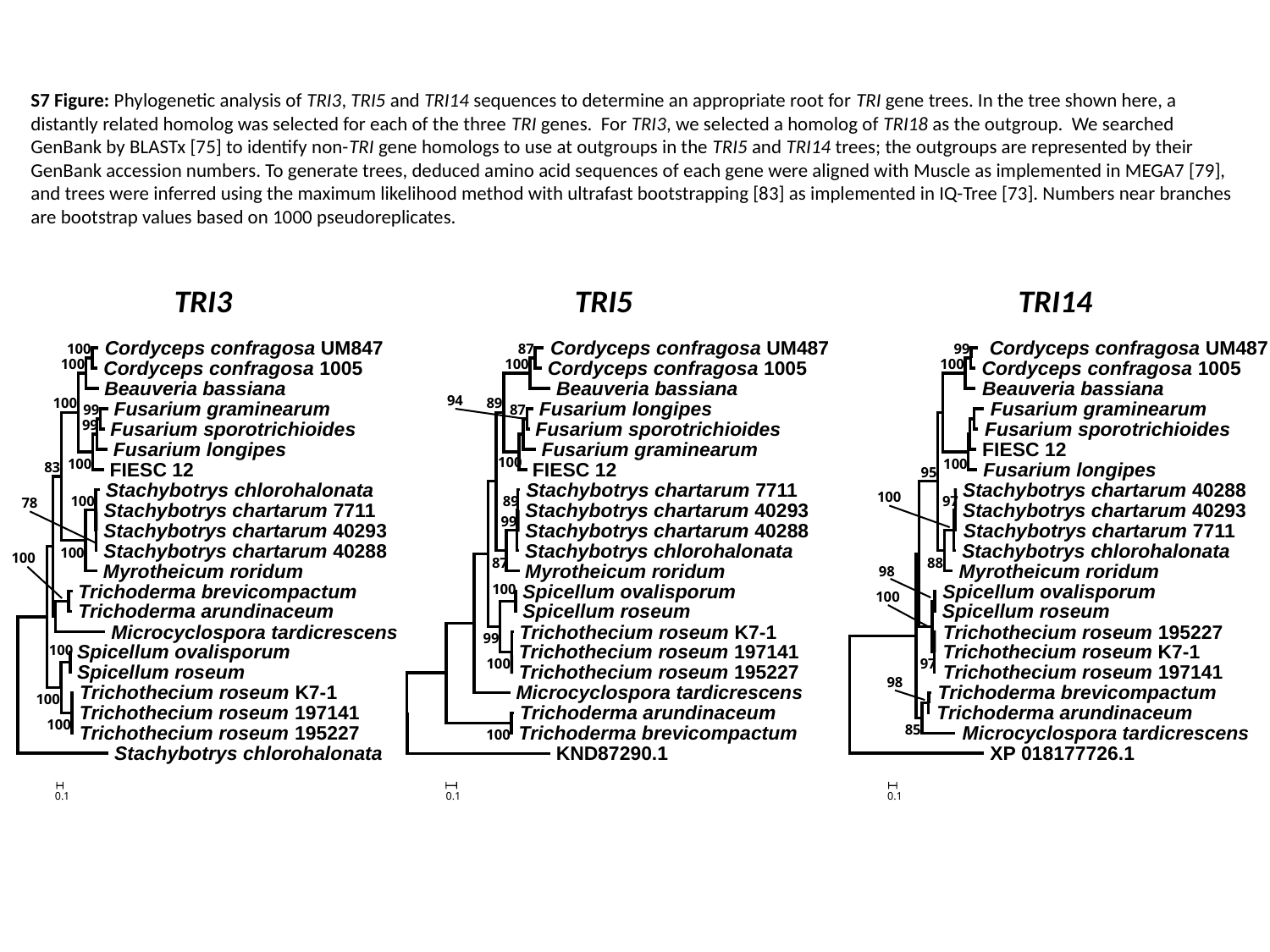

S7 Figure: Phylogenetic analysis of TRI3, TRI5 and TRI14 sequences to determine an appropriate root for TRI gene trees. In the tree shown here, a distantly related homolog was selected for each of the three TRI genes. For TRI3, we selected a homolog of TRI18 as the outgroup. We searched GenBank by BLASTx [75] to identify non-TRI gene homologs to use at outgroups in the TRI5 and TRI14 trees; the outgroups are represented by their GenBank accession numbers. To generate trees, deduced amino acid sequences of each gene were aligned with Muscle as implemented in MEGA7 [79], and trees were inferred using the maximum likelihood method with ultrafast bootstrapping [83] as implemented in IQ-Tree [73]. Numbers near branches are bootstrap values based on 1000 pseudoreplicates.
TRI3
TRI5
TRI14
 Cordyceps confragosa UM847
100
100
 Cordyceps confragosa 1005
 Beauveria bassiana
100
 Fusarium graminearum
99
99
 Fusarium sporotrichioides
 Fusarium longipes
100
 FIESC 12
83
 Stachybotrys chlorohalonata
100
78
 Stachybotrys chartarum 7711
 Stachybotrys chartarum 40293
 Stachybotrys chartarum 40288
100
100
 Myrotheicum roridum
 Trichoderma brevicompactum
 Trichoderma arundinaceum
 Microcyclospora tardicrescens
 Spicellum ovalisporum
100
 Spicellum roseum
 Trichothecium roseum K7-1
100
 Trichothecium roseum 197141
100
 Trichothecium roseum 195227
 Stachybotrys chlorohalonata
0.1
 Cordyceps confragosa UM487
87
100
 Cordyceps confragosa 1005
 Beauveria bassiana
94
89
 Fusarium longipes
87
 Fusarium sporotrichioides
 Fusarium graminearum
100
 FIESC 12
 Stachybotrys chartarum 7711
89
 Stachybotrys chartarum 40293
99
 Stachybotrys chartarum 40288
 Stachybotrys chlorohalonata
87
 Myrotheicum roridum
 Spicellum ovalisporum
100
 Spicellum roseum
 Trichothecium roseum K7-1
99
 Trichothecium roseum 197141
100
 Trichothecium roseum 195227
 Microcyclospora tardicrescens
 Trichoderma arundinaceum
 Trichoderma brevicompactum
100
 KND87290.1
0.1
 Cordyceps confragosa UM487
99
100
 Cordyceps confragosa 1005
 Beauveria bassiana
 Fusarium graminearum
 Fusarium sporotrichioides
 FIESC 12
100
 Fusarium longipes
95
 Stachybotrys chartarum 40288
100
97
 Stachybotrys chartarum 40293
 Stachybotrys chartarum 7711
 Stachybotrys chlorohalonata
88
 Myrotheicum roridum
98
 Spicellum ovalisporum
100
 Spicellum roseum
 Trichothecium roseum 195227
 Trichothecium roseum K7-1
97
 Trichothecium roseum 197141
98
 Trichoderma brevicompactum
 Trichoderma arundinaceum
85
 Microcyclospora tardicrescens
 XP 018177726.1
0.1
