# Supplementary material for: Evolution of structural diversity of trichothecenes, a family of toxins produced by plant pathogenic and entomopathogenic fungi
Source: PLoS Pathog. 2018 Apr 12;14(4):e1006946. doi: 10.1371/journal.ppat.1006946 (PMC5897003; doi:10.1371/journal.ppat.1006946)
Supplement: S8 Fig — (PPTX) [file ppat.1006946.s011.pptx]

## Slide 1
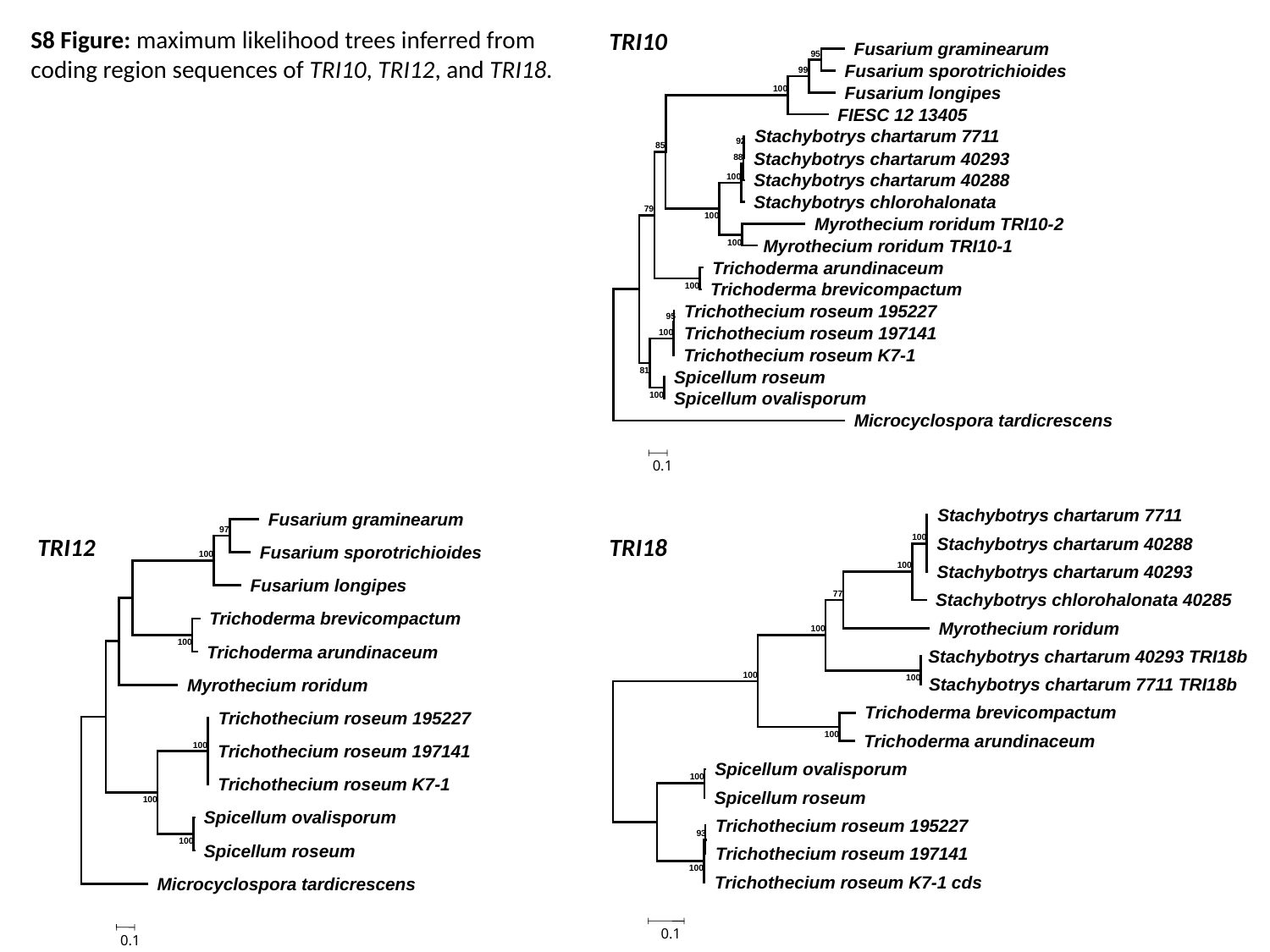

S8 Figure: maximum likelihood trees inferred from coding region sequences of TRI10, TRI12, and TRI18.
TRI10
 Fusarium graminearum
95
 Fusarium sporotrichioides
99
 Fusarium longipes
100
 FIESC 12 13405
 Stachybotrys chartarum 7711
92
85
 Stachybotrys chartarum 40293
88
 Stachybotrys chartarum 40288
100
 Stachybotrys chlorohalonata
79
100
 Myrothecium roridum TRI10-2
 Myrothecium roridum TRI10-1
100
 Trichoderma arundinaceum
 Trichoderma brevicompactum
100
 Trichothecium roseum 195227
95
 Trichothecium roseum 197141
100
 Trichothecium roseum K7-1
81
 Spicellum roseum
 Spicellum ovalisporum
100
 Microcyclospora tardicrescens
0.1
 Stachybotrys chartarum 7711
100
 Stachybotrys chartarum 40288
100
 Stachybotrys chartarum 40293
77
 Stachybotrys chlorohalonata 40285
 Myrothecium roridum
100
 Stachybotrys chartarum 40293 TRI18b
100
100
 Stachybotrys chartarum 7711 TRI18b
 Trichoderma brevicompactum
100
 Trichoderma arundinaceum
 Spicellum ovalisporum
100
 Spicellum roseum
 Trichothecium roseum 195227
93
 Trichothecium roseum 197141
100
 Trichothecium roseum K7-1 cds
0.1
 Fusarium graminearum
97
 Fusarium sporotrichioides
100
 Fusarium longipes
 Trichoderma brevicompactum
100
 Trichoderma arundinaceum
 Myrothecium roridum
 Trichothecium roseum 195227
100
 Trichothecium roseum 197141
 Trichothecium roseum K7-1
100
 Spicellum ovalisporum
100
 Spicellum roseum
 Microcyclospora tardicrescens
0.1
TRI12
TRI18
