# Supplementary material for: Evolution of structural diversity of trichothecenes, a family of toxins produced by plant pathogenic and entomopathogenic fungi
Source: PLoS Pathog. 2018 Apr 12;14(4):e1006946. doi: 10.1371/journal.ppat.1006946 (PMC5897003; doi:10.1371/journal.ppat.1006946)
Supplement: S1 Table — (DOCX) [file ppat.1006946.s012.docx]

**S1 Table:** oligonucleotide primers used in this study.

| **Primer designation** | **Nucleotide sequence (5’🡪3’)** |
| --- | --- |
| **Construction of *Trichoderma arundinaceum TRI3* deletion plasmid** | |
| Tarun-TRI35F | TCCGCGGATGGGTAGCAAACTTCCGGA |
| Tarun-TRI35R | AGATATCCAACCTCCCTTAACGGACAA |
| Tarun-TRI33F | TGATATCGCCTGGAGTTCAAATCTGGA |
| Tarun-TRI33R | AAGGCCTTCCCAACGCATCATACGCTT |
| **Analysis of *T. arundinaceum* *TRI3* deletion transformants** | |
| Tarun-TrpC3 | GTAACCATGCATGGTTGC |
| Tarun-CompT35F | CGGGGGAGAGAATAGTAA |
| **Construction of *T. arundinaceum TRI3* complementation plasmid** | |
| Tarun-TRI3F | CCATGGGTAGCAAACTTCCGGAA |
| Tarun-TRI3R | CCATGGCGCTTATTGGGAGAAGA |
| **Analysis of *T. arundinaceum TRI3* complementation transformants** | |
| Tarun-T3int3 | CCTCCTCCTGACTGTAAT |
| Tarun-T3int5 | TATTGAGGAGCTGCGAGA |
| Tarun-Phleo-3**^a^** | GGTGTTGGTCGGCGTCGG |
| Tarun-Phleo-4**^a^** | TGGGTGTGGGTGCGCGG |
| **Construction of *T. arundinaceum TRI17* deletion plasmid** | |
| Tarun-TRI1755X | TTTTCTCGAGGCGTCACTACTAACAGAG |
| Tarun-TRI1753EV | gTGTGATATCATGGAAGATGAACCAGCG |
| Tarun-TRI1735EV | GTGTGATATCAAGTGGGAGAGAGAACGA |
| Tarun-TIR1733Sc | TTTTGAGCTCGTTAATCTGATCGCTGGG |
| **Analysis of *T. arundinaceum* *TRI17* deletion transformants** | |
| Tarun-Db741 | GGATGCCTCCGCTCGAAGTA |
| Tarun-Db742 | CGTTGCAAGACCTGCCTGAA |
| Tarun-pks-F | TGCAAGCCTTATGTCAGTCG |
| Tarun-pks-R | GCTTAGCGGACGTTAGATCG |
| **Construction of *T. arundinaceum TRI17* for complementation plasmid** | |
| Tarun-TRI17NtP | AGCGGAACAAATCCCACC |
| Tarun-TRI17NtB | GCCTCATAGGTGGTCTCT |
| Tarun-TRI17CtBN | TTTTCCATGGCTGCGTCAGATACAGCAA |
| Tarun-TRI17CtN | TTTTCCATGGCCTTCACATCTAGGCTGT |
| **Construction of *Myrothecium roridum TRI17* complementation plasmid** | |
| Mrori-TRI17-Nt | AGCGGACCCAACCCCATC |
| Mrori-TRI17-Ct | TCAAGCCTTAACTTCAGGGGTAGTTG |
| **Analysis of *T. arundinaceum* *tri17* mutant complemented with *M. roridum TRI17*** | |
| Mrori-TRI17-4 | GGTGTTGTTGTTGCTGAG |
| Mrori-TRI17-5rev | GCTTCAAGAGCTTGCGAA |
| **Amplification and sequencing of *Trichothecium roseum TRI6* paralogs** | |
| Trose-TRI6aF | GATGATGGGAGAAAGAATGGAC |
| Trose-TRI6aR | CGTGCATGCGCTTGTAGTGGTC |
| Trose-TRI6bF | TCATCACGGACGCCAACACAC |
| Trose-TRI6bR | CCTRTAGTGGTCCCTCATGTTG |
| Trose-TRI6cF | GATCATGAGAGACGAATCTTCC |
| Trose-TRI6cR | CTATTTCTCGTGAATCCTGCGGTA |
| **Construction of *Aureobasidium pullulans TEF1* promoter::*Beauveria bassiana TRI4* fusion plasmid** | |
| Apul_TEFpromF(2279) | GGGTAGCAAACGGTGGTCAAA |
| Apul_TEFpromR (2280) | GATGCGGTTCATGTTTGACGGTGATGTATGGAAGAT |
| Bbas_Tri4F (2281) | CATACATCACCGTCAAACATGAACCGCATCATGTTGCA |
| Bbas_Tri4R (2278) | GAGAGTGAGCAAGTAGTAAGC |
| **Construction of *A. pullulans TEF1* promoter::*B. bassiana TRI22* fusion plasmid** | |
| Amp_Apul-Tefprom-F | GGTAGCAAACGGTGGTCAAAGG |
| Amp_Apul-Tefprom-R | CTATCCCGATGATGTCGGCAGGCATGTTTGACGGTGATGTATGG |
| BbasTri22-F | CATCACCGTCAAACATGCCTGCCGACATCATCGGGATAG |
| BbasTri22-R | GGTTCTGTACCGAGTAAGTAGC |
| Apul-Tef1prom-Nest-F | GATGGGAAACGAGGATTGGATG |
| BbasTri22-2-Nest-R | GAAACATCGACGAGGCTATAAG |
| ***B. bassiana TRI101* expression plasmid** | |
| BbasTri101-F (3151) | CAGATGGCGTTCAAAGACACA |
| BbasTri101-R (3153) | GCCCTAATTATCCACAAACTTTGC |
|  |  |
|  |  |

**^a^** These oligonucleotide were also used for the analysis of the *tri17* mutant complemented with *T. arundinaceum TRI17* and *M. roridum TRI17*.
